# Macroscopic quorum sensing sustains differentiating embryonic stem cells

**DOI:** 10.1038/s41589-022-01225-x

**Published:** 2023-01-12

**Authors:** Hirad Daneshpour, Pim van den Bersselaar, Chun-Hao Chao, Thomas G. Fazzio, Hyun Youk

**Affiliations:** 1 Kavli Institute of Nanoscience, Delft, The Netherlands; 2 Department of Systems Biology, University of Massachusetts Chan Medical School, Worcester, MA USA; 3 Department of Molecular, Cell, and Cancer Biology, University of Massachusetts Chan Medical School, Worcester, MA USA; 4 CIFAR Azrieli Global Scholars Program, CIFAR, Toronto, ON Canada

**Keywords:** Networks and systems biology, Stem cells, Cell signalling, Cell death

## Abstract

Cells can secrete molecules that help each other’s replication. In cell cultures, chemical signals might diffuse only within a cell colony or between colonies. A chemical signal’s interaction length—how far apart interacting cells are—is often assumed to be some value without rigorous justifications because molecules’ invisible paths and complex multicellular geometries pose challenges. Here we present an approach, combining mathematical models and experiments, for determining a chemical signal’s interaction length. With murine embryonic stem (ES) cells as a testbed, we found that differentiating ES cells secrete FGF4, among others, to communicate over many millimeters in cell culture dishes and, thereby, form a spatially extended, macroscopic entity that grows only if its centimeter-scale population density is above a threshold value. With this ‘macroscopic quorum sensing’, an isolated macroscopic, but not isolated microscopic, colony can survive differentiation. Our integrated approach can determine chemical signals’ interaction lengths in generic multicellular communities.

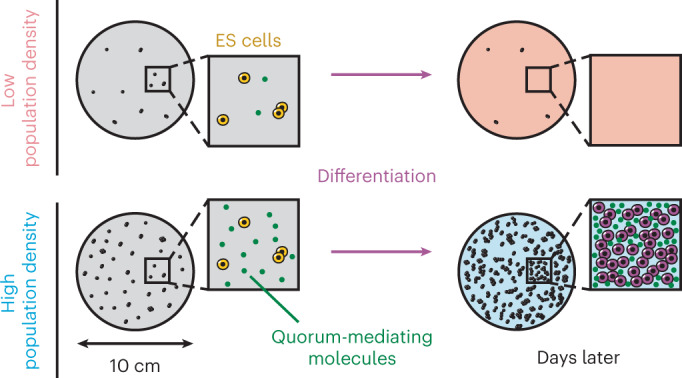

## Abstract (web summary)

Development of a quantitative approach to determine how far a signal from a cell travels as a diffusible molecule reveals a millimeter-scale quorum sensing mechanism that determines collective growth of differentiating embryonic stem cells.

## Main

A cell population grows when more cells divide than die per unit time. Cells often regulate their population’s growth by secreting a molecule that each cell uses for self-replication^1–4^. In an isogenic cell population, every cell can secrete the same replication-aiding factor (autocrine signal^3,5–7^). Thus, a cell can ‘self-communicate’ by secreting and capturing its own molecule before it diffuses to another cell^3,8^. A cell can also capture the same molecule but from another cell and, thereby, communicate with another cell^5–12^. This complication poses challenges for quantitatively understanding autocrine-based communication among cells^3,13–15^. These challenges include determining which cell secretes a particular molecule, which cell captures that molecule, how far the molecule diffuses and which intracellular pathways are regulated by each secreted molecule^15^. Even in the relatively simple context of cell culture dishes, it is often unclear whether cell–cell communication occurs only within a microscopic environment (within a colony), between sufficiently nearby colonies or over macroscopic distances (millimeters to centimeters) on the cell culture dish^14,15^. Conventionally, in cell cultures, one transfers conditioned media or uses microfluidics to determine whether autocrine signals exist^16–18^. But these methods either pool together all molecules from everywhere on the dish or directionally flows away all molecules, thereby erasing the crucial, spatial information about cell–cell communication. Resolving the above-mentioned challenges requires an approach that does not disturb the cells and paths of diffusing molecules that the cells use to communicate.

Here, we present a systematic approach in which we combine quantitative experiments and mathematical analyses of diffusion to address the challenges mentioned above for cell cultures. By applying this approach to a culture of mouse embryonic stem (ES) cells^16–28^, we discovered that ES cells quorum sense nearly at the centimeter scale as they start differentiating toward either the neural-ectodermal (NE) or mesendodermal (ME) lineages and that this macroscopic quorum sensing dictates whether the entire cell population, spanning centimeters on a dish, grows or suffers a catastrophic loss of viable cells. Our study provides a comprehensive, multi-scale analysis of a spatially extended and active, living matter^29,30^, thereby advancing understanding of how macroscopic living systems can stay out of thermal equilibrium^31–34^.

## Results

### Modeling autonomous and collective growths

**Fig. 1:**
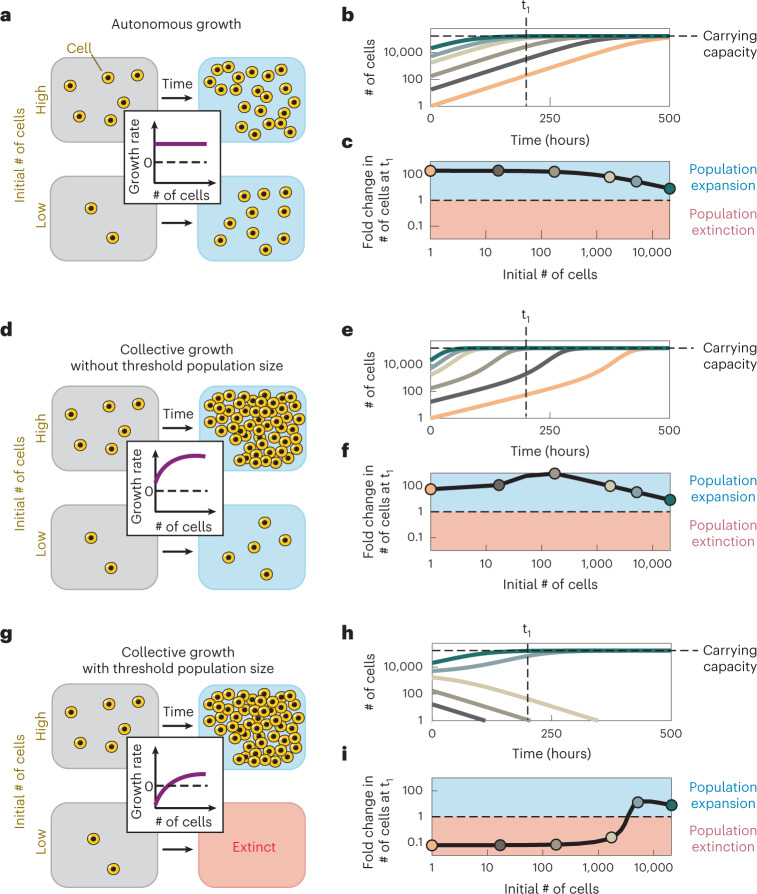
Phenomenological models for autonomous and collective growth of cells. **a**, Schematic of autonomously self-replicating cells, described by the logistic growth equation (Eq. 1 in Methods). Cell replication (cell growth) rate is independent of the population size (number of cells). **b**, Model-produced curves for autonomous growth (solutions of Eq. 1 in Methods). Horizontal dashed line: carrying capacity. Vertical dashed line: arbitrarily chosen time *t*_*1*_. **c**, Fold change in number of cells after time *t*_*1*_, read off from curves in **b**. Blue (red) shade: population approaches carrying capacity (extinction). **d**, Schematic of cells that collectively replicate without a threshold population size, described by Eq. 2 in Methods. Net growth rate is always positive. **e**, Model-produced curves for collective growth without a threshold population density (solutions of Eq. 2 in Methods). **f**, Fold change in number of cells after time *t*_*1*_, read off from curves in **e**. **g**, Schematic of cells that collectively replicate with a threshold population size, described by Eq. 2 in Methods. Net growth rate is negative for populations smaller than the threshold population density. **h**, Model-produced curves for collective growth with a threshold population density (solutions of Eq. 2 in Methods). Threshold population density is 3,400 cells per unit space. **i**, Fold change in number of cells after time *t*_*1*_, read off from curves in **h**.

To set the stage, we used mathematical modeling to consider three scenarios of population growths. In one scenario, cells autonomously replicate, causing cell populations to exponentially grow and reach a carrying capacity (Fig. 1a–c). Alternatively, cells can help each other replicate (collectively grow) by secreting molecules that increase each cell’s replication frequency. Here, the net growth rate (birth rate minus death rate) increases as the population size (number of cells) increases. If a cell can still autonomously replicate in its lifetime without any other cell’s help, every population still grows toward the carrying capacity, but a larger population grows faster due to each cell replicating more frequently (Fig. 1d–f). If a cell cannot replicate in its lifetime without help from sufficiently many (above-threshold) numbers of cells, the cell population can grow to the carrying capacity and avoid extinction only when the population size is above some ‘threshold population size’ (Fig. 1g–i).

### Sparsely seeding ES cells

**Fig. 2:**
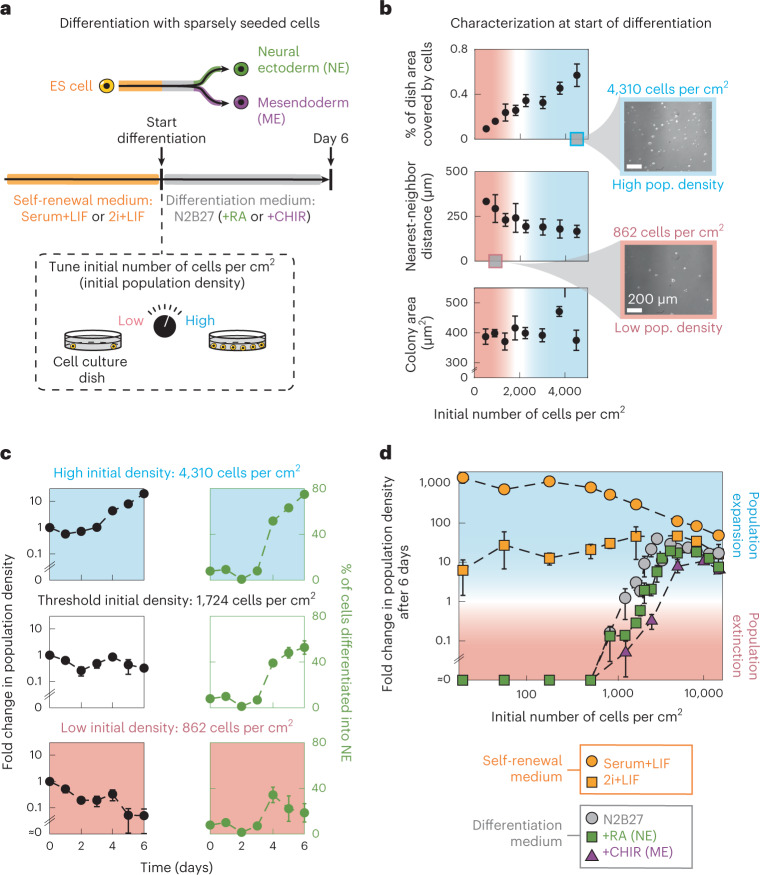
Differentiating cells, but not self-renewing cells, collectively grow with a threshold population density. **a**, Differentiation protocol (Methods). **b**, Characterizations of a 10-cm dish containing E14 cells, ~8 hours after seeding in N2B27, with wide-field microscope: percentage of dish covered by cells (top), average distance between nearest-neighboring colonies (middle) and average area of a microcolony (bottom). All data are presented as mean ± s.e.m. *n* = 3 biological replicates. Blue shade: population expansion regime. Red shade: population extinction regime. Shown for each regime: an example field of view in a dish (scale bar, 200 µm). **c**, Fold change in population density (black) and percentage of cells expressing Sox1-GFP (green) for RA-induced differentiation. Shown for different initial population densities (see also Supplementary Figs. 4–6). All data are presented as mean ± s.e.m. *n* = 3 biological replicates. **d**, Fold change in population density after 6 days in either one of two self-renewal media (orange) or one of three differentiation media (gray, green and purple). Cell lines: E14 (orange and gray), 46C (green) and Brachyury-GFP (purple). All data are presented as mean ± s.e.m. For ‘Differentiation medium: +RA (NE)’ condition: *n* = 4 biological replicates (green). For all other conditions, *n* = 3 biological replicates.

To determine which of the population growth scenarios fits murine ES cells in pluripotency (self-renewing state), we cultured ES cells with leukemia inhibitory factor (LIF) in a growth medium without serum (FBS), termed ‘2i’, or with serum (Fig. 2a). To determine the population growth scenario that describes differentiating ES cells, we cultured the cells without LIF in ‘N2B27’ medium, either without or with one of two inducers: the NE-inducing retinoic acid (RA)^35^ or the ME-inducing CHIR^36^ (Fig. 2a and Supplementary Fig. 1). Before adding either inducer, we kept ES cells in N2B27 for 2 days, during which they degrade pluripotency-maintaining factors^36^ (Methods).

To start the cell cultures, we used a flow cytometer and a hemocytometer to count ES cells in liquid suspension and then randomly scattered (seeded) them across a large (10-cm-diameter) cell culture dish (Fig. 2a and Supplementary Fig. 2). The resulting population density ranged from ~5 cells per cm^2^ to ~15,000 cells per cm^2^, and every dish had sparsely distributed, nearly isolated cells (Fig. 2b). With a wide-field microscope, we observed that cells covered less than 1% of every dish, and we measured the distribution of colony-to-colony distance for each dish (population density) (Fig. 2b). The average distance between two, nearest-neighboring colonies decreased as the seeded population density increased but was always ~100 μm or more (Fig. 2b). The initial colony area, on average, was virtually identical for every seeded population density. After seeding the cells on a dish, we incubated the dish at 37 °C, during which we left it untouched to not disturb diffusive paths of any molecules that the cells might be secreting. After some time, we determined the population density by detaching and counting all cells from the dish (Methods).

### Collective growth with threshold density in differentiation

We first examined populations of RA-induced, differentiating cells (46C cell line) that expressed GFP when they entered the NE lineage^37^ (Methods). Populations that began with a sufficiently high density (above ~1,700 cells per cm^2^) grew toward the carrying capacity, whereas populations that began with a sufficiently low density (below ~1,700 cells per cm^2^) approached extinction over 6 days (Fig. 2c and Methods). A population that nearly began with a ‘threshold density’ (~1,700 cells per cm^2^) neither noticeably grew nor shrank during the first 6 days (Fig. 2c). But, days later, it either grew toward the carrying capacity or shrank toward extinction (Supplementary Fig. 3). Specifically, by using an ensemble of many dishes that all started with the same density, we discovered that two populations that start with the same, near-threshold density can have different outcomes: one expanding and one becoming extinct. Despite these complex growth dynamics, we found that higher initial population densities led to higher fractions of cells becoming NE (GFP-expressing) cells (Fig. 2c and Supplementary Figs. 4 and 5).

The same population growth scenario (collective growth with a density threshold) occurred in three different, widely used cell lines— E14, 46C and Brachyury-GFP cell lines^38^—in N2B27 media with and without inducers (Fig. 2d and Methods). It did not matter whether the ES cells were self-renewing in 2i or serum medium before they began differentiating (Supplementary Fig. 6). Hence, from here on and unless we state otherwise, we will focus on the 46C cells that self-renewed with serum and then transferred to N2B27 with RA for differentiation. In contrast, self-renewing ES cells did not collectively grow: every population grew to the carrying capacity, consistent with the autonomous growth scenario (Fig. 2d).

### Secreted factors set threshold density for collective growth

**Fig. 3:**
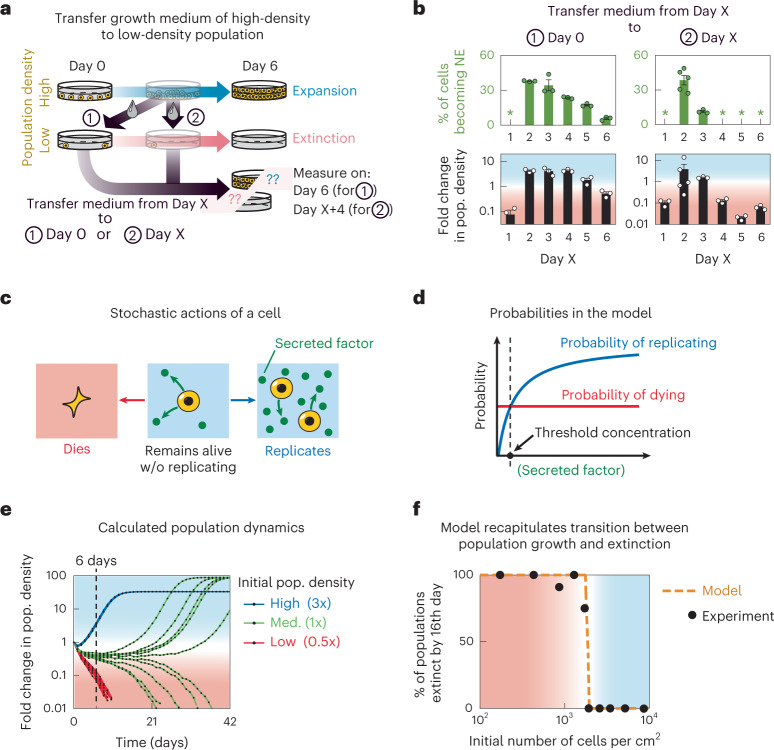
Stochastic model recapitulates collective growth with threshold population density. **a**, High-density population (initially 5,172 cells per cm^2^) expands (blue arrow) and low-density population (initially 862 cells per cm^2^) approaches extinction (red arrow). Two methods for replacing medium of the low-density population with medium (supernatant) of the high-density population. Method (labeled ‘1’): start differentiation by replacing low-density population’s medium with that of X-days-old high-density population. Method 2 (labeled ‘2’): replace medium of X-days-old low-density population with that of X-days-old high-density population. **b**, Results of medium transfer experiments. Percentage of cells that express Sox1-GFP (green bars) and fold change in population density (black bars). Left column: method 1 (*n* = 3 biological replicates for all data). Right column: method 2 (‘day 2’ has *n* = 5 biological replicates; all other days have *n* = 3 biological replicates). All error bars are s.e.m. Data for 46C cells in RA-induced differentiation. Asterisks: average fold change was ~0.1 or less (too few to reliably count Sox1-GFP cells in our flow cytometer). **c**, Stochastic model: three possible actions of a cell (Methods). **d**, Stochastic model: probabilities of cell replicating (blue; Eq. 3 in Methods) and of a cell dying (red; Eq. 4 in Methods). Dashed line: threshold concentration of survival factor. **e**, Simulations of the stochastic model (Eq. 8 in Methods) showing fold change in population density for various initial population densities: 862 cells per cm^2^ (red; low density), 1,931 cells per cm^2^ (green; near-threshold (medium) density) and 5,172 cells per cm^2^ (blue; high density). Ten simulated, replicate populations for each color. **f**, Orange curve: percentage of ten initially identical populations that become extinct by the 16th day of differentiation in the stochastic simulations. Each black point is from experiments with an ensemble of populations that had nearly identical initial population densities (data from Supplementary Fig. 3).

The above results suggest that differentiating cells secrete at least one ‘survival factor’ that promotes cell replications. If this is true, collecting the growth medium from a high-density population (5,172 cells per cm^2^) and then incubating in it an originally extinction-bound, low-density population (862 cells per cm^2^) should rescue the low-density population from extinction. We tested this idea in two ways (Fig. 3a). First, we collected the high-density population’s medium after X days of differentiation and then initiated differentiation of a low-density population in it (Fig. 3a). We found that only the media taken between the second and fifth day, but not earlier or later, rescued the low-density population (Fig. 3b). This suggests that the survival factor needs ~2 days to accumulate to a sufficient concentration and that it degrades over time in such a way that not enough of the factor exists for rescuing the low-density cells after ~6 days. In a second method, we collected the medium of the high-density population on Xth day of differentiation and then transplanted in it a low-density population that was already differentiating for the same number of days (X days) (Fig. 3a). We found that only the media taken from the second or third day rescued the low-density population (Fig. 3b). This suggests that, starting on the fourth day of differentiation, the survival factor is no longer effective.

### Stochastic model of collective growth with threshold density

The density threshold required for the collective growth may arise from requiring the survival factor to be above some ‘threshold concentration’ to promote enough cell births to counteract cell deaths. To test this idea, we built a stochastic model by modifying a previously built model that explained yeast collectively growing with a threshold population density at high temperatures^4^. The model assumes that a survival factor is secreted by every cell at the same, constant rate and that its concentration always remains spatially uniform (full details in Methods). In each timestep, a cell replicates, dies or remains alive without dividing (Fig. 3c). A cell’s probability of replicating non-linearly increases as the survival factor’s concentration increases, whereas the probability of a cell dying always remains constant, with the two probabilities matching at a ‘threshold concentration’ of the survival factor (Fig. 3d). To test the model, we used a wide-field microscope to continuously monitor differentiating cells for up to 4 days to measure the birth and death probabilities at each initial population density (Supplementary Figs. 7–9). The model’s only free (unmeasured) parameter was the threshold concentration. Regardless of the threshold value, the stochastic model yielded a collective growth with a threshold population density. By picking an appropriate value for the threshold concentration, the stochastic simulations yielded a threshold population density that matched the experimental value, experimentally observed fold changes for each population density and the experimentally observed ‘random outcome’ at the threshold population density, whereby two populations start with the same density but one becomes extinct, whereas the other one grows (Fig. 3e,f and Methods).

### Communication at millimeter scale for collective growth

**Fig. 4:**
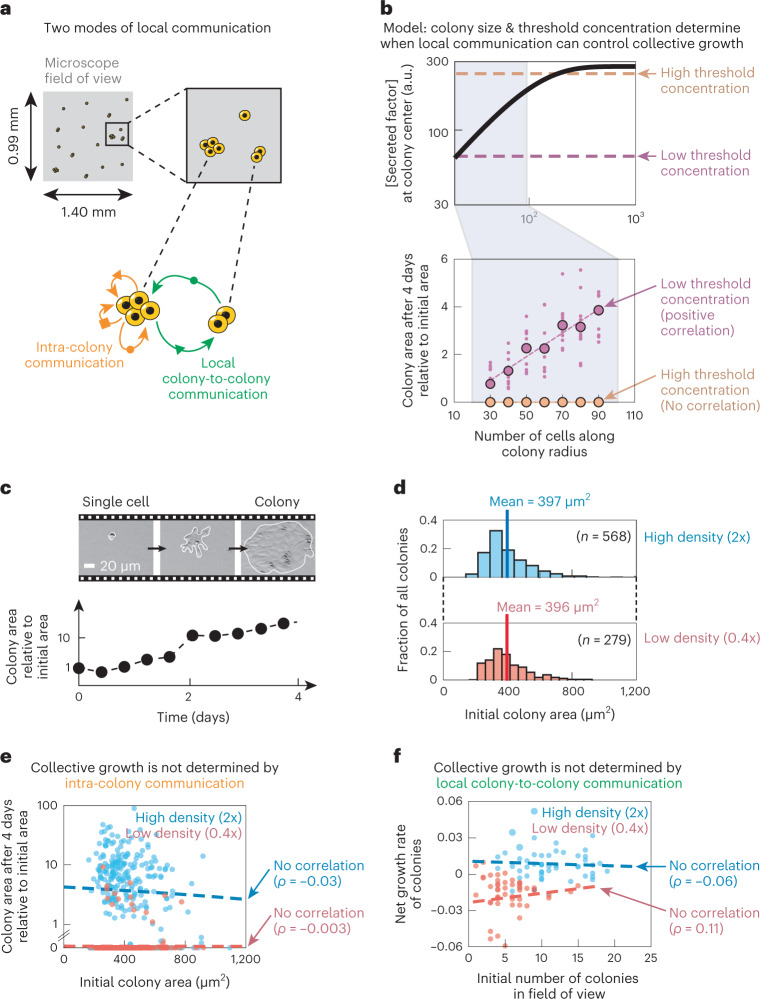
Communication below a millimeter scale cannot account for collective growth. **a**, Cartoon of microscope’s field of view (1.40 mm × 0.99 mm). **b**, Black curve: concentration of secreted, survival factor at circular colony’s center as a function of the colony size (Eq. (15) in Methods). Bottom graph: results from stochastic simulations (Eq. (8) in Methods). Small dot: result of one stochastic simulation. Large circle: averaged over all simulations. Purple (brown): simulations with a low (high) threshold concentration value as indicated in the top graph. Purple line in bottom graph: linear regression (Pearson correlation coefficient = 0.98). **c**, Example of 96-hour time-lapse movie of a growing microcolony (white outline drawn for visual guidance). Portion of a field of view is shown. Scale bar, 20 µm. **d**, Histogram of initial colony area (in µm^2^) for a high-density population (seeded 2,727 cells per cm^2^) that survives and expands toward carrying capacity (blue) and a low-density population (seeded 818 cells per cm^2^) that approaches extinction (red). Data from multiple fields of view, with E14 cells in N2B27. Mean colony area and number of colonies (*n*) are indicated. **e**, Blue dot: a microcolony from a high-density population (seeded 2,727 cells per cm^2^). Red dot: a microcolony from a low-density population (seeded 818 cell per cm^2^). The same microcolonies were analyzed in **d**. Microcolony’s area was measured 4 days after starting differentiation. Blue line: linear regression (correlation coefficient of −0.03). Red line: linear regression (correlation coefficient of −0.003). **f**, Blue and red data points represent the same microcolonies as in **e** but now show a microcolony’s net growth rate and, in the ~1-mm^2^ field of view that contains this colony, the number of microcolonies that initially existed. Blue line: linear regression (correlation coefficient of −0.06). Red line: linear regression (correlation coefficient of 0.11). a.u., arbitrary units.

Given the colonies in our microscope’s field of view (1.40 mm × 0.99 mm), we can consider two kinds of cell–cell communication with a survival factor: an ‘intra-colony communication’ between cells within the same colony and a ‘local colony-to-colony communication’ between two colonies in the same field of view (Fig. 4a). To understand both, consider a circular colony of a fixed radius. We can calculate the maximum possible number of cells, in two dimensions, that fit in the circle and then calculate the steady-state concentration of the survival factor that is sensed by the cell in the middle of the colony (‘center cell’) due to every cell in the colony secreting the survival factor at the same, constant rate (Methods). From these calculations, we find that the survival factor’s concentration at the center cell increases as the colony area increases but only up to a certain, maximum value. The maximum value occurs when the colony radius is near the survival factor’s diffusion length (Methods). Increasing the colony radius beyond the diffusion length cannot appreciably increase the survival factor’s concentration at the center cell (Fig. 4b). This occurs because the survival factors from cells that are more than the diffusion length away from the center cell degrade before reaching the center cell. The survival factor is most abundant at the center cell (Methods). So, if its concentration is less than the threshold concentration at the center cell, then it is also less than the threshold value everywhere in the colony, meaning that the entire colony is more likely to die (become extinct) than grow.

To further explore intra-colony communication’s implications, we re-examined our stochastic model by varying both the threshold concentration and colony radius (Methods). The simulations revealed that a colony’s growth is uncorrelated with its initial area when it is too small for generating an above-threshold concentration of survival factor at its center (Fig. 4b). We confirmed this by measuring areas of differentiating E14 cell colonies over 4 days in multiple, millimeter-scale fields of view (1.40 mm × 0.99 mm) (Fig. 4c and Supplementary Fig. 10). Initially, both high and low population densities (2,727 cells per cm^2^ and 818 cells per cm^2^, respectively) had virtually identical ranges of colony areas (Fig. 4d and Supplementary Fig. 11). The colony area, after 4 days, was uncorrelated with its initial area for both densities (Fig. 4e and Supplementary Fig. 12). Moreover, a relatively small colony in the low-density population would almost certainly die, but the same-sized colony in the high-density population would almost certainly grow (Fig. 4e). Hence, the threshold concentration must be higher than values achievable by colonies smaller than ~1,000 μm^2^ (~35-μm diameter).

The microscope-based time-lapse movies also revealed that a colony’s growth rate was independent of the number of colonies in a field of view at any population density (Fig. 4f and Supplementary Fig. 13). For example, in the low-density population, virtually every colony died in a field of view with ~10 colonies, whereas, in the high-density population, virtually every colony survived in a field of view with ~10 colonies (Fig. 4f). Moreover, a colony’s chance of surviving was uncorrelated with the distance between itself and its nearest-neighboring colony (Supplementary Fig. 14). These results establish that the threshold concentration must be higher than values achievable by any colony-to-colony communication that stays within a ~1-mm^2^ field of view. The survival factor must, thus, diffuse beyond 1 mm.

### Survival factor is suitable for long-distance communication

**Extended Data Fig. 1:**
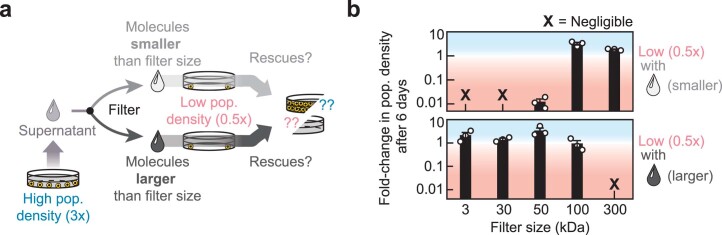
Survival factors diffuse by several millimeters to dictate collective growth. **a**, Protocol for separating secreted molecules in a supernatant, based on their molecular weights, by using membrane-based filters (Methods). For all experiments here, we used 46 C cells in N2B27 with RA. N2B27 medium is first taken from a high-density population (initially 5172 cells per cm^2^) after 2 days of differentiation. We then flow the medium through a membrane filter of a certain filter (pore) size, resulting in splitting of medium in two parts (bifurcating arrows): one part (follow arrows in top row) contains all molecules that are smaller than the filter size (in kDa) and a low-density population (initially 862 cells per cm^2^) is then incubated in this medium to test its growth. The other part of the filtered medium (follow arrows in bottom row) contains all molecules that are larger than the filter size (in kDa). Same procedure is carried out with this medium as with the medium containing all the lighter molecules. **b**, Results of experiment described in a. Fold changes in population density of the low-density population that was incubated in the medium which contained either all the molecules that were smaller than the filter size (top graph) or all the molecules that were larger than the filter size (bottom graph). Filter size is indicated on the horizontal axis. Cross (‘X’) indicates that the fold change is negligibly small (that is, average fold change < 0.01). Error bars are s.e.m.; *n* = 3 biological replicates.

A molecule’s diffusion length depends on its weight. To determine the survival factor(s)ʼ molecular weight(s), we filtered the differentiation medium (supernatant) of a 2-day-old high-density population (initially 5,172 cells per cm^2^) with a commercial ‘membrane filter’ that captures every molecule larger (heavier) than the membrane’s ‘filter size’ (specified in kDa) while letting through every smaller molecules^16^ (Supplementary Fig. 15). The filters make some errors so that molecules that are lighter or heavier by ~50% of the filter size can pass through or stay on the filter membrane (Methods). Into the filtered supernatant, which contains all molecules smaller than the filter size, we transplanted a 2-day-old, low-density population (862 cells per cm^2^) (Extended Data Fig. 1a). Only when the filter size was 50 kDa or larger, the transplanted low-density population expanded (Extended Data Fig. 1b). In another experiment, we filtered the supernatant, took all the molecules that were captured in the membrane and then dissolved them in N2B27. This medium, containing all molecules that were larger than the filter size, rescued the low-density population only when the filter size was 100 kDa or less (Extended Data Fig. 1b). Accounting for the filters’ ~50% error range, the above results establish that the survival factor(s) must be heavier than ~25 kDa and lighter than ~100 kDa.

**Extended Data Fig. 2:**
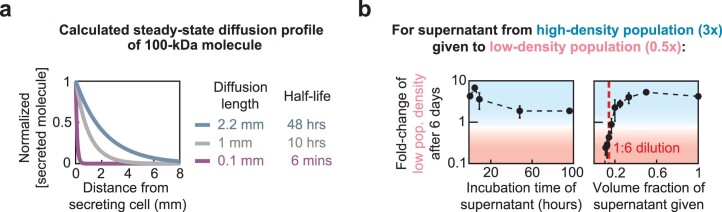
Millimeter-scale diffusion is enabled by days-long half-life of survival factors. **a**, Calculated concentration of secreted molecule of 100 kDa with three different half-lives (*t*_1/2_) and diffusion lengths *λ*. Calculations here show normalized, steady-state solution of the three-dimensional reaction–diffusion equation with the Stokes-Einstein relation for determining the diffusion constant (full details in Supplementary Notes). **b**, Experimental results showing that survival factors—all of them combined if more than one type exists—have an effective half-life of at least 2 days. Data shown for 46 C cells differentiating in N2B27 with RA. We took the growth medium (supernatant) of a high-density population (seeded 5172 cells per cm^2^) after 2 days of differentiation medium, and then incubated in it, a low-density population (seeded 862 cells per cm^2^). Before the transfer to the low-density population, we either incubated the supernatant, without any cells, at 37 °C (5% CO_2_) for various durations (left graph) or diluted it with fresh N2B27 by various amounts (right graph). More details in Supplementary Fig. 26. Error bars are s.e.m.; *n* = 3 biological replicates.

According to the Stokes–Einstein relationship, a molecule’s diffusion length *λ* is $\sqrt {D\tau }$, where *τ* is the molecule’s half-life. With the estimated weight range and for various values of *τ*, we determined *λ* and, by solving the reaction–diffusion equation, obtained a steady-state concentration gradient formed by the survival factor around a secreting cell (Extended Data Fig. 2a). Experimentally, we found that a high-density population’s supernatant, incubated at 37 °C without any cells for 2 days, had undiminished, growth-inducing effects (Extended Data Fig. 2b). Hence, the survival factor has a half-life of at least 2 days and, evidently, according to the steady-state concentration gradients computed above, diffuses over several millimeters.

**Fig. 5:**
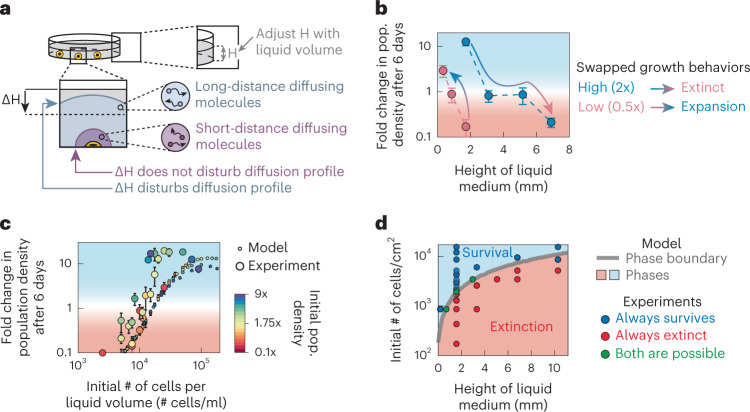
Communication over millimeters dictates collective growth. **a**, Diffusion profile for molecules with long-distance (blue region) or short-distance (purple region) diffusion. Cell (yellow) at dish bottom secretes both molecules. Δ*H*, change in liquid height. *H*, total height of liquid medium. **b**, Results of experiment in **a**. Red points: low-density population (seeded 862 cells per cm^2^). Blue points: high-density population (seeded 3,448 cells per cm^2^). Data for 46C cells in N2B27 with RA. Error bars are s.e.m.; *n* = 3 biological replicates. **c**, Data (large circles) for 46C cells in N2B27 with RA and stochastic simulations dictated by Eq. (8) in Methods (small circles), all from multiple volumes of growth medium (in milliliters: 2, 5, 10, 18, 20, 30, 40 and 60) and multiple initial numbers of cells per cm^2^ (172, 431, 862, 1,293, 1,724, 1,931, 2,155, 2,586, 3,017, 3,448, 4,310, 5,172, 6,034, 8,621, 9,483, 12,069 and 15,517, indicated by the color bar). Error bars are s.e.m.; *n* = 3 biological replicates for experiments (large circles); *n* = 10 stochastic simulations for model predictions (small circles). **d**, Phase diagram produced by data (circles; same data as in **c**) and stochastic simulations dictated by Eq. (8) in Methods (gray curve and blue-red shadings). Gray curve was constructed from the model by calculating, for each liquid medium height (volume), the threshold population density. Blue circles (‘Always survives’): conditions in which all replicate populations expanded. Red circles (‘Always extinct’): conditions in which all replicate populations decreased in their density during 6 days (fold change below 0.6). Green circles (‘Both are possible’): conditions in which some replicate populations expanded during 6 days while some did not.

If the survival factor indeed diffuses by millimeters, then its concentration gradient would change if we changed the height of the liquid culture medium by millimeters (Fig. 5a). Changing the height would not alter the two-dimensional population density. Consistent with this reasoning, we found that a low-density population (862 cells per cm^2^) grew toward the carrying capacity when the liquid height was 0.3 mm, whereas it became extinct when the liquid height was 2 mm (Fig. 5b). Conversely, a high-density population (3,448 cells per cm^2^) became extinct if the liquid height was 7 mm but survived if the height was 5 mm or 2 mm, with a faster growth in the 2-mm height than in the 5-mm height (Fig. 5b and Supplementary Fig. 16). Hence, differentiating cells secrete survival factor(s) that diffuse over at least ~5 mm.

Because the diffusion length is nearly 1 cm, we can assume that the survival factor is effectively uniformly mixed in our centimeter-scale dish. Indeed, when we widely varied both the initial population density and the liquid height, the resulting population growth dynamics in our experiments closely matched our stochastic model in which we assume that the survival factor is always uniformly distributed (Fig. 5c). Moreover, as a function of both the liquid height and initial population density, the stochastic model produced a boundary in the phase diagram, separating the population growth and population extinction phases, that closely matched the experimentally determined boundary (Fig. 5d and Supplementary Fig. 17). Thus, differentiating populations indeed quorum sense nearly at the centimeter scale.

### Macroscopic quorum sensing by secreting and sensing FGF4

**Extended Data Fig. 3:**
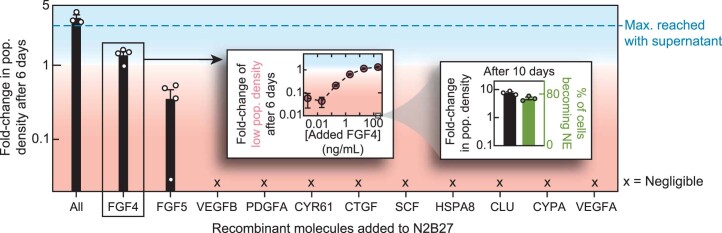
Screening of secreted molecules identified FGF4 as one of the secreted survival-factors. Main graph: fold change in population density, after 6 days in N2B27 supplemented with either one of the 11 recombinant molecules (indicated on horizontal axis) or all eleven molecules mixed (labeled ‘All’). Data are for a low-density population (862 cells per cm^2^) of 46 C cells in N2B27 with RA (see Methods and Supplementary Fig. 22 for full details and the concentrations for each molecule). Horizontal dashed line at top shows the fold change in population-density that occurs when the same low-density population grows in the supernatant of a high-density population (5172 cells per cm^2^) for 6 days. ‘X’ marks populations with negligibly small fold change (average fold change below 0.1). Middle inset graph: fold change in population density after adding various concentrations of recombinant FGF4. Same procedure as in the main graph. Right inset graph: fold change in population density (black bar) and percentage of cells entering NE lineage (Sox1-GFP expressing cells) (green bar), both measured 10 days after differentiation begins in the presence of 200 ng/ml FGF4 added at the start of differentiation. All error bars are s.e.m. All data are for *n* biological replicates as follows. *n* = 3 for ‘All’ and *n* = 4 for ‘FGF4’ and ‘FGF5’. For both inset graphs: *n* = 3 biological replicates.

To identify the survival factor(s), we performed RNA sequencing (RNA-seq) on high-density (5,172 cells per cm^2^), medium-density (1,931 cells per cm^2^; near threshold density) and low-density populations (862 cells per cm^2^) of 46C cells on their second day of differentiation in N2B27 without inducers (Supplementary Figs. 18 and 19). We identified 11 genes, expressed by all populations, which encode known, secreted ligands whose weights fall within the weight range that our membrane filters determined (Extended Data Fig. 3 and Supplementary Figs. 20 and 21). Of these, FGF4 was among the lightest molecules (~22 kDa). We incubated a low-density population in N2B27 supplemented with each of the 11 purified versions of the ligands (Methods). Of these, only the FGF4-supplemented media caused the low-density population to grow (Extended Data Fig. 3). Intriguingly, giving all 11 molecules to the low-density population caused it to grow the most, by nearly the same amount as in a supernatant of a high-density population (Supplementary Fig. 22). Hence, FGF4ʼs rescuing ability is likely enhanced by a combination of some of the ten other molecules. At least ~2 ng ml^−1^ (~0.13 nM) of FGF4 was required to rescue the low-density population (Extended Data Fig. 3). These results establish that FGF4 alone is sufficient for rescuing low-density populations from extinction.

**Fig. 6:**
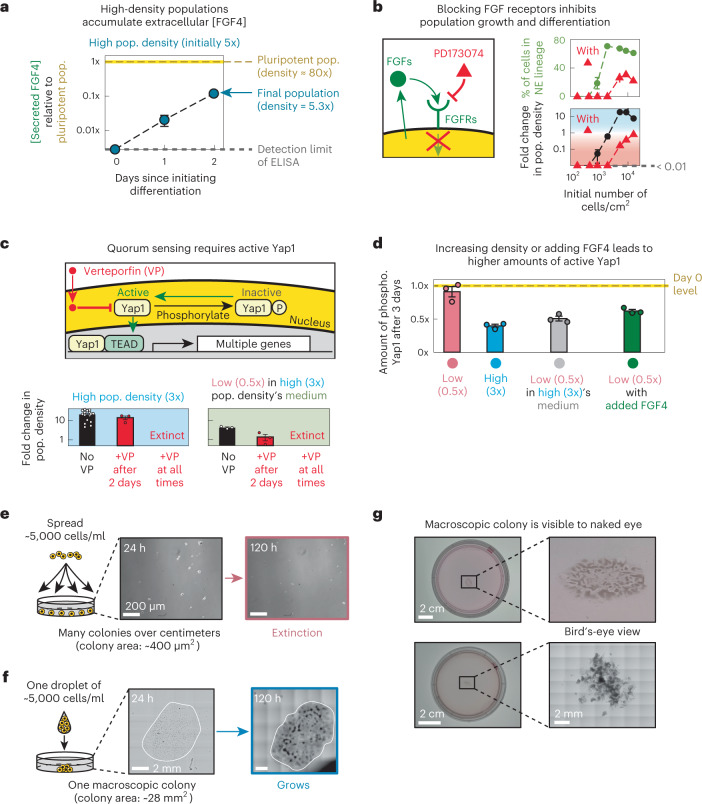
Macroscopic quorum sensing enables macroscopic colony to autonomously survive. **a**, Extracellular FGF4 concentrations, measured with ELISA, in N2B27 of a high-density population (seeded 8,620 cells per cm^2^) (Methods). Error bars are s.e.m.; *n* = 3 biological replicates. **b**, Cartoon: PD173074 inhibits FGFRs (Methods). Graphs: data from 46C cells after 6 days in N2B27 with RA. Red triangles: started differentiation with 2 µM of PD173074. Black and green points: differentiation without PD173074 but with the same DMSO concentration as for the red data points. Error bars are s.e.m.; *n* = 3 biological replicates. **c**, Cartoon: how Yap1 and VP function. Data, after 6 days of differentiation, for a high-density population (seeded 5,172 cells per cm^2^) and a low-density population that was rescued with supernatant from 2-day-old high-density population (seeded 862 cells per cm^2^). Error bars are s.e.m. All conditions show *n* biological replicates as follows. Black: *n* = 16 for high density, *n* = 3 for low density. Red: *n* = 3 for both densities. Last column: *n* = 4 for both densities. **d**, Normalized amount of phosphorylated Yap1 (inactive Yap1) measured with ELISA for 46C cells in N2B27 with RA (Methods). Densities are defined in **c**. Gray: low-density population rescued after 2 days by supernatant from 2-day-old high-density population. Green: low-density population starting with 200 ng ml^−1^ of FGF4. Error bars are s.e.m.; *n* = 3 biological replicates. **e**, Usual cell seeding protocol for a low-density population (~5,000 cells per ml). Representative images from *n* = 3 biological replicates. Scale bars, 200 µm. **f**, Seeding cells as one liquid droplet. Representative images from *n* = 3 biological replicates. Both images are from stitching together multiple fields of view. Each field is 1.40 mm × 0.99 mm. White outline: visual guide. Scale bars, 2 mm. **g**, Macroscopic colonies after 10 days in N2B27 with RA. Representative images from *n* = 3 biological replicates. Left column: pictures from a phone camera (estimated scale bar, 2 cm). Top row: 10-cm-diameter dish (left) and a zoomed-in view of a macroscopic colony (right). Bottom row: 6-cm-diameter dish (left) and a zoomed-in microscope image: stitched together multiple fields of view.

With ELISA, we detected a gradually increasing, extracellular concentration of FGF4 in N2B27 containing a high-density population (8,620 cells per cm^2^) (Fig. 6a). Consistent with this, we found that cells express *FGF4* and FGF receptors (*FGFR1–4*) during the first 2 days of differentiation (Supplementary Fig. 23). By supplementing N2B27 with PD173074, which inhibits the FGF receptors^21^ (*FGFR1–4*), we found that every population was driven to extinction, regardless of its initial density (Fig. 6b). Here, higher-density populations approached extinction more slowly, potentially due to having more FGF4s competing with PD173074 for the FGFRs.

### FGF4 diffuses by several millimeters

We found that FGF4 in N2B27 without any cells barely degraded at 37 °C over 3 days (Supplementary Figs. 24 and 25). Moreover, in a supernatant of a high-density population that was incubated without cells for 4 days at 37 °C, we found that a low-density population could grow by nearly the same amount as it would in fresh (unaged) supernatant from the same high-density population (Supplementary Fig. 26). According to these results, extracellular FGF4 is stable for at least 3 days. With this estimated half-life of FGF4 and its known weight (~22 kDa), the Stokes–Einstein relationship supports FGF4 diffusing over several millimeters (Methods). To confirm that FGF4 and any secreted factor primarily move by diffusion instead of other means such as convection in our cell cultures, we used a wide-field microscope to observe a droplet of dye spreading in N2B27 without any cells and concluded that diffusion alone can account for the speed at which the dye spreads (Supplementary Fig. 27).

### Quorum sensing with FGF4 activates Yap1

**Extended Data Fig. 4:**
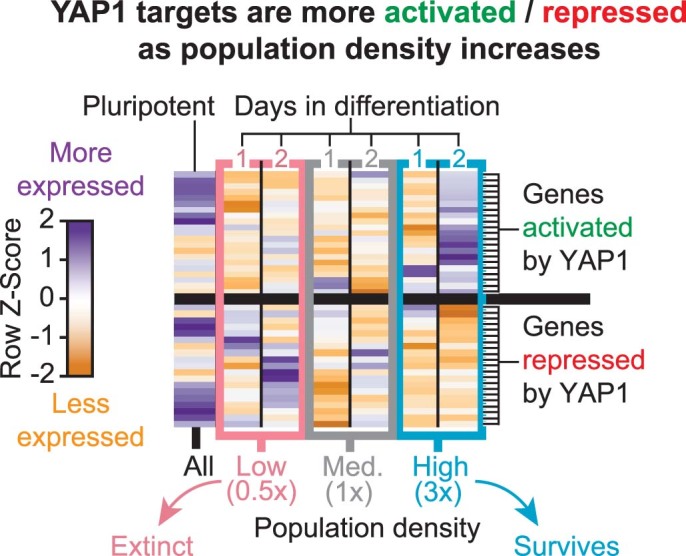
RNA-Seq reveals differential expressions of genes regulated by Yap1. Heat map showing transcriptome-wide changes in unguided differentiation (in N2B27) of 46 C cells (full details in Methods and gene labels for each row in Supplementary Fig 19). Low-density population (862 cells per cm^2^; enclosed in pink box). Near-threshold (medium-density) population (1931 cells/= per cm^2^; enclosed in gray box. High-density population (5172 cells per cm^2^; enclosed in blue box). Leftmost column shows data for self-renewing population just before differentiation begins (labeled ‘All’). Each column of differentiating population shows data for 1 day after (labeled ‘1’) or 2 days after (labeled ‘2’) starting differentiation. Each row shows a different gene, each of which are either activated (21 genes) or repressed (19 genes) by Yap1, either directly or indirectly. Supplementary Fig. 19 lists all genes. Color represents row Z-score: a measure of by how much a gene’s expression level for a given condition deviates from that gene’s expression level averaged across all different conditions (that is, different populations and days). Purple represents a positive row Z-score (more expressed than average). Orange represents a negative row Z-score (less expressed than average). Data from *n* = 3 biological replicates.

Yap1 is a master regulator of transcription for genes that control cell proliferation and differentiation and is primarily known for being regulated by cell-contact-mediated signaling^39–42^. The RNA-seq data revealed that the high-density population, compared to the low-density population, highly expressed genes that Yap1 either directly or indirectly activates (for example, *Cyr61* and *Amotl2*) and lowly expressed genes that Yap1 directly or indirectly downregulates^39–43^ (for example, *Angptl4* and *Tmem79*) (Extended Data Fig. 4 and Supplementary Fig. 19). Consistent with these observations, Yap1 was more active in higher population densities (Extended Data Fig. 4). Phosphorylating Yap1 inactivates it. Verteporfin (VP) is a small molecule that inhibits Yap1 (refs. 39,44) (Fig. 6c). Adding 1 µM of VP to the serum (FBS) medium did not affect the growth of self-renewing populations (Supplementary Fig. 28). Adding 1 µM of VP to N2B27 either with or without RA at the start of differentiation drove a high-density population (5,172 cells per cm^2^) to extinction, whereas adding it 2 days after starting differentiation did not affect the high-density population’s growth (Fig. 6c and Supplementary Fig. 28). As shown before, a low-density population (862 cells per cm^2^) grows in the supernatant from a 2-day-old, high-density population (Fig. 6c). Adding 1 µM of VP at the time of this medium transfer drove the low-density population toward extinction (Fig. 6c). But adding the VP 2 days after the medium transfer caused the low-density population to grow, albeit more slowly than when VP was always absent (Fig. 6c). These results suggest that the macroscopic quorum sensing requires activating Yap1.

### Collective Yap1 activation during the first 2 days

With phospho-ELISA, we measured the amount of phosphorylated (inactive) Yap1 at various population densities. The phospho-ELISA specifically detected phosphorylation at Ser397, the primary phosphorylation site of Yap1 (refs. 41,45). For each initial population density, we measured the amount of inactive Yap1 per cell, 3 days after starting differentiation, and in self-renewing cells. We found that higher population densities had less amounts of inactive Yap1 and that self-renewing cells had about the same amount of inactive Yap1 as the extinction-bound, low-density populations (Fig. 6d and Supplementary Fig. 29). Crucially, when we rescued a differentiating, low-density population by adding FGF4, cells had as much inactive Yap1 as the low-density population that we rescued with the medium from the high-density population (Fig. 6d and Supplementary Fig. 29). We also found that Yap1-regulated genes were upregulated in populations with abundant FGF4 and were lowly expressed in dying populations lacking sufficient FGF4 (Supplementary Figs. 30 and 31). These results establish that the macroscopic quorum sensing activates Yap1 and that Yap1 activation is necessary for population growth.

### Millimeter-sized colony can survive without other colonies

**Extended Data Fig. 5:**
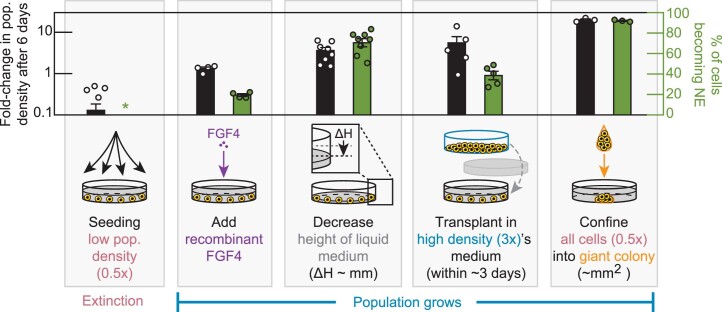
Strategies for inducing collective growth of differentiating ES cells. Bar graphs are for 46 C cells in N2B27 with RA. Black bars: fold change in population density after 6 days of differentiation. Green bars: percentage of cells expressing Sox1-GFP after 6 days of differentiation. All error bars are s.e.m. 1st column (causes ‘extinction’, meaning negligibly small fold change on average (see Methods)): spreading a relatively low number of cells (~5000 cells per ml) on a 10-cm-diameter dish (bar graphs for 58 cm^2^ of surface area (862 cells per cm^2^) and 10-ml of N2B27). Asterisk: too few cells to reliably measure percentage of cells expressing Sox1-GFP in our flow cytometer (both bars: *n* = 13 biological replicates). The following methods yield population expansion. 2nd column: adding recombinant FGF4 (200 ng/mL) to growth medium (both bars: *n* = 4 biological replicates). 3rd column: decreasing the height of growth medium by millimetres (both bars: *n* = 8 biological replicates). 4th column: transplanting low-density population into supernatant from a high-density population that had been differentiating for 2–3 days (both bars: *n* = 5 biological replicates). 5th column: seeding a macroscopic colony at the start of differentiation (both bars: *n* = 3 biological replicates).

Although spreading cells onto a dish could yield a ‘low-density population’ that became extinct (Fig. 6e), seeding the same total number of cells but now as one liquid droplet on a dish yielded a macroscopic colony that grew by ~tenfold during 6 days of differentiation with a characteristic length (diameter) of several millimeters (Fig. 6f,g). This result matches our model’s prediction: a sufficiently large colony can survive solely due to intra-colony communication. Among all our cell culturing methods, concentrating cells into a macroscopic aggregate yielded the highest differentiation efficiencies and fold changes in cell numbers (Extended Data Fig. 5).

## Discussion

We presented a systematic approach for determining the spatial range of diffusion-mediated interaction (communication) between cells on a culture dish. Determining this has been challenging even when one knew which molecules were involved^14,15^. Applying our approach to murine ES cell cultures revealed that a macroscopic quorum sensing mediated by several molecules, including FGF4, governs cell replications during differentiation. The macroscopic spatial scale of the quorum sensing is enabled by the weights and stabilities of the secreted, quorum-mediating molecules and by the long timescale (days) for differentiation, which gives the quorum-mediating molecules sufficient time to travel between two distant cells. The macroscopic quorum sensing entails that an isolated ES cell aggregate has to be sufficiently large to avoid becoming extinct during differentiation, which is relevant for building embryoid bodies and synthetic, embryo-like structures^26–28^.

Our models and interpretations come with several limitations and caveats because we constructed the simplest possible stochastic model—with the fewest possible parameters and simplest possible equations—that could explain our experiments (detailed in Methods). Notably, we neglected subpopulations of progenitors and already-differentiated cells that would gradually arise. These cells would divide and die at rates that differ from those of the undifferentiated cells that initially constitute the entire population. They may also survive without quorum sensing, as our data suggest (Fig. 2c). Studies have found that such subpopulations can affect population-level dynamics of differentiating cells^46–48^. Also neglected by our analyses is the fact that several quantities, including the threshold population density above which a population expands and the rate and efficiency of differentiation, can substantially differ among cell lines and differentiation conditions (Fig. 2d and Supplementary Fig. 6). These differences likely arise, in part, because each cell line and differentiation condition has its own cell-autonomous rates for death, division, differentiation and secretions of quorum-sensing molecules, all of which would also influence whether and how rapidly progenitors and differentiated cells arise. Finally, we used the reaction–diffusion equation to examine how molecules diffuse between cells, either in the same colony or different colonies, through the liquid medium in which the cells were immersed. But we neglected lateral diffusion of molecules through the crevices of cells within each colony, which is negligible for the microcolonies that we examined during early days of differentiation (Fig. 2b) but not negligible on later days when large colonies arise.

From a physics perspective, the quorum-sensing ES cells constitute a macroscopically active matter that avoids a decay to a thermal equilibrium (population extinction) due to reaction–diffusion processes that connect vast length scales. Our study provides a comprehensive, multi-scale analysis of a macroscopic biological system that extends understanding of how living systems stay out of thermal equilibrium and the quantitative principles that govern their dynamics^29–34,49,50^.

## Methods

### Deterministic models for autonomous and collective growth (in Fig. 1)

The purpose of the deterministic models in Fig. 1 is to explore qualitatively distinct ways in which a population can grow. The striking differences of the three growth types in Fig. 1 do not hinge on exactly what values we assign to the parameters in the model. Therefore, we did not use any experimental data to inform our choice of parameter values in these phenomenological models in Fig. 1 (these models also appear before any experiments in our paper). For all three models in Fig. 1, we assumed that cell populations grow at time *t* according to the logistic equation:

1 $$\frac{{dN}}{{dt}} = \left( {1 - \frac{N}{K}} \right)rN$$

where *N* is the number of cells; *K* is the carrying capacity (determined by the availability of nutrients and space); and *r* is the cells’ net growth rate (growth rate minus death rate). For autonomously replicating cells, the net growth rate can be any positive constant. For collectively growing cells—when cells help each other replicate by secreting and extracellularly accumulating molecules that uniformly mix in the environment—the net growth rate depends on the total number of cells. To capture the main, qualitative features of collective growth, we assume a simple, hyperbolic relationship between *r* and *N* as follows:

2 $$r\left( {N\left( t \right)} \right) = \frac{{\mu _{max}N\left( t \right)}}{{K_M + N\left( t \right)}} + r_0$$

Here, *µ*_*max*_ is the maximum net growth rate; *K*_*M*_ is the number of cells for which the population attains half of the maximum possible growth increase; and *r*_*0*_ is the autonomous net growth rate—net growth rate of an isolated cell—which can be either a positive constant (for collective growth without a threshold population size) or a negative constant (for collective growth with a threshold population size). We solved Eq. 1 for autonomous growth, and, for collective growth, we used MATLAB to numerically solve the system of equations formed by Eqs. 1 and 2. For each of the three population growth behaviors that we considered in Fig. 1, we used the following values to generate the series of curves shown in Fig. 1b,c,e,f,h,i: *N* (*t* = *0*) = 1; 17; 172; 1,724; 5,172; 20,690 (each shown in different colors). For all three population growth scenarios, we arbitrarily picked the parameter values. We used *K* = 172,414 for all three growth scenarios. For both types of collective growths, we used *µ*_*max*_ = 0.0519 and *K*_*M*_ = 3,500. For the autonomous growth scenario (Fig. 1a–c), we used *r* = 0.0264. For collective growth without a threshold population size (Fig. 1d–f), we used *r*_*0*_ = 0.02. For collective growth with a threshold population size (Fig. 1g–i), we used *r*_*0*_ = −0.0256. We used MATLAB to plot the number of cells (population size) between 0 and 500 hours (Fig. 1b,e,h). From the resulting graphs, we read off the fold change in the number of cells at an arbitrarily picked time *t*_*1*_ and then plotted it as a function of the initial population density (Fig. 1c,f,i).

### Stochastic model (in Figs. 3c–f, 4b and 5c,d)

Our stochastic model assumes that the secreted survival factor is always uniformly mixed (that is, every cell senses the same concentration of the survival factor). This assumption is justified by our experimental proof of the survival factor’s long diffusion length: the diffusion length is tens of times larger than the largest diameter that a colony can start with in our experiments (Fig. 2b and Supplementary Fig. 12b). The assumption is also justified by our experiments showing that both the average distance between two nearest-neighboring colonies (Fig. 2b) and the average distance between colonies (Supplementary Fig. 11) are an order of magnitude less than the diffusion length. Given these experimental findings, we assumed that every cell in a colony feels the same concentration of the survival factor—each colony is too small for an appreciable concentration gradient to form across it—and that every colony on a dish essentially feels the same concentration of the survival factor. In short, we can indeed assume that the survival factor is uniformly mixed for all practical purposes.

Our stochastic model is a minor modification of a similar stochastic model that we previously built to explain collective growth of yeast at high temperatures^4^. In fact, the quorum-sensing-based growth dyanmics of differentiating ES cell populations (Fig. 2c) is mathematically similar to the population growth dynamics of yeast cells at high temperatures (see Fig. 2 in Laman Trip and Youk^4^). Let us first describe the stochastic model in Fig. 3. Here, we assume that differentiating cells secrete a factor (survival factor) at a constant rate and that this factor also degrades at some constant rate. The survival factor can be any molecule that promotes cell replication. We assume that the probability of a cell replicating non-linearly increases as the extracellular concentration of the secreted factor increases (Fig. 3d, blue curve). The probability of a cell dying is constant: it does not depend on the concentration of the survival factor (Fig. 3d). Let *N*_*t*_ be the number of alive cells at time *t. t* takes integer values. At each timestep, cells die with a time-independent probability *P*_*γ*_ and replicate with a time-dependent (survival-factor-dependent) probability *P*_*μ*_(*t*). The extracellular concentration of the survival factor at time *t* is *M*_*t*_. *P*_*μ*_(*t*) is a non-linear function of *M*_*t*_ as follows:

3 $$P_{\mu}\left( t \right) = \mu \frac{{M_t}}{{K + M_t}}$$

where μ is the maximum possible probability of replicating, and *K* is the concentration at which the replication probability is half of its maximum value. The probability of a cell dying *P*_*γ*_ is a constant value *γ*:

4 $$P_\gamma = \gamma$$

is the ‘threshold concentration’ when *P*_*μ*_ = *P*_*γ*_. The survival factor’s extracellular concentration changes over time as follows:

5 $$M_{t + 1} = \frac{{N_t}}{V} + d\,M_t$$

where *V* is the volume of the liquid growth medium, and *d* is a degradation factor whose value is between 0 and 1. According to Eq. 5, *d* = 0 means that every molecule that currently exists (at time *t*) degrades in one timestep so that none of it is left (at time *t* + 1). When *d* = 1, no molecule ever degrades: the survival factor is permanently stable. When *d* is greater than 0 but less than 1, some fraction of the molecules degrades in one timestep. In Eq. 5, we measure the survival factor’s concentration *M*_*t*_ in units of the secretion rate. The secretion rate per cell is 1/*V*.

At each time *t*, we determine the number of cells that replicate, *R*_*t*_, which is a random variable that we pick from a binomial distribution in which each of the *N*_*t*_ cells has the same probability *P*_μ_(*t*) of replicating:

6 $$R_t\sim Binom\left( {N_t,P_\mu \left( t \right)} \right)$$

We also determine the number of cells that die, *D*_*t*_, which is a random variable that we pick from a binomial distribution in which each of the *N*_*t*_ cells has the same, constant (time-independent) probability *P*_*γ*_ of dying:

7 $$D_t\sim Binom\left( {N_t,P_\gamma } \right)$$

We then have one equation that governs the number of cells at each timestep:

8 $$N_{t + 1} = N_t + R_t - D_t$$

At *t* = *0*, we have *P*_*μ*_(0) = 0, as Eq. 3 indeed states, because the cell population begins without any survival factor (*M*_*0*_ = 0). To simulate Eq. 8, we can pick any positive integer for the initial number of cells, *N*_*0*_. The Methods subsection titled ‘Stochastic model for describing circular colonies in Fig. 4b’ explains how this model applies to a circular colony in Fig. 4b and gives an intuitive explanation of the stochastic, population-level dynamics that result from Eq. 8. The Methods subsection titled ‘Stochastic model’s limitations and caveats’ describes the limitations of the stochastic model.

### Parameter values for the stochastic model (in Figs. 3 and 5)

The stochastic model (Eqs. 3–8) has a total of five constant parameters. We experimentally determined the values for four of them—*V*, *d*, *P*_*γ*_ and *μ*—whereas we did not directly measure the fifth parameter, *K*.

We know *V* because we pipetted a known volume *V* of the growth medium into each cell culture dish (Supplementary Fig. 26). To experimentally determine the degradation factor *d*, we used FGF4-specific ELISA to measure the FGF4 concentration at different timepoints in a supernatant—taken from self-renewing ES cells—that we incubated without any cells at 37 °C (Supplementary Fig. 25). Based on this experiment and by letting each timestep in the stochastic model represent 1 hour, we concluded that *d* = 0.99. This high stability of a survival factor is consistent with another experiment in which we measured the effective half-life of all survival-promoting factors in a supernatant, including all unknown factors mixed with the secreted FGF4, by incubating a supernatant—taken from a high-density population—without any cells at 37 °C and then measuring its ability to rescue a low-density population from extinction (Supplementary Fig. 26). Thus, two different experiments support having *d* = 0.99.

To experimentally determine *P*_*γ*_, we used time-lapse microscopy to continuously monitor the areas of multiple colonies for 4 days, in multiple fields of views for each population density (Supplementary Figs. 7–10). In the resulting time-lapse movies, we observed cells and entire microcolonies dying and then lifting off the dish to float away at various timeframes. If we could count the total number of cells that have lifted off as a function of time in these movies, we would obtain the total number of dead cells, which increases as a function of time because it is a cumulative sum of the number of dying cells at each timeframe. But it was not feasible to count the integer numbers of dying cells at each timeframe of the movies. Instead, at each timeframe, we summed the area of each microcolony that detached from the plate. We then summed this number for each timeframe, yielding a cumulative, ‘total area of dead cells’ as a function of time. If we assume that there is a certain average area for a cell (average cell size), then the total area of dead cells by some given time would be directly proportional to the total number of cells that died up to that same time. With this reasoning, for each initial population density, we plotted the total area of dead cells as a function of time, which we fitted with an exponential function (~exp(−γt)) (Supplementary Fig. 8a). We found that *γ* was virtually identical for every initial population density (Supplementary Fig. 8b). We averaged *γ* over all initial population densities to obtain *P*_*γ*_ = 0.023 ± 0.002 h^−1^. We independently verified this value by measuring the net growth rate (birth rate minus death rate) of a population that started with the lowest density (~500 cells per cm^2^) in our time-lapse movie. This population hardly grew—as seen in a different experiment (flow-cytometer-based experiment in Fig. 2d)—and, thus, the net growth rate would be dominated by the death rate. The net growth rate for this population was a negative value, −0.026 h^−1^ (green circle in Supplementary Fig. 9c), meaning that this population’s death rate was approximately 0.026 h^−1^, which closely resembles the death rate found above (*P*_*γ*_ = 0.023 ± 0.002 h^−1^).

To experimentally determine the maximum growth rate *μ* in Eq. 3, we used the time-lapse movies mentioned above. In these movies, for each initial population density, we directly measured the total area of all colonies combined (colonies from multiple fields of view) at each timeframe (Supplementary Figs. 9 and 10). We fitted this value—the total combined area of all colonies—as a function of time with an exponential function (~exp(μ_net_t)), where μ_net_ is the net growth rate for a population of a given initial density (Supplementary Fig. 9a). We determined the net growth rate for every initial population density (Supplementary Fig. 9c). Because we determined the death rate *P*_*γ*_, which is the same for every initial population density, we can deduce the growth rate by adding the death rate to the net growth rate. The maximum growth rate *μ* is the maximum net growth rate plus *P*_*γ*_, which yielded *μ* = 0.052 h^−1^. We verified this value by another experiment: using the flow cytometer to count the number of cells over time in a population after dissociating all cells from a dish at each timepoint (Fig. 2c and Supplementary Figs. 2 and 4).

Finally, with the four parameter values—*V*, *d*, *P*_*γ*_ and *μ*—determined through the experiments mentioned above, we were left with just one parameter, *K* in Eq. 3, which we could freely tune. We chose *K* to be 4.85 × 10^5^ because then the stochastic simulations produced a threshold population density that closely matched the measured, threshold population density in Fig. 2c. Altering the value of *K* does not qualitatively alter the results of the stochastic model: the model still produces the collective growth with a threshold population density, which is the main phenomenon that we sought to recapitulate with our stochastic model.

### Intuitive description of the stochastic model (related to Figs. 3 and 5)

With the five parameter values assigned as explained above, we simulated Eq. 8 for each initial cell number, *N*_*0*_, for a wide range of values. Note that we used a fixed-volume *V* so we can use *N*_*t*_ to understand the population density, which is directly proportional to *N*_*t*_. For a sufficiently low *N*_*0*_, we have *P*_*μ*_(t) < *P*_*γ*_ at all times because the population, starting without any extracellular survival factor, does not have enough time to accumulate a sufficiently high concentration of the survival factor before all cells die. That is, the cells die too rapidly for the survival factor’s concentration to reach a value that is necessary for the replication probability to match and then go beyond the death probability *P*_*γ*_. Thus, an initially low-density population becomes extinction. For a sufficiently high *N*_*0*_, we eventually have *P*_*μ*_(t) > *P*_*γ*_ after some time, ensuring a sustained population growth, because *M*_*t*_ eventually becomes sufficiently high: it goes above the threshold concentration at which the probability of replicating equals *P*_*γ*_. For intermediate values of the initial population density, the survival factor’s concentration eventually, at time *t*, reaches a value such that *P*_*μ*_(*t*) ≈ *P*_*γ*_. Specifically, as the replication probability *P*_*μ*_ approaches the death probability *P*_*γ*_ from below, the population density remains nearly constant for some prolonged duration due to the replication probability nearly matching the death probability (that is, nearly equal numbers of cells replicate and die for some time) (flat parts of green curves in Fig. 3e). In this prolonged duration, stochastic births and deaths of a few cells determines whether the population can grow or head toward extinction. Namely, if just a few more cells die than replicate during the prolonged duration, the population loses its chance to accumulate an above-threshold concentration of the survival factor and, thereby, heads to extinction (descending green curves in Fig. 3e). In contrast, if just a few more cells replicate than die during the prolonged duration, the population grows due to the survival factor’s concentration becoming barely above the threshold, which then triggers a further growth of the population that, in turn, leads to a more rapid increase of the survival factor’s concentration beyond the threshold value (ascending green curves in Fig. 3e). By running simulations for various initial population densities (*N*_*0*_) and various volumes of growth medium (*V*), we determined a phase boundary that separates the population growth phase from the population extinction phase (gray curve in Fig. 5d). To calculate the phase boundary, we ran eight iterations of the stochastic simulation, based on Eq. 8, for each combination of *N*_*0*_ and *V* and then examined what percentage of these eight simulations led to a population growth toward the carrying capacity and how many of them led to a population extinction. In the population extinction phase (red region in Fig. 5d), all eight replica populations became extinct. In the population growth phase (blue region in Fig. 5d), all eight replica populations grew toward the carrying capacity. These determinations then allowed us to identify the boundary between the two phases (phase boundary) shown as a gray curve in Fig. 5d. At this boundary, the replication and death probabilities are identical (*P*_*μ*_ = *P*_*γ*_). Populations starting at this boundary exhibit the ‘random population-level growth’ seen as the green curves in Fig. 3e.

### Deriving a criterion that describes when intra-colony communication alone can dictate whether a population grows or not (in Fig. 4b)

The steady-state concentration of a three-dimensionally diffusing, survival factor at distance *r* from the center of a spherical cell that secretes it is:

9 $$c\left( r \right) = \frac{{c_RR}}{r}exp\left( { - \frac{{r - R}}{\lambda }} \right)$$

where *R* is the radius of the cell; $\lambda = \sqrt {D/\gamma }$ is the diffusion length; and *c*_*R*_ is the concentration on the cell surface (*r* = *R*). To measure *c* in units of *c*_*R*_, we consider:

10 $$\frac{{c\left( r \right)}}{{c_R}} = \frac{R}{r}exp\left( { - \frac{{r - R}}{\lambda }} \right)$$

Now, consider a series of identical, spherical cells lined up next to each other without any gaps in between them. Let us call the cell at the leftmost end of the line to be a ‘receiver cell’. The cell to its immediate right is called the ‘1st cell’; the cell to the immediate right of this cell is called the ‘2nd cell’ and so on. The distance *r*_*m*_ between the receiver cell’s surface and the center of the *m*-th cell is:

11 $$r_m = \left( {2m - 1} \right)R$$

where *m* ≥ 1. The normalized concentration created by the *m-*th cell on the receiver cell’s surface is:

12 $$\frac{{c\left( {r_m} \right)}}{{c_R}} = \frac{1}{{2m - 1}}{\it{exp}}\left( { - \frac{{2\left( {m - 1} \right)R}}{\lambda }} \right)$$

Consider a circular colony formed by spherical cells that are adhered to a surface. For simplicity, suppose that this colony consists of spherical cells that are arranged in a series of concentric circles. At the center of this colony is the receiver cell. It is surrounded by a series of rings (concentric circles) of radii *R* + *r*_*1*_, *R* + *r*_*2*_, *R* + *r*_*3*_ and so on. Let *N*_*m*_ be the total number of spherical cells that are arranged along the ring of radius *r*_*m*_. Then, *N*_*m*_ is approximately the total number of cell diameters (2*R*) that can fit on the circumference of the ring:

13 $$N_m \approx \frac{{2\pi \left( {R + r_m} \right)}}{{2R}} = 2m\pi$$

The total concentration *c*_*tot*_ of the survival factor on the receiving cell (colony’s center), due to the factor secreted by every cell in the colony other than the center cell itself, is:

14 $$\frac{{c_{tot}}}{{c_R}} = \mathop {\sum}\limits_{m \ge 1} {N_m\frac{{c\left( {r_m} \right)}}{{c_R}}}$$

which simplifies to:

15 $$\frac{{c_{tot}}}{{c_R}} = \mathop {\sum}\limits_{m \ge 1} {\frac{{2m\pi }}{{2m - 1}}exp\left( { - \frac{{2\left( {m - 1} \right)R}}{\lambda }} \right)}$$

Equation 15 shows that when *m* is large, the cells forming the ring of radius *R* + *r*_*m*_ contribute a negligible concentration at the colony’s center because the summand in the above equation approaches zero as *m* increases. Specifically, for sufficiently large *m*, we have:

16 $$\frac{{2m\pi }}{{2m - 1}}exp\left( { - \frac{{2\left( {m - 1} \right)R}}{\lambda }} \right)\sim exp\left( { - \frac{{2mR}}{\lambda }} \right)$$

Hence, a colony’s center cell receives negligible amounts of the survival factor from cells that are much farther away from it than the diffusion length *λ*. Now, suppose that cells are individually dispersed instead of being in a colony or that we have scattered islands (colonies) of cells instead of all cells being in a circular disc. Then, Eq. 16 shows that a cell would receive an appreciate amount of the survival factor from only the cells that are within the diffusion length *λ*. Only for such cells, we have 2*mR*/*λ* < 1, meaning that the contributed concentration, *exp* $( { - {\textstyle{{2mR} \over \lambda }}} )$, is non-negligible.

### Stochastic model for describing circular colonies in Fig. 4b

The stochastic model assumes that the survival factor is well mixed, but it still applies to the circular colonies in Fig. 4b. Here, every cell in a circular colony feels the same concentration of the survival factor. The entire colony grows if and only if *K* is sufficiently low such that the cells in the colony can generate an above-threshold concentration (the concentration at which *P*_*μ*_ = *P*_*γ*_) (purple dots in Fig. 4b). In contrast, the entire colony dies if and only if *K* is sufficiently high such that the cells in the colony cannot generate an above-threshold concentration (brown dots in Fig. 4b).

### Describing when intra-colony communication can control population growth (related to Fig. 4b)

Consider the total concentration *c*_*tot*_ in Eq. 15. *c*_*tot*_ alone does not dictate when an intra-colony communication alone is sufficient for dictating the survival of a colony. We must compare *c*_*tot*_ to the threshold concentration *c*_*thres*_ of the survival factor. In short, if *c*_*thres*_ is sufficiently low, then a small colony can generate *c*_*tot*_ that is larger than *c*_*thres*_. In this case, our stochastic model shows that a colony’s initial size would positively correlate with its probability of surviving. In other words, a colony’s initial size would positively correlate with its growth rate because the net growth rate—growth rate minus death rate—is proportional to the growth rate when the death rate is constant as is the case in the stochastic model. Having no correlation between the two quantities means that *c*_*thres*_ is sufficiently high, meaning that, no matter how large a colony is, its *c*_*tot*_ is always smaller than *c*_*thres*_. Since *c*_*tot*_ is larger for larger colonies, a sufficiently high *c*_*thres*_ means that our cell-seeding method cannot generate a colony whose initial area is sufficiently large that its *c*_*tot*_ is higher than *c*_*thres*_. Our data are consistent with this scenario: we do not observe any correlation between a colony size and its survivability (Fig. 4b and Supplementary Fig. 12). Note that if differentiating cells collectively grow *without* a threshold population size (Figs. 1d–f), then the above argument would not hold because, in this case, a larger colony would grow faster. Having a threshold concentration means that the outcome in binned into two categories—either growth (positive net growth rate) occurs or extinction (negative net growth rate) occurs—so that a larger colony is not more likely to survive than a smaller colony due to neither colony being large enough to generate an above-threshold value of *c*_*tot*_. Finally, note that a sufficiently large colony can survive based solely on its intra-colony communication (that is, even when the colony is by itself on a dish with no other colonies). Evidently, the colony sizes in our experiments are not sufficiently large. Being a sufficiently large colony means that its characteristic length (radius) is at least as large as the survival factor’s diffusion length. Otherwise, due to the exponential decay term, *exp* $\left( { - {\textstyle{{2mR} \over \lambda }}} \right)$, in Eq. 16, increasing the colony would negligibly increase the *c*_*tot*_. This reasoning is consistent with our experimental findings: the survival factor’s diffusion length λ is many millimeters while every colony on our dish had a characteristic length that was shorter than a millimeter. As another confirmation of this reasoning, we found that seeding a low number of cells as a single colony, by putting all the cells into a single liquid droplet that lands in the middle of the cell culture dish, leads to a sufficiently large colony—with a characteristic length of several millimeters (Fig. 6g)—that survives with intra-colony communication alone (note: there are no cells in the dish outside of this colony). Crucially, the same number of cells, if dispersed all over the dish through our usual seeding method instead of being seeded as a one liquid droplet, causes the seeded cell population to become extinct, consistent with the above reasoning. There may be contact-mediated (mechanically mediated) communication inside a colony that is also important for cell survival. Our analysis does not exclude this possibility. But if a macroscopic colony cannot survive on its own, as it does above, then our model for intra-colony communication would be incorrect regardless of an existence of contact-mediated communication that promotes survival.

### Stochastic model’s limitations and caveats

We sought to construct the simplest possible, stochastic model—with the fewest possible parameters and the simplest possible forms of equations—that could recapitulate the main observations from our experiments, including the phenomenon of collective growth with a threshold population density. Due to its simplicity, the stochastic model, although successfully recapitulating all the main features of our data, has limitations and caveats, which we describe here. We did not include subpopulations of progenitors or already-differentiated cells. Differentiated cells can affect population dynamics in complex ways that still lead to the population dynamics that our simple, stochastic model recapitulated. In fact, we expect differentiated cells to divide and die at rates that are different from those of undifferentiated cells. Hence, having a subpopulation of already-differentiated cells—given that not all cells differentiate at the same rate (Fig. 2c)—would affect the average death rate of cells over time, whereas we used a constant, average death rate in our stochastic model (Eq. 4). Moreover, our media transfer experiment (Fig. 3b) is consistent with cells that have already finished entering the NE lineage no longer needing the quorum sensing to survive, and, thus, these cells would form a subpopulation that dies and replicates at different rates from the undifferentiated cells that made up the initial population. This, too, would mean that our use of a single function for the probability of replicating *P*_*μ*_(*t*) and a single death probability *P*_*γ*_ were simplifications that still let our stochastic model recapitulate the main features of our data. Finally, differentiated cells may secrete FGF4 and other molecules differently from those that are not yet (fully) differentiated. Finally, differentiated cells may secrete FGF4 and other molecules differently from those that are not yet (fully) differentiated. There may be cell-to-cell feedback effects through (different) molecules secreted by already-differentiated cells or progenitors that form from ES cells that have not yet entered the NE or ME lineage^46–48^. Our experimental data do not exclude such complicated extracellular milieu, but it still evidently leads to the population-level growth features our simple, stochastic model could recapitulate. Our model uses stochastic, population-level variables rather than stochastic, single-cell-level variables. Broadly, there are two classes of stochastic models: a ‘population-level’ stochastic model, as in ours, and a ‘single-cell-level’ stochastic model that we have not used. Which one to use is a matter of a modeling strategy. We opted for the simpler of the two—a population-level stochastic model—because our primary goal was to describe the population size, which is the quantity that we directly measured, and build the simplest possible stochastic model that could recapitulate the main features of our data. Our stochastic model is a ‘population-level’ model in that it uses the number of cells (population size at time *t*) as the stochastic variable whose value changes at each timestep according to stochastic births, governed by the time-dependent *P*_*μ*_(*t*) in Eq. 3, and stochastic deaths, governed by the time-independent *P*_*γ*_ in Eq. 4. In this sense, not all cells behave the same way because, even though everyone ‘sees’ the same concentration *M*_*t*_ of the extracellular survival factor, not everyone divides and not everyone dies. At time *t*, every cell has probability *P*_*μ*_(*t*) of dividing, and this value depends on the molecule concentration *M*_*t*_, which is itself a stochastic variable because it depends on the stochastically determined number of cells at the previous timestep, *N*_*t−1*_. Because we assume that every cell at time *t* has the same chance of dividing and the same chance of dying, we used the binomial distribution that takes *P*_*μ*_(*t*) to determine the number of newborn cells at time *t* and the binomial distribution that takes *P*_*γ*_ to determine the number of dying cells at time *t*. An alternative to our population-level model would be a ‘single-cell-level’ model that can be implemented in numerous ways. For example, every cell can have a different probability of dividing at time *t*, unlike in our simpler model. To do so, each cell can have a different *P*_*μ*_(*t*) by, for example, having a different value of *μ* and/or different value of *K*. One can implement this model by distributing the *μ* among cells according to some probability distribution. Another, more complicated option is to have a different functional form of *P*_*μ*_(*t*) for each cell. As this discussion shows, there are myriad versions of single-cell-level stochastic models, and they are generally more complicated and require more drastic assumptions than those of our population-level stochastic model. For some instances of single-cell-level stochastic models, however, a sufficiently large number of cells—this may be as ‘small’ as 1,000—the central limit theorem may turn the single-cell-level model into a population-level stochastic model so that the total number of cells *N*_*t*_ is a random variable that follows some ‘simple’ distribution as in our model. We considered only one type of a secreted molecule. Although we discovered that FGF4 alone is sufficient, and necessary, for the quorum sensing, we also discovered that other factors enhance its action (Extended Data Fig. 3). Given the necessity of FGF4, we can consider the ‘molecule’ secreted in our stochastic model to be FGF4, but this is not required. Alternatively, we can consider the ‘molecule’ in our stochastic model to be a conglomerate of multiple factors, including FGF4, that are all secreted together in such a way that their concentrations are always directly proportional to one another (for example, factor ‘X’ is always twice the concentration of FGF4, and factor ‘Y’ is always one-third the concentration of FGF4). This direct proportionality occurs if we consider X, Y and FGF4 to be secreted at different rates but at some constant proportionality (for example, secretion rate of X is always some constant factor times the secretion rate of Y) and that X, Y and FGF4 all degrade at different rates but at some constant proportionality (can be different proportionality factors from the secretion rates). Our stochastic model allows for this interpretation of ‘conglomerate molecules’ because both the secretion rate and degradation factor are constants (that is, independent of time). Moreover, the degradation factor *d* in Eq. 5 is close to 1 because, from our experiments, we found that FGF4 and all the survival-promoting factors in the supernatant of high-density populations are stable for multiple days (Supplementary Figs. 25 and 26). Hence, the degradation factor *d* used in our stochastic model could have been just that of FGF4 or the effective degradation rate of all the conglomerate factors. Our stochastic model, however, cannot treat two survival factors secreted independently of each other (that is, a temporally varying proportionality factor between the secretion rates of the two molecules).

### Limitations and caveats of the reaction–diffusion analysis

To understand the effect of intra-colony communication on cell growth (Fig. 4b, top) and to calculate the diffusion coefficient *D* of secreted factors from our measurements, we used the three-dimensional, deterministic reaction–diffusion equation (Supplementary Eq. 1) and the Stokes–Einstein relationship (Supplementary Eq. 3). We also used other methods (experiments) to determine the diffusion length and the effects of intra-colony communication. These all agreed with calculations based on the reaction–diffusion equation. Still, one should note that the Stokes–Einstein relationship assumes that the diffusing molecule is spherical, which is an idealization. Moreover, we used the reaction–diffusion equation to describe diffusion through liquid growth medium, which contained our cells at the bottom (cells were adhered to the dish bottom). Although this is fine for describing the secreted factors diffusing from one cell to another cell in the colony through the liquid medium above them, we cannot use this equation to describe ‘lateral diffusion’: diffusion of the same survival factor in between the crevices of cells within the colony (for example, diffusion through complex extracellular matrix). We did not need to consider such lateral diffusion because the survival factor diffuses over many millimeters through the liquid medium—orders of magnitude longer than the diameter of any colony—and we experimentally demonstrated that any local (intra-colony) communication—without using any model to describe it—cannot explain the collective growth with the threshold population density.

### Cell lines and media

We used three murine ES cell lines: E14Tg2a.IV (129/Ola), 46C and Brachyury-eGFP. The 46C cell line (Sox1 promoter driving GFP) was previously described by Ying et al.^37^ and was a kind gift from Austin Smith. The Smith laboratory constructed this cell line by targeting GFP to the endogenous Sox1 locus. Thus, the 46C cells had the Sox1 promoter controlling a GFP expression. The Brachyury-eGFP cell line was previously described in Pearson et al.^38^ and was a kind gift from Valery Kouskoff whose laboratory constructed it by knocking in eGFP at the endogenous Brachyury (T) locus. Thus, this cell line had the Brachyury (T) promoter controlling an eGFP expression. To keep our ES cells pluripotent (in self-renewal), we passaged them in either a serum-based (FBS) or a serum-free (2i) pluripotency medium by tenfold dilution every 2 days. The serum-based medium (denoted ‘serum with LIF’ in our paper) consisted of high-glucose DMEM (Gibco, 11965092) supplemented with 15% FBS (Gibco, ES qualified, 11500526), 1× MEM non-essential amino acids (Gibco, 11140050), 1 mM sodium pyruvate (Gibco, 11360070), 1× GlutaMAX (Gibco, 35050061), 0.1 mM 2-mercaptoethanol (Gibco, 21985023), 1,000 U ml^−1^ of penicillin–streptomycin (Gibco, 15140122) and 1,000 U ml^−1^ of LIF (PolyGene, PG-A1140-0100). The serum-free medium (denoted 2i + LIF) consisted of approximately half-and-half mixture of Neurobasal (Gibco, 21103049) and DMEM/F12 (Gibco, 11320033) and was supplemented with 1× MEM non-essential amino acids (Gibco, 11140050), 1 mM sodium pyruvate (Gibco, 11360070), 1× GlutaMAX (Gibco, 35050061), 1× N-2 (Gibco, 17502048), 1× B-27 minus vitamin A (Gibco, 12587010), 0.1 mM 2-mercaptoethanol (Gibco, 21985023), 50 µg ml^−1^ of BSA (Sigma-Aldrich, fraction V, 10735094001), 1,000 U ml^−1^ of penicillin–streptomycin (Gibco, 15140122), 1,000 U ml^−1^ of LIF (PolyGene, PG-A1140-0100), 3 µM CHIR99021 (SanBio, 13122-25) and 1 µM PD0325901 (SanBio, 13034-25). We filtered all cell culture media with a 0.2-µm-pore bottle top filter. Cells were maintained in the self-renewal medium on 10-cm-diameter tissue culture dishes (Sarstedt, TC Dish 100 standard) that were coated with 0.1% gelatin in water (Sigma-Aldrich, from bovine skin type B, 10735094001) at 37 °C for at least 20 minutes before seeding cells. See Supplementary Table 1 for list of ingredients for the self-renewing and differentiation media.

### Dependence on cell lines and cell culture conditions for population growth and extinction

The phenomenon that we discovered—collective growth with a threshold population density—occurs for all three cell lines (E14, 46C and Brachyury-GFP) as shown in Supplementary Fig. 6 and Fig. 2. Moreover, this phenomenon occurs in three different kinds of differentiation media: N2B27 without any inducers, N2B27 with RA and N2B27 with CHIR (Supplementary Fig. 6 and Fig. 2). This phenomenon, occurring during differentiation, does not depend on how the ES cells were self-renewing before their incubation in one of the three differentiation media. Specifically, the cells could be self-renewing in serum-based (FBS with LIF) medium or serum-free (2i with LIF) medium. The experiments throughout our paper establish this independence on self-renewal media. Although the phenomenon holds for all three cell lines and three differentiation media, our data show that the type of cell line and the type of differentiation medium substantially affect the value of the threshold population density (Supplementary Fig. 6). Indeed, as our stochastic model shows, this value is a function of parameters such as maximal growth rate attainable by a cell and death rate of a cell (Eqs. 3 and 4), both of which can depend on the type of cell line, type of differentiation media and incubation condition (for example, CO_2_ and O_2_ levels), to name a few. Moreover, a possible scenario that we have not investigated is a subpopulation of differentiation progenitors, already-differentiated cells and ‘early’ differentiators not requiring the quorum sensing to grow and survive. Different cell lines and differentiation conditions would affect differentiation rate, which we did not attempt to explain with our model and analyses, and cell-autonomous rates, such as rates of growth and death, and parameters of the quorum-sensing architecture (for example, secretion rate and sensing of FGF4 and other quorum-sensing molecules). We did not investigate these complications and their impacts on how negative the net growth rate may become as the initial population density approaches 0 and, thus, at which low value, below 1, the fold change in population density plateaus after a sufficiently long time in differentiation medium. It is possible that, in some culture conditions, the threshold population density would be very low (much lower than the value we found). The lowest value that we found was ~500 cells per cm^2^ (purple curve in Supplementary Fig. 6). As our data show, the media type and cell line also affect the sharpness of the curve that describes the fold change in population density as a function of initial population density as well as how the curve tapers (plateaus) when the initial population density is below the threshold population density. We did not investigate these aspects. We classified a population to be expanding (growing) if its net growth rate (birth rate minus death rate) was positive so that the fold change in population density after some time was larger than 1. We classified a population to be approaching extinction (or simply that a ‘population became extinct’) if its net growth rate was negative in such a way that the fold change in population density was sufficiently below 1 after some time (Supplementary Fig. 9). For example, fold change of 0.1 was reached after some time for some populations that started with very low densities (for example, Supplementary Fig. 3). In our paper, ‘population extinction’ does not mean that there were absolutely no cells left on the cell culture plate. In fact, even a fold change of 0.1, for a 10-cm-diameter dish that started with a low density (~850 cells per cm^2^), still had many countable colonies. Indeed, there were still sufficiently many cells left for us to locate, count and report their abundance.

### Differentiation protocol

To initiate differentiation, we detached ES cells from gelatinized dishes with Accutase (Gibco, StemPro Accutase Cell Dissociation Reagent, A1110501). After collecting the detached cells, we washed them twice with 1× PBS and then centrifuged them to remove any remaining Accutase from the resulting cell pellet. We then resuspended the cell pellet in N2B27, which was prepared according to established protocols^38,51^. We then counted the number of cells per milliliter in this resuspension, as described in the ‘Cell counting’ subsection below. Afterward, we calculated the volume of this resuspension that was required to achieve a desired number of cells per cm^2^ on a dish and then pipetted this volume into a tube containing 10 ml of N2B27 that was pre-warmed to 37 °C. We then transferred this onto a 10-cm-diameter dish whose bottom was coated with 0.1% gelatin. We distributed (randomly scattered) the cells across the area of the dish by gently shaking the dish and then incubated the cells at 37 °C with 5% CO_2_. Importantly, we did not disturb the dish for at least 6 hours after the plating to allow the cells to sediment and attach to the gelatinized dish bottom. We defined this moment to be the start of differentiation. Cells were left for 2 days in the dish, and then the spent medium was replaced with either fresh, pre-warmed N2B27 (for unguided differentiation) or N2B27 supplemented with 500 nM of RA (SanBio, 11017-1) for NE differentiation or N2B27 supplemented with 3 µM of CHIR99021 (SanBio, 13122-25) for ME differentiation. We then left the dish for further incubation at 37 °C with 5% CO_2_. Subsequently, we collected the cells from plates for counting (see ‘Cell counting’ subsection below) and flow cytometry (see ‘Flow cytometry’ subsection below). Importantly, in accordance with published studies^38,51^, we verified that N2B27 without any inducers (RA or CHIR) caused a large majority of ES cells (more than 80%) to differentiate into the NE lineage, regardless of which medium (2i or serum) the ES cells were previously self-renewing in (Supplementary Fig. 1).

### Flow cytometry

We used Accutase to collect cells from a dish, washed the cells with 1× PBS, resuspended them in 1× PBS + 4% FBS and kept them on ice before flowing them into our flow cytometer. Our flow cytometer was the BD FACSCelesta system with a High-Throughput Sampler (HTS) and lasers with the following wavelengths: 405 nm (violet), 488 nm (blue) and 561 nm (yellow/green). We calibrated the forward scatter (FSC) and side scatter (SSC) gates to detect only ES cells (FSC-PMT = 231 V, SSC-PMT = 225 V, GFP-PMT = 476 V; as a control, flowing 1× PBS without cells yielded no detected events). We measured the GFP fluorescence using the FIT-C channel. Data were collected with BD FACSDiva 8.0 and analyzed with FlowJo 8.0 and a custom MATLAB script (MathWorks, R2016-R2020).

### Time-lapse microscopy

We used a wide-field microscope (Nikon, SMZ25) to continuously monitor microcolonies over days for various initial population densities (E14Tg2A cell line). Cells were cultured on a 6-cm-diameter tissue culture dish (Sarstedt, TC Dish 60, Standard) coated with 0.1% gelatin (Sigma-Aldrich, from bovine skin type B, G6650-100G) in water, fed with 4 ml of differentiation medium (N2B27) and incubated inside a temperature-controlled, CO_2_-controlled and humidity-controlled microscope chamber (Okolab) with steady conditions of 37 °C with 5% CO_2_. We imaged microcolonies with the following initial population densities (in number of cells per cm^2^): 455, 818, 1,227, 1,636, 2,045, 2,727, 3,409 and 4,091 cells per cm^2^ dish area. Previously, the cells were self-renewing in serum with LIF on 10-cm-diameter dishes and routinely passaged (every 2 days). Cells were given ~6 hours to settle down and attach to the gelatinized bottom of the dish before the image acquisition. The microscope’s mono-acquisition settings included a ×1 microscope objective, ×90.0 magnification, 28.5 (arbitrary units) of DIA intensity, 300-ms exposure time and ×2.2 analog gain. Before image acquisition, we picked multiple fields of view that were evenly spread across the entire 6-cm-diameter dish, with each field of view being 1,399.16 µm × 994.95 µm. Images were acquired with Andor IQ3. For each initial population density, we analyzed three biological replicates (three separate dishes), each in a different week and each consisting of multiple fields of view per dish. Images were acquired with 1-hour intervals in a total of 96-hour duration (that is, 4 days of imaging), during which cells were maintained in N2B27 without any inducer (unguided differentiation) without refreshing and, thus, disturbing cells. We analyzed the microscope images using a custom MATLAB script (MathWorks, R2016-R2020) and ImageJ 1.53c. We found, at most, ~10 colonies per field of view for the lowest population densities and, at most, ~50 colonies per field of view for the highest population densities.

### Cell counting

We counted cells with two devices—hemocytometer and flow cytometer—that both gave similar cell counts. To count the alive cells, we first detached all cells from a cell culture dish with Accutase and then washed them twice with 1× PBS. Then, in one method, we used a bright-field microscope (Motic AE31, ×100 total magnification) and a hemocytometer (Marienfeld Buerker, 631-0921) to count the alive cells by excluding dead cells with a trypan blue staining (dead cells appear blue). We counted the total number *N* of alive, non-stained cells in nine large squares, consistently excluding alive cells on two out of the four edges of each square. This way of counting enabled us to determine the total number of harvested, alive cells per unit of volume (ml) with the following formula: *N* × dilution factor × 10,000. In a second method, we used a flow cytometer to estimate the cell counts (see ‘Flow cytometry’ subsection and Supplementary Fig. 2).

### Medium-transfer experiments (in Fig. 3a,b)

We collected the liquid medium (supernatant) of a high-density population (5,172 cells per cm^2^), centrifuged it at 200*g* for 5 minutes to pellet and eliminate any remaining cells and debris from the supernatant and then transferred the supernatant to a low-density population (862 cells per cm^2^) after first removing the liquid medium of the low-density population. We did this in two ways. In one scenario (Fig. 3a, labeled as ‘1’), we collected the medium of a high-density population as described above on day X—the X here means X days after we initiated differentiation—and then incubated a low-density population in this medium to initiate its differentiation (that is, the low-density population was in a self-renewing medium before this). In the second scenario (Fig. 3a, labeled as ‘2’), we collected the medium of a high-density population as described above on day X and then incubated in it a low-density population that was differentiating for X days in its own medium. In this method, we measured the population density of the low-density population 4 days after the medium transfer (that is, X + 4 days) rather than on the same day for all values of X. This ensured that we could fairly compare the different low-density populations (that is, same number of days spent in the medium of the high-density population).

### Medium filtration experiments (in Extended Data Fig. 1)

We collected the liquid medium (supernatant) of a high-density population (5,172 cells per cm^2^) 2 days after we initiated its differentiation, centrifuged the supernatant at 200*g* for 5 minutes to eliminate any cells and debris from it and then transferred the supernatant to a second centrifugal tube for ultrafiltration. The filter unit consisted of two compartments that were physically separated by a regenerated cellulose membrane that separated soluble molecules, depending on their molecular size and shape. Specifically, the membrane had pores that either pass or hold soluble molecules based on their molecular weight (in kDa) during a high-speed centrifugation. We used filter sizes of 3 kDa (Merck, Amicon Ultra-15 Centrifugal Filter Unit, UFC900324), 30 kDa (idem, UFC903024), 50 kDa (idem, UFC905024), 100 kDa (idem, UFC910024) and 300 kDa (Merck, Vivaspin 20 centrifugal concentrator, Z629472). We centrifuged the supernatant of the high-density population for times specified by the manufacturer. After the filtration, the centrifugal tube with the membrane filter contained two supernatants, each in separate compartments: one that contained all molecules that were larger than the filter size—this portion was much less than 1 ml and stayed on top of the membrane filter—and one that contained all molecules that were smaller than the filter size. We added the supernatant containing larger-than-filter-size molecules to a 10-ml N2B27 with 500 nM of RA (for NE differentiation). In this mixed medium, we incubated a low-density population that had been differentiating, in N2B27, for 2 days. The results of this experiment are in the bottom graph of Extended Data Fig. 1b. In a second experiment, we added 500 nM of RA to the ~9 ml of the supernatant that contained all the molecules that were smaller than the filter size. We then incubated in it a low-density population that had been differentiating, in N2B27, for 2 days. The results of this experiment are in the top graph of Extended Data Fig. 1b. According to the manufacturer, to ensure that one captures proteins of a desired molecular weight, one needs to use a filter size that is at least two times smaller than the desired molecular weight. This sets a conservative safety/error margin that we incorporated into the conclusions that we drew from the results in Extended Data Fig. 1b, as explained in the main text. Finally, the few large molecules (>3 kDa) that are ingredients of N2B27 were previously shown to have either no effect or a small growth-promoting effect on ES cells (that is, they do not inhibit ES cell growth^16^). Additionally, we performed control experiments to confirm that the filters indeed did not catch any ingredients of the medium vital for cell growth (Supplementary Fig. 15). Moreover, we tested the accuracy of the filters in catching a specific molecule of known size by filtering a differentiation medium (N2B27) supplemented with recombinant LIF (1,000 U ml^−1^; 21.2 kDa according to manufacturer (PolyGene, ESLIF)) and then giving this filtrated medium to a low-density population (862 cells per cm^2^) otherwise bound to becoming extinct if not rescued. For this method, we measured the population density of the low-density population 6 days after giving either un-filtrated or filtrated N2B27 + LIF (filtrated with a 50-kDa filter size). This result ensured that we could fairly compare the different low-density populations.

### RNA-seq (in Extended Data Fig. 4)

We performed RNA-seq on differentiating 46C populations that were previously self-renewing in serum with LIF. We examined three initial population densities (number of cells per cm^2^): 862, 1,931 and 5,172. These populations were undergoing unguided differentiation (in N2B27 without any inducers), and we examined their transcriptome both 1 day after and 2 days after initiating their differentiation (Extended Data Fig. 4). We also performed RNA-seq on a pluripotent 46C population, which would represent the transcriptome of the three differentiating populations just before their differentiation began (Extended Data Fig. 4, first column). To perform the RNA-seq, we collected cells from each population (dish) and then centrifuged them using a pre-cooled centrifuge. We then extracted RNA (DNase-treated) from each cell pellet using PureLink RNA Mini Kit (Ambion, Life Technologies, 12183025) according to the manufacturer’s protocol. We next prepared the cDNA library with the 3′ mRNA-Seq Library Prep Kit (Quant-Seq, Lexogen) according to the manufacturer’s protocol. We measured the concentrations of each cDNA library before pooling using Quant-iT dsDNA Assay Kit (Invitrogen, Q33120) and a Qubit Fluorometer (Invitrogen). We then loaded the cDNA library pool onto an Illumina MiSeq system using the MiSeq Reagent Kit v3 (Illumina, MS-102-3001). We analyzed the resulting RNA-seq data as previously described in Trapnell et al.^52^. We performed the read alignment with TopHat 2.1.1. (also using Bowtie 2, SAMtools 1.16.1, BBDuk 36.85 and Salmon-1.5.1), read assembly with Cufflinks 2.2.1 and analyses of differential gene expression with Cuffdiff 2 and CummeRbund 2.7.2. As a reference genome, we used the genome sequence of *Mus musculus* from UCSC (mm10) (from https://genome.ucsc.edu/cgi-bin/hgGateway?db=mm10). We performed enrichment analysis of genes based on their FPKM values (that is, more than twofold expressed when two initial population densities are compared) by using Gene Ontology (GO) terms from PANTHER^53^, a custom MATLAB script (MathWorks, R2016-R2020) and RStudio 3.5.1. We visualized the results of pre-sorted, Yap1-related genes^39–45^ as heat maps using CummeRbund 2.7.2 and custom MATLAB scripts (MathWorks), which displayed the normalized expression value (row Z-score) for each gene and each condition. Supplementary Data 1 lists all genes examined in the RNA-seq.

### RT–qPCR

We performed RT–qPCR on differentiating 46C populations that were previously self-renewing in serum with LIF. We examined two initial population densities (number of cells per cm^2^): 862 and 5,172. We performed RT–qPCR on them every day for 4 days of differentiation in N2B27 with 500 nM of RA (for NE differentiation). We collected the cells and extracted their RNA with PureLink RNA Mini Kit (Ambion, Life Technologies, 12183025) according to the manufacturer’s protocol. Then, we reverse transcribed (DNase-treated) RNA into cDNA using iScript Reverse Transcription Supermix for RT–qPCR (Bio-Rad, 1708840). Next, we performed qPCR in 10-µl reactions with iTaq Universal SYBR Green Supermix (Bio-Rad, 172-5121) and 100 nM of forward and reverse primers. We verified the primer specificity and primer dimer formation by using the melt curve analysis, which showed one peak. See the list of primers that we used in Supplementary Table 2. On each day, we normalized a population’s gene expression level by that population’s *GAPDH* (housekeeping gene) level. Afterward, we compared each population’s *GAPDH*-normalized gene expression level for a given day to that of 1-day-old low-density population (whose value is, thus, ‘1×’ in Supplementary Fig. 30). We performed all reactions in triplicates on a ‘QuantStudio 5’ Real-Time PCR System (Thermo Fisher Scientific).

### Population rescue experiments with recombinant proteins (in Extended Data Fig. 3)

We examined whether we could rescue a low-density population from extinction by adding one or more molecules among 11 different autocrine-signaling molecules (all recombinant versions from mouse or human). We considered differentiating 46C cells that were previously self-renewing in serum with LIF in a low-density population (initially 862 cells per cm^2^). After 2 days of culturing in N2B27, we added 500 nM of RA and one or combinations of the following recombinant proteins to this medium: 200 ng ml^−1^ of recombinant mouse FGF4 (R&D Systems, 7486-F4), 200 ng ml^−1^ of recombinant human FGF5 (R&D Systems, 237-F5), 100 ng ml^−1^ of recombinant mouse PDGFA (Novus, NBP1-43148), 100 ng ml^−1^ of recombinant mouse VEGFB 186 (Novus, 767-VE), 100 ng ml^−1^ of recombinant mouse VEGFA (Novus, 493-MV), 500 ng ml^−1^ of recombinant human CYR61/CCN1 (Novus, 4055-CR), 500 ng ml^−1^ of recombinant human CTGF/CCN2 (Novus, 9190-CC), 200 ng ml^−1^ of recombinant mouse CLU (Novus, 2747-HS), 500 ng ml^−1^ of recombinant human HSPA8/HSC70 (Novus, NBP1-30278), 1,000 ng ml^−1^ of recombinant human cyclophilin A (PPIA) (Novus, NBC1-18425) or 2,000 ng ml^−1^ of mouse recombinant SCF (STEMCELL Technologies, 78064). After incubating in a medium containing one or a combination of these molecules for 4 days, we collected the cells for counting (see ‘Cell counting’ subsection) and flow cytometry (see ‘Flow cytometry’ subsection) to determine whether the population survived or not and its differentiation efficiency. Results of these experiments are in Supplementary Fig. 22.

### FGF4 ELISA (in Fig. 6a)

We measured concentrations of FGF4 in 10 ml of liquid media (N2B27) as follows. We used Mouse FGF4 ELISA Kit (ELISAGenie/Westburg, MOES00755) and followed the manufacturer’s protocol. The assay involved measuring the absorbance at 450 nm for various samples as a direct measure of the FGF4 concentration in the sample. We verified that the absorbance signals are real and sufficiently high relative to the lower detection limit of the ELISA kit by constructing a standard curve (Supplementary Fig. 24). We measured the absorbances on a Synergy HTX Multi-Mode Reader (BioTek). Following the manufacturer’s protocol, we diluted the stock of the biotinylated detection antibody by 1 in 100 to obtain a working stock. We normalized the ELISA measurements of FGF4 concentration by comparing it to that of a highly confluent population of ES cells in self-renewal media. The latter is expected to have a high concentration of extracellular FGF4, based on previous studies’ finding that pluripotent ES cells highly express FGF4 (refs. 21,54). Normalizing all our ELISA measurements of FGF4 concentration by that of the pluripotent population also makes our result interpretable if ELISA does not detect 100% of all FGF4s that are secreted (for example, due to antibodies not binding to all their targets) (see Supplementary Fig. 24 for validations of these justifications). With these justifications in mind, we used ELISA to measure the concentration of extracellular FGF4 in the medium of a high-density population (‘5×’ = 8,620 cells per cm^2^) during the first 2 days of differentiation (Fig. 6a and Supplementary Fig. 24). The 46C cells were differentiating for 2 days in N2B27 without any inducers and were previously self-renewing in serum with LIF. We also used ELISA to measure the concentration of extracellular FGF4 in the medium of a highly confluent population of pluripotent cells (population density of ~80×) (Fig. 6a, yellow line). The differentiation medium did not initially have any detectable amounts of FGF4 (Fig. 6a, ‘day 0’). By using three different forms of FGF4—one from the ELISA kit, FGF4 secreted by the cells in our experiments, and a recombinant form of FGF4 from a different company (R&D Systems, 486-F4)—we found that the ELISA kit did not detect all forms of mouse FGF4 but that it did detect the form of FGF4 secreted by our cells, although less efficiently than for the recombinant FGF4 that came with the ELISA kit (Supplementary Fig. 24).

### Phospho-Yap1 ELISA (in Fig. 6c,d)

We examined the endogenous levels of phosphorylated Yap1 protein in four different conditions (Fig. 6c,d and Supplementary Fig. 29). We examined 46C cells that were differentiating for 3 days in N2B27 with 500 nM RA and were self-renewing in serum with LIF before differentiating. For each measurement, we collected cells in 10-ml tubes, counted the total number of collected cells with the counting method described in the ‘Cell counting’ subsection and then centrifuged them to form a pellet. We then lysed the cells with a lysis buffer (Cell Signaling Technology, 9803) and 1 mM of PMSF (Sigma-Aldrich, P7626). We performed a sandwich-ELISA assay by incubating the cell lysates with Phospho-YAP (Ser397) rabbit antibody from PathScan Phospho-YAP (Ser397) Sandwich ELISA Kit (Cell Signaling Technology, 57046). We followed the manufacturer’s protocol for the assay. Following this protocol, we used a 1-in-10 dilution of the detection antibody (that is, first, we made a concentrated stock by dissolving the lyophilized antibody in 1.0 ml of the ‘detection antibody diluent’ from the kit; we then diluted this stock into 10.0 ml of the detection antibody diluent to obtain the working stock). We used a Synergy HTX Multi-Mode Reader (BioTek) to measure each sample’s absorbance at 450 nm. The absorbance is a direct measure of the abundance of phosphorylated Yap1. We constructed a standard curve by serially diluting a lysate of pluripotent cells (Supplementary Fig. 29). We used the standard curve to report the levels of phosphorylated Yap1 in all differentiating populations.

### Caspase-3 assay

We measured the activity of Caspase-3, a well-known apoptosis executioner, in E14 cells that were self-renewing in serum with LIF or were differentiating in N2B27 with 500 nM of RA (after self-renewing in serum with LIF). We examined three differentiating populations: a high-density population (6,896 cells per cm^2^ initially), a low-density population (517 cells per cm^2^ initially) and a low-density population that was rescued by the medium of the high-density population after 2 days of differentiating. We collected the cells of each of these populations and then performed a membrane-permeable DNA-dye-based assay that measures the amounts of active Caspase-3/7 in intact, alive cells (NucView 488 Caspase-3 Assay Kit for Live Cells, 30029). We followed all steps according to the manufacturer’s protocol. We used a flow cytometer to measure the amounts of active Caspase-3 in single cells. We normalized the Caspase-3 level per cell by the average Caspase-3 level of an ES cell (that is, mean level per cell of the pluripotent population.) Results of these experiments are in Supplementary Fig. 31.

### Inhibiting FGF receptors (in Fig. 6b)

We examined the fold changes in population densities after several days of inhibiting their FGFRs with a small-molecule inhibitor, PD173074 (PD17, Tocris, 3044) (previous studies characterized this inhibitor^21,55^). We used 46C cells that were differentiating in N2B27 + RA (RA added on day 2) and self-renewing in serum with LIF before the differentiation. We examined the following initial population densities (number of cells per cm^2^): 172, 431, 862, 1,931, 5,172, 8,620 and 15,517. To inhibit the FGFRs, we added 2 µl of 10 mM PD173074 (PD17) to a 10-ml N2B27 medium. We dissolved the stock of PD17 in DMSO to a final concentration of 2 µM (1,056 ng ml^−1^). After 6 days, we measured each population’s density (see ‘Cell counting’ subsection) and differentiation efficiency (see ‘Flow cytometry’ subsection). As a control, we examined the effect of adding 2 µl of DMSO to cell culture medium without any PD17. This ensured that our results were not due to any side effects of having DMSO that was carried over with the PD17 that we added to each medium. Results of these experiments are in Fig. 6b.

### VP experiments (in Fig. 6c)

We examined fold changes in population densities after several days of incubation with VP (R&D Systems, 5305), which prevents active Yap1 from entering the nucleus to regulate the transcription of multiple genes. We used 46C cells that were differentiating in N2B27 with and without RA (RA added on day 2) and that were self-renewing in either serum with LIF or 2i + LIF before the differentiation. We supplemented the differentiation medium with 1 μM of VP that was dissolved in DMSO (based on LeBlanc et al.^39^). After 6 days, we measured each population’s density (see ‘Cell counting’ subsection) and differentiation efficiency (see ‘Flow cytometry’ subsection). As a control, we examined the effect of adding only DMSO to cell culture medium without any VP. This ensured that our results were not due to any side effects of having DMSO that was carried over with the VP that we added to each medium. Results of these experiments are in Fig. 6c and Supplementary Fig. 28.

### Procedure for seeding a macroscopic colony (in Fig. 6f,g)

Our standard cell seeding method involves spreading a desired number of cells across the gelatin-coated surface of centimeter-sized dish. Unlike this method, we clustered a relative low number of cells (~5,000 46C cells per ml of N2B27) by injecting a few microliter droplets of N2B27 containing the amount of cells at the center of a centimeter-sized (for example, 10-cm or 6-cm) dish that was coated with 0.1% gelatin and contained the same volume of N2B27 as in the case of the standard cell seeding method (for example, 10 ml for 10-cm-diameter dish and 4 ml for 6-cm-diameter dish). The cells that were initially confined by a droplet were then allowed to sediment and attach to the gelatinized dish bottom, which, after 24 hours, resulted in an area of ~28 mm^2^, inside of which we observed localized, individual microcolonies that were not touching each other (imaged with bright-field microscopy; Fig. 6f). Over the next days, the microcolonies grew to touch each other to form a macroscopic colony that survived on its own.

### Reporting summary

Further information on research design is available in the Nature Portfolio Reporting Summary linked to this article.

## Online content

Any methods, additional references, Nature Portfolio reporting summaries, source data, extended data, supplementary information, acknowledgements, peer review information; details of author contributions and competing interests; and statements of data and code availability are available at 10.1038/s41589-022-01225-x.

## Supplementary information

Supplementary Information Supplementary Figs. 1–31, Supplementary Tables 1 and 2 and Supplementary Notes

Reporting Summary

Supplementary Data 1 Genes of interest in the RNA-seq dataset.

## Data Availability

Our RNA-seq data are available on the National Center for Biotechnology Information’s Gene Expression Omnibus (GEO) and are accessible through GEO series accession number GSE157642. As a reference genome for our RNA-seq analyses, we used the genome sequence of *Mus musculus* from UCSC (mm10), which is avilable here: https://genome.ucsc.edu/cgi-bin/hgGateway?db=mm10. All main data for this study are available at GitHub: https://github.com/youklab/Daneshpour-Stemcells-2022.

## Code availability

MATLAB codes used for data analysis, modeling and plotting are publicly available at GitHub: https://github.com/youklab/Daneshpour-Stemcells-2022.
