# Supplementary material for: Macroscopic quorum sensing sustains differentiating embryonic stem cells
Source: Nat Chem Biol. 2023 Jan 12;19(5):596–606. doi: 10.1038/s41589-022-01225-x (PMC10154202; doi:10.1038/s41589-022-01225-x)
Supplement: Supplementary file 1 — Supplementary Figs. 1–31, Supplementary Tables 1 and 2 and Supplementary Notes [file 41589_2022_1225_MOESM1_ESM.pdf]

nature chemical biology

Article

https://doi.org/10.1038/s41589-022-01225-x

Macroscopic quorum sensing sustains differentiating embryonic stem cells

In the format provided by the authors and unedited

Table of contents

- **Supplementary Figs. 1 - 31** .... **Pgs. 2-52**
- **Supplementary Tables 1 - 2** .... **Pgs. 53-56**
- **Supplementary Notes** .... **Pgs. 57-59**

A

B

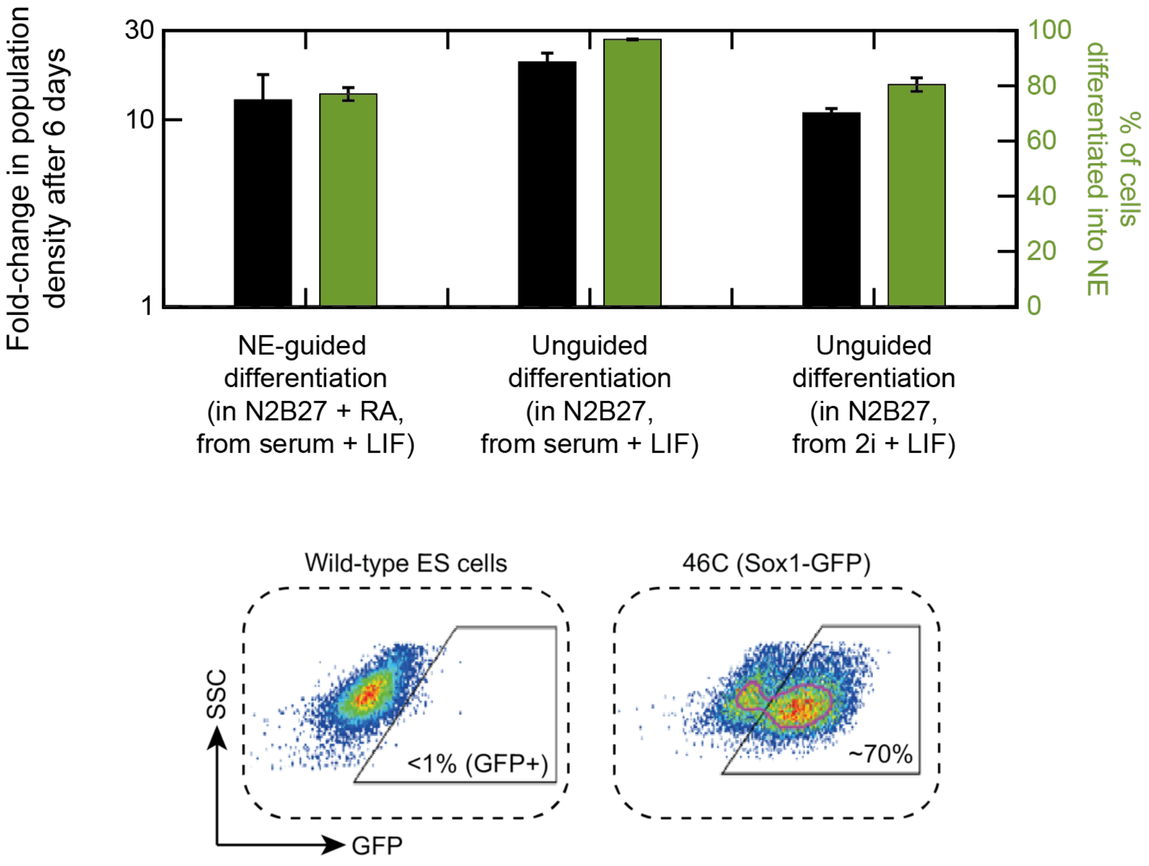

Supplementary Fig. 1 | Majority of ES cells (≥80%) differentiates into the Neural Ectoderm (NE) lineage without any inducers such as RA, regardless of which medium (2i or serum) the ES cells were self-renewing in prior to differentiating (related to Fig. 2). (A) Data shown for 46C cell line. The 46C cells have a Sox1 promoter controlling their GFP expression (Sox1 is a marker of the Neural Ectoderm (NE) lineage). Black bars represent fold-change in initial population density after 6 days of differentiation (measured with hematocytometer and confirmed with flow cytometer (see Supplementary Fig. 2)). Green bars represent percentage of cells becoming Sox1-GFP positive (measured with flow cytometer). For each differentiation, we took 46C cells that were kept pluripotent with serum+LIF or with 2i+LIF (i.e., serum-free) medium. These starting conditions are indicated in the captions below the black and green bars (e.g., “from serum + LIF”). The cells were then differentiated in N2B27 (without any serum), in the presence or absence of the inducer Retinoic Acid (RA) (added on day 2). These differentiation conditions are indicated in the captions below the black and green bars (e.g., “in N2B27 + RA”). As seen here, regardless of the use of inducers or which medium (2i or serum) the cells were self-renewing in prior to differentiating, all populations substantially expand (≥10 fold) and differentiate into the NE lineage (≥80%

becoming Sox1-GFP positive). These results agree with the findings in published papers[37,51]. *n* = 3; error bars are s.e.m. **(B)** Gating strategy used to measure percentage of 46C cells expressing Sox1-GFP. In the 2D plane defined by Side Scatter Channel (SSC) and FITC-A (GFP) channel, we drew a gate that included a negligible fraction of E14 ("widl-type") cells which do not have any GFP (left picture shows an example: less than 1% of the E14 cells are included in the gate). This gate includes 46C cells that express Sox1-GFP (right picture shows an example: ~70% of cells express Sox1-GFP in a population of 46C cells. Values for PMT voltages and wavelengths for measuring FSC, SSC, and fluorescence are in Methods.

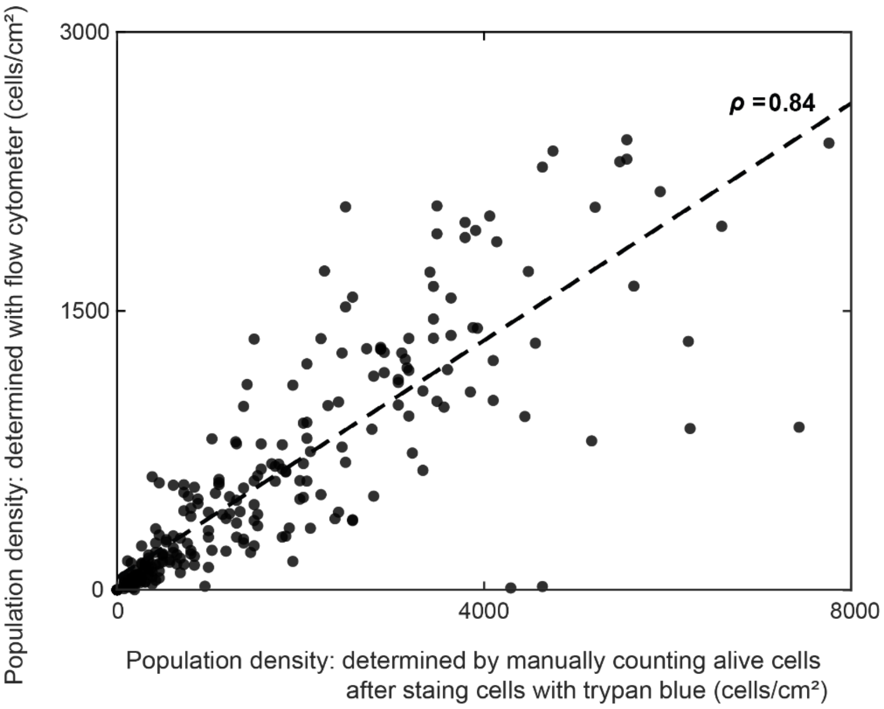

Supplementary Fig. 2 | Two different methods of counting cells yield population densities that are directly proportional to each other (i.e., offset by a constant factor of order one) and of the same order of magnitude, thus affirming both cell-counting methods for determining population densities (related to Fig. 2). Each data point represents one population whose density (# of cells/cm^2) was determined by two independent methods. Each axis represents a different method. As one method, we used a standard hemocytometer to count individual cells after subjecting the cells to the dye, trypan blue. Trypan blue penetrated only dead cells within the population. We counted the unstained (non-blue) cells with a hemocytometer to determine the resulting population density (# of cells/cm^2) of alive cells on a cell-culture dish. As another method to determine the population density, we used a flow cytometer to count the number of cells (events) that belonged to a specified FSC-SSC gate. We set the FSC-SSC gate so that it captured alive cells while excluding dead cells. These two methods yielded cell counts - and thus the corresponding population densities - that were directly proportional to one another, as indicated by a high Pearson linear correlation coefficient (black line; ρ = 0.84). The proportionality factor is on the order of one, meaning that, for the same population, the two methods yield numbers that are in the same order of magnitude as the population density. Given this result, throughout our study, we primarily counted cells manually (i.e., with trypan blue) and used the flow cytometer when this was not possible. Specifically, we used the flow cytometer to count populations that had very

few cells such as those near extinction (i.e., populations whose fold-change in density was near or below 0.1 after some days).

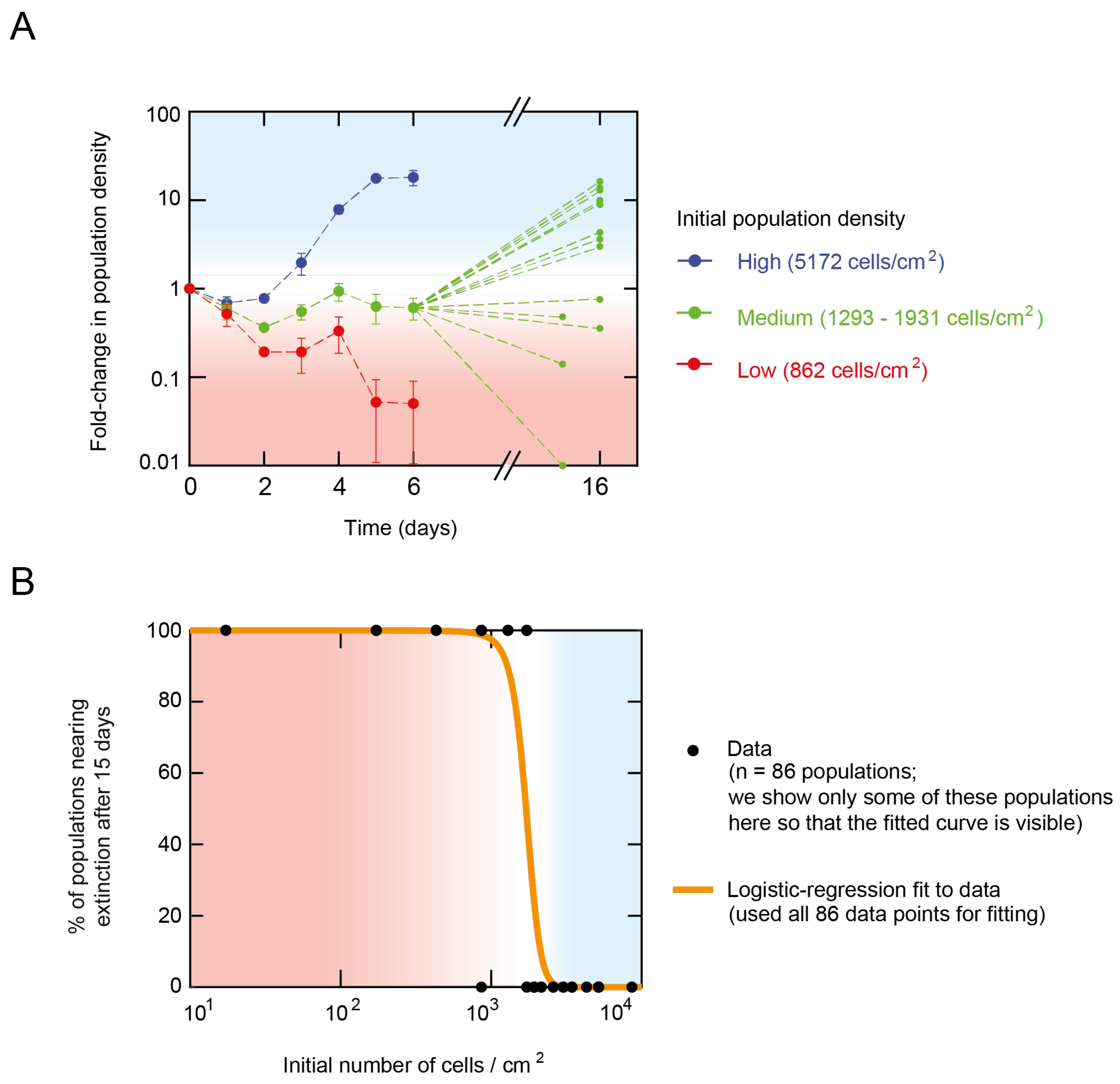

Supplementary Fig. 3 | Differentiating populations that start with nearly a threshold population-density (~1700 cells/cm^2) neither expand nor shrink during the first six days. But, after more time passes, these populations either expand towards the carrying capacity or shrink towards extinction in a stochastic manner (i.e., two populations of the same starting density can have two different fates: one survives whereas the other one heads towards extinction) (related to Figs. 2 and 3). Data for 46C cells differentiating towards NE lineage in N2B27 + Retinoic Acid (RA) that were self-renewing in serum+LIF prior to differentiating (Methods). **(A)** Blue: populations that started with a sufficiently high density (5172 cells/cm^2) all grew towards the carrying capacity. Red: populations that started with a sufficiently low density (862 cells/cm^2) headed towards extinction during the first 6 days. Green: populations that started near a threshold population-density (between 1293 and 1931 cells/cm^2) neither grew nor shrank in the first 6 days. However, by 15-16 days after removing

LIF (i.e., beginning differentiation), some of these populations reached the carrying capacity (i.e., population density increased by ~10-fold) while some others became extinct (i.e., population density became ~0.1-fold or less). Still, some populations maintained nearly the same density for these 15-16 days (i.e., green curves with fold change of nearly one after 15-16 days). Thus, populations having virtually the same initial density can have distinct fates: some would become extinct whereas some would survive (i.e., population-level growth is stochastically (randomly) determined). For all the data shown for the first six days, *n* = 3 and error bars are s.e.m. Each green data point, taken 15-16 days after starting differentiation, represents a single population. **(B)** To quantify the stochastic nature of the population growth for populations that start with a near-threshold density (green data in (A)), we measured the fold-change in population density for a total of 86 populations that collectively spanned a wide range of initial densities (black points). To each population, we either assigned a value of zero if it eventually grew towards the carrying capacity (i.e., fold-change of larger than 1) or a value of one if it eventually approached extinction within the first 16 days of differentiation. Then, we performed a logistic regression on these black data points by fitting a logistic function, $p(x) = \frac{1}{1+e^{-(\beta_0+\beta_1 x)}}$ (orange curve). Consequently, $p(x)$ represents the probability that a population approaches extinction by 15-16 days after differentiation began. By fitting, we found $\beta_1 = -8.7856 \pm 0.8780$ with a p-value of 3.76×10^{-25} according to the Wald test. This logistic regression is the simplest model (null model) that we can have for describing the probability of becoming extinct without any information about the mechanisms that determine the survival-versus-extinction fate. Our stochastic model recapitulates this logistic regression fit (in Fig. 3f).

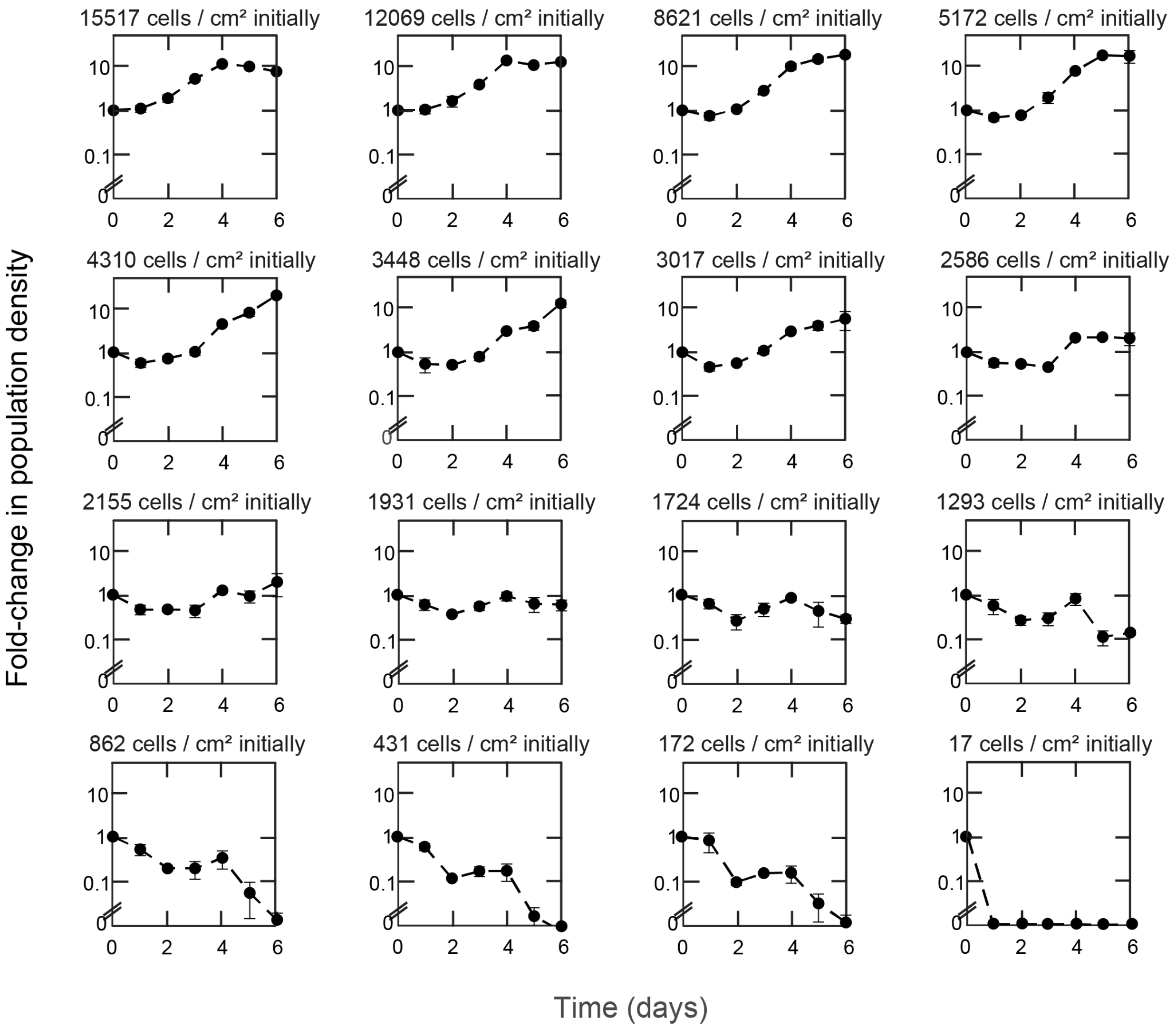

Supplementary Fig. 4 | Fold-change in population density during differentiation towards the Neural Ectoderm (NE) lineage for a wide range of initial population-densities (related to Fig. 2). Data for 46C cells differentiating towards NE lineage in N2B27+ Retinoic Acid (RA) that were self-renewing in serum+LIF prior to differentiating (Methods). We triggered pluripotency exit to begin each time course shown in each box (protocol in Fig. 2a). Each box shows the population-growth dynamics for one initial population-density (indicated above each box). Each box shows the fold-change in population density (vertical axis) as a function of the time passed since starting the differentiation (horizontal axis). *n* is at least 3 for each data point. Error bars are s.e.m.

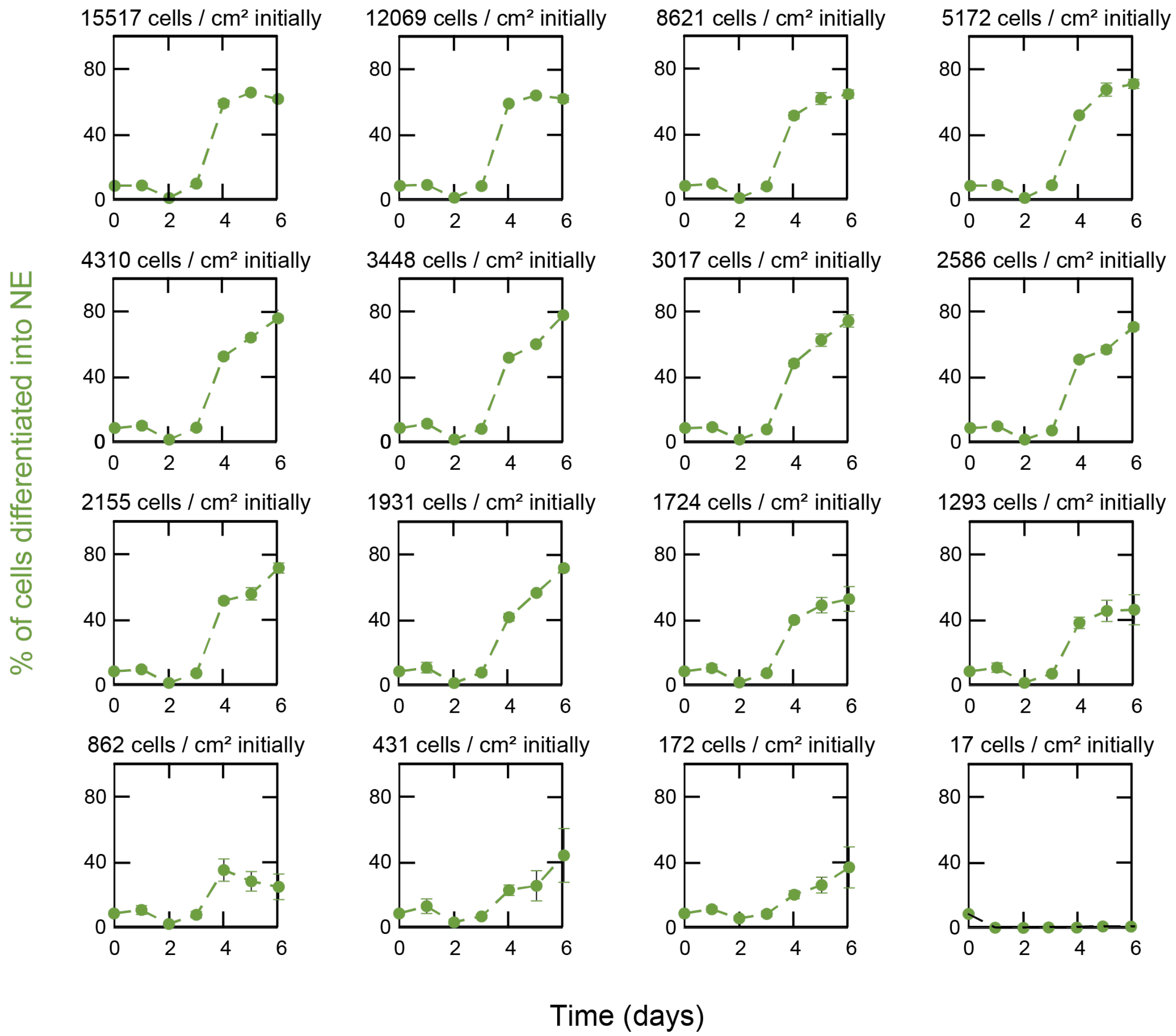

Supplementary Fig. 5 | Populations with higher initial densities have larger percentages of cells that differentiate into the Neural Ectodermal (NE) lineage) (related to Fig. 2). Same data and procedure as described in Supplementary Fig. 4. Each box shows the differentiation efficiency for a specific initial population-density (indicated above each box). On each day, we detached all cells from a 10-cm-diameter dish and then flowed them through a flow cytometer to measure the percentage of alive cells in the population that expressed GFP (i.e., percentage of alive cells that expressed Sox1, a marker of NE-lineage entrance) (Methods). Differentiation efficiency reached a maximum of ~80% for populations that started above the threshold population-density (~1700 cells/cm^2). Differentiation efficiency was below ~50% for populations that began with a below-threshold density. *n* is at least 3; error bars are s.e.m.

A
B

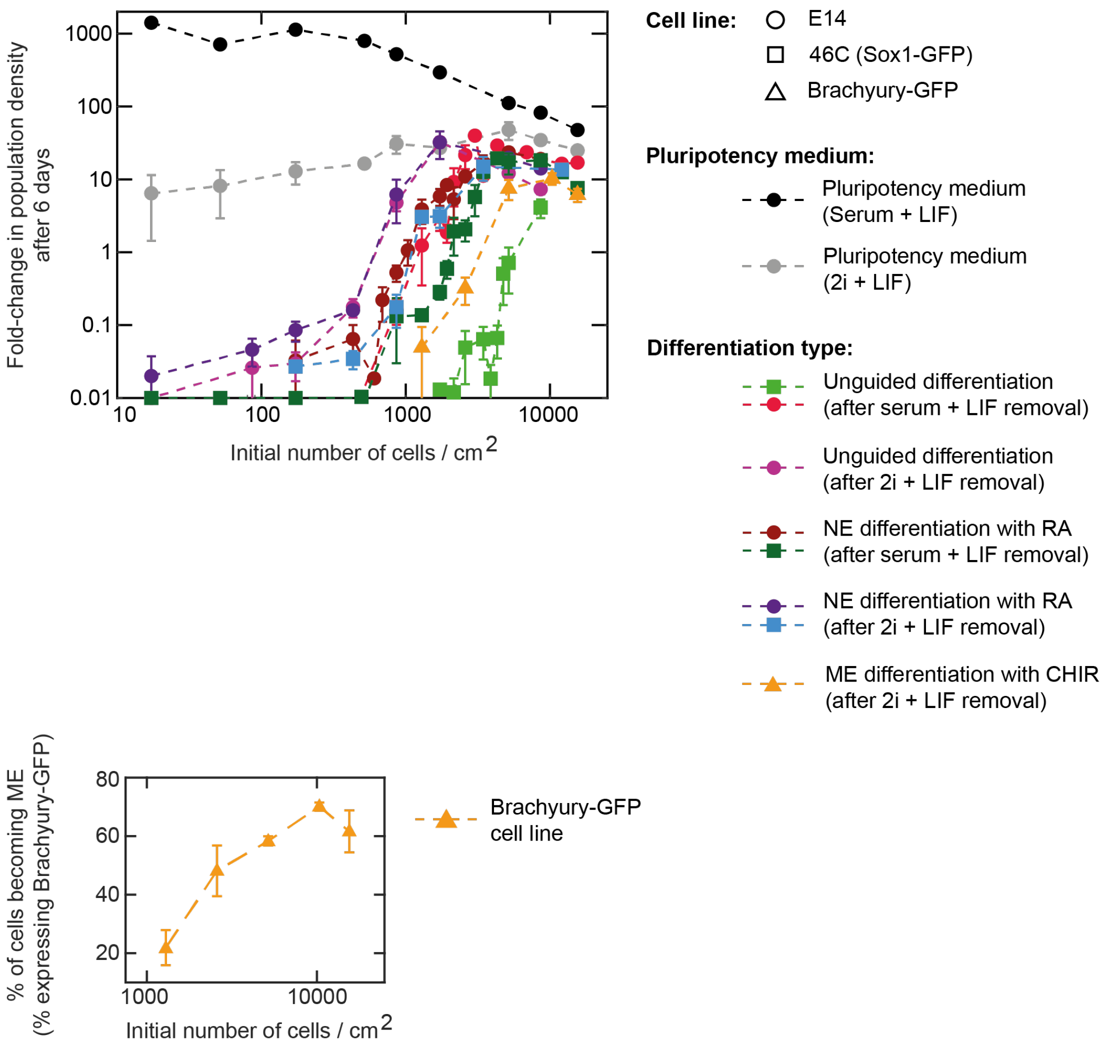

Supplementary Fig. 6 | Three distinct cell-lines and multiple ways of differentiating all exhibit the same phenomenon: collective growth with a threshold population-density (related to Fig. 2). (A) Data for three different cell-lines: E14 (circles), 46C (squares), and Brachyury-GFP (triangles). See Methods for full details on these cell-lines. The 46C cells have a Sox1 promoter controlling their GFP expression (Sox1 is a marker of the Neural Ectoderm (NE) lineage). The Brachyury-GFP cells have a Brachyury promoter controlling their GFP expression[38]. Brachyury is a marker of Mesendoderm (ME) lineage. Different colors represent different types of cell-culture media as indicated in the legend. For each differentiation, we took one of the three cell lines that were self-renewing with LIF in either a serum-based medium or a serum-free (2i) medium. The three types of differentiations are: unguided differentiation in which no inducer was added to N2B27, NE differentiation in which we added Retinoic Acid (RA) to N2B27, and ME differentiation in which we added the small molecule,

CHIR, to N2B27 (Methods). For the latter two, inducers were added to N2B27 only two days of differentiating in N2B27 without any inducers. As seen here, regardless of the cell line and differentiation type, a differentiating population's initial density determined whether the population grows or approaches extinction. **(B)** After four days of CHIR-induced differentiation, we used a flow cytometer to measure the percentage of the Brachyury-GFP cells that expressed GFP (i.e., percentage of cells in a population that entered the ME lineage[35]). Prior to differentiating, the cells were self-renewing in 2i+LIF. As with the 46C cells that differentiated towards the NE lineage (Fig. 2c), populations of Brachyury-GFP cells that started with higher densities had higher differentiation efficiencies (larger percentages of cells entering the ME lineage). For both (A) and (B): n = 3; error bars are s.e.m.

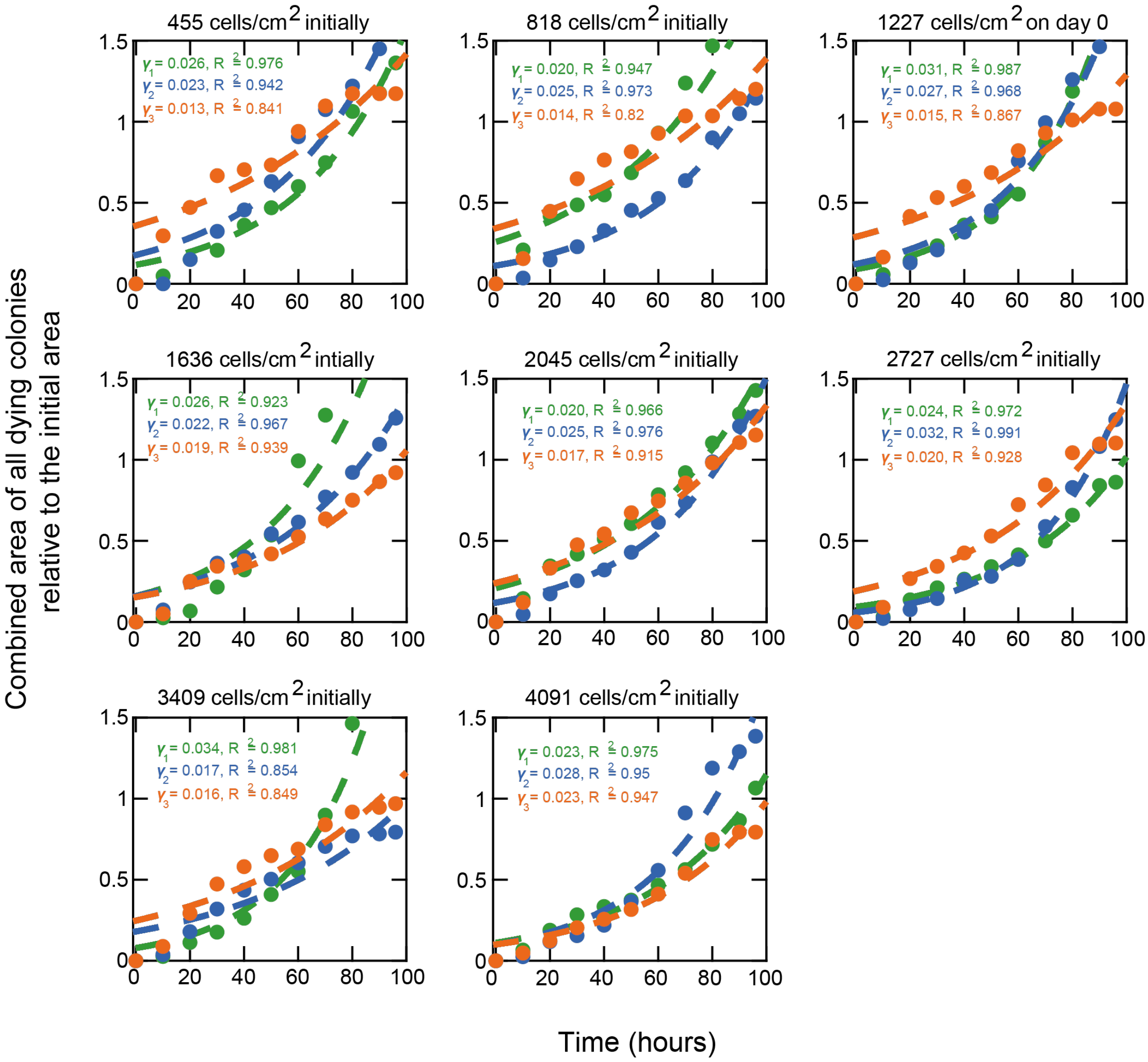

Supplementary Fig. 7 | Determining the rate of cell death with time-lapse microscopy (related to Fig. 3). We sought to verify that the rate of cell death (death rate) is independent of the initial population density. This is an ingredient of our stochastic model (Methods). With time-lapse microscopy, we measured the growth of individual microcolonies over four days for a wide range of initial population-densities. Data shown for E14 cells in unguided differentiation in N2B27 (without any inducers such as RA) that were previously self-renewing in serum+LIF (Methods). Each box shows a population of a different starting density (indicated above each box). For each replicate of an initial population-density (green, blue, and orange curves), we tracked microcolonies in 17 fields of view on a 6-cm diameter dish (one dish per replicate population). Microscope's field-of-view has a dimension of 1.40 mm x 0.99 mm. Dying colonies typically lifted off the plate (and thus disappeared from the field of view) or started to display clearly visible apoptotic bodies. From these movies, we inferred the death rate of cells by taking the cumulative sum of the last recorded areas of each colony just before it died, to measure how much colony area is "lost" (i.e., deaths) in time. We then divided this combined

area of all dead cells by the combined initial area (thus the vertical axis is reported in dimensionless numbers in each box). We determined the death rate by fitting a single exponential function, with the death rate γ (in 1/hours), to the data points (dashed curves represent the fits to data points of corresponding color; each color is a single replicate; n = 3 for each initial density) (see Supplementary Fig. 8A for a demonstration of this fitting). The values of γ and corresponding R^2 are indicated in each plot. Error bars are s.e.m. This figure complements Supplementary Fig. 8.

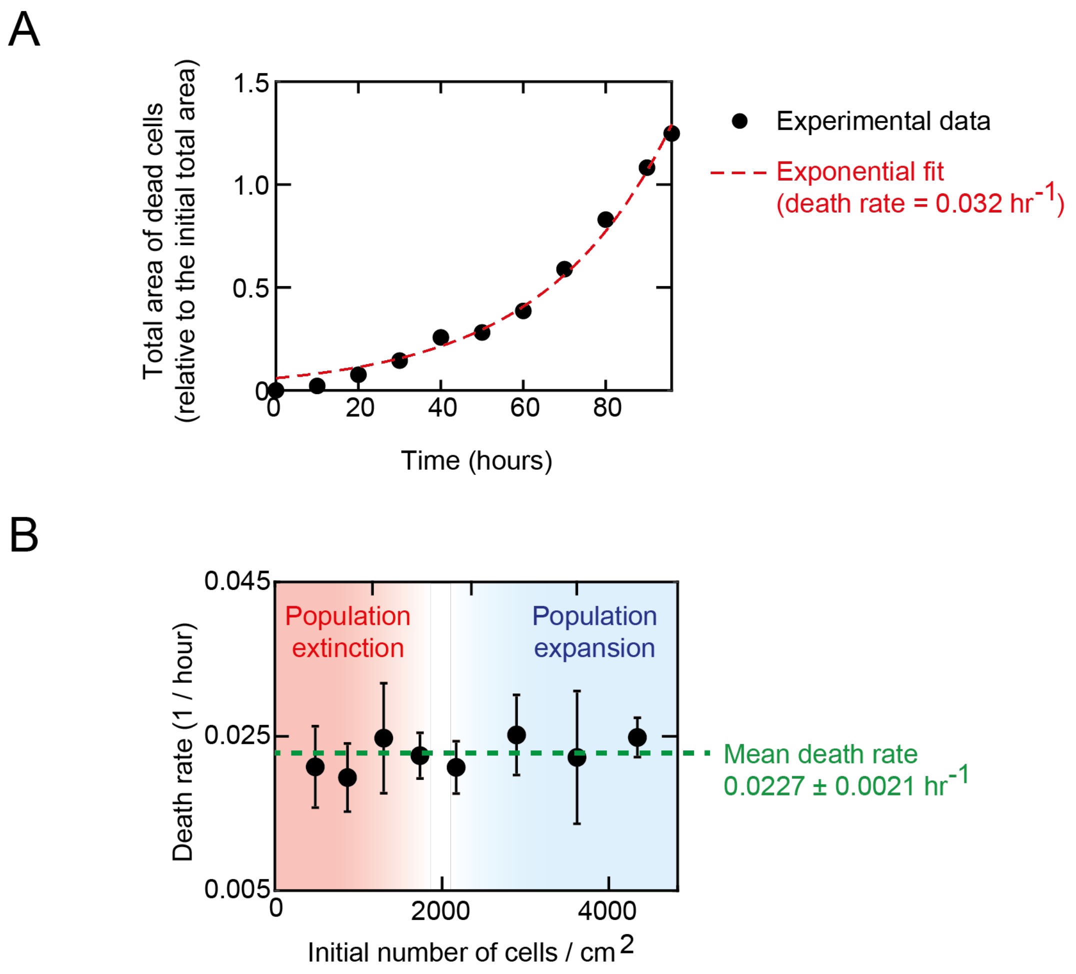

Supplementary Fig. 8 | Rate of cell death (death rate) is independent of the initial population-density (related to Fig. 3). Data for E14 cells in unguided differentiation in N2B27 that were previously self-renewing in serum+LIF (Methods). **(A)** Example that shows how we inferred the death rate for one population from measuring and summing up the area of dead cells at each timeframe of microscope time-lapse movie. See explanation in the caption of Supplementary Fig. 7. **(B)** Death rate extracted in Supplementary Fig. 7 (as in (A)) for various initial population-densities. This plot shows that the death rate of differentiating cells (0.0227 ± 0.0021 hr^{-1}) is independent of the initial population-density. n = 3; Error bars are s.e.m.

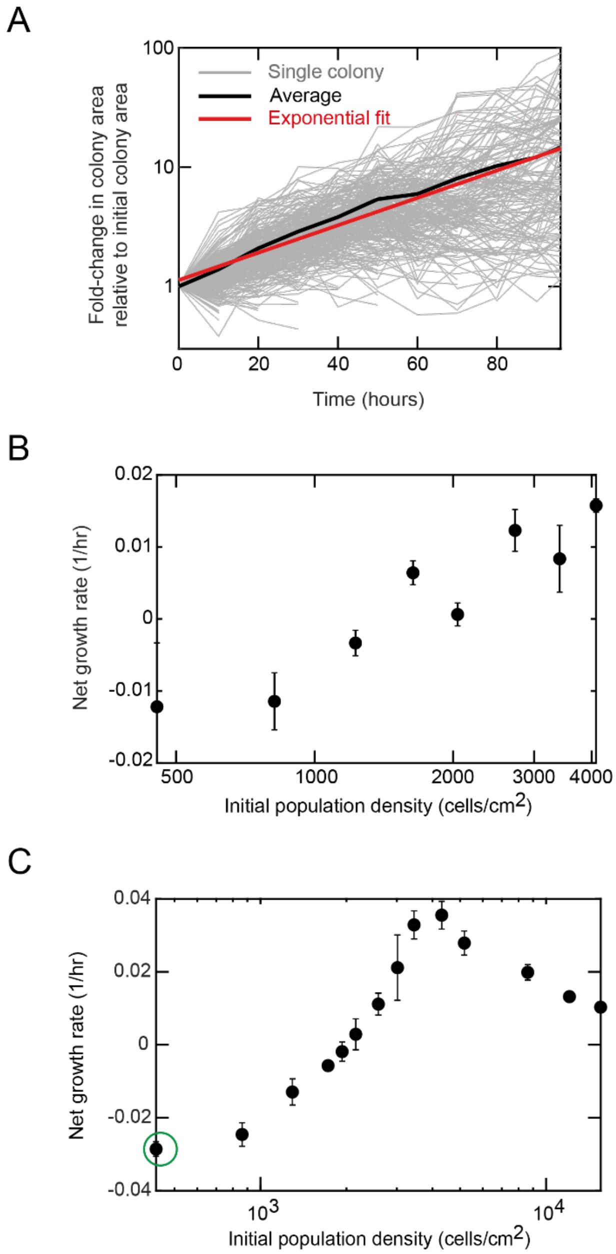

Supplementary Fig. 9 | Net growth rate (growth rate - death rate), measured in two ways, depends on population density (related to Fig. 3). Data for E14 cells in unguided differentiation in N2B27. These cells were self-renewing in serum+LIF prior to differentiation (Methods). Net growth rate is negative if the death rate is higher than the growth rate. "Growth rate" is the cell-replication rate (number of cells born per unit time). Throughout our paper, a population with a negative net growth rate is defined to be approaching extinction (this is our definition of "population extinction"). The net growth rate is positive if the growth rate is larger than the death rate. Throughout our paper, a population with a positive net growth rate is defined to be growing towards the carrying capacity (this is our definition of "population grows" and "population survives"). We measured the cells' net growth rates in two different ways. In one, we used time-lapse microscopy data to measure colony growths. In the other, we

manually counted cells (and thus the population density) over time as in Fig. 2c. **(A)** Example showing the microscope-based method of determining the net growth rate for a given initial population density. As shown in Supplementary Fig. 10, we continuously measured the area of each colony for four days. We imaged 17 fields-of-view for each replicate of an initial population-density. Each grey curve shows the area of a single microcolony over time. Each colony's area is normalized to its initial area (thus all curves here start at a value of one on the vertical axis). Black curve is the average of all the grey curves. Red line is an exponential curve (line in this semi-log plot) that we fitted to the black curve (i.e., fitted to the population average). The slope of the red line is the net growth rate for this population. **(B)** Using the microscope-based method outlined in (A) for every initial population-density, we obtained the growth rate (black dots) as a function of the initial population density. n = 3; Error bars are s.e.m. **(C)** Net growth rates determined by manual counting of cells (i.e., data in Supplementary Fig. 4) rather than from the microscopy data. n = 3; Error bars are s.e.m.

To determine the (non-net) growth rate for each initial population density, we added the constant death rate (determined in Supplementary Fig. 8) to the net growth rate (determined in (B-C)). We found that the maximum possible growth rate was 0.05219 hr^{-1}, which we used as one of the parameter values in our mathematical model. From (C), we found that the lowest possible net growth rate was -0.026 hr^{-1} (indicated with green arrow in (C)). This value nearly matches the death rate (0.023 ± 0.002 hr^{-1}; found in Supplementary Fig. 8B). Our stochastic model uses these values for the growth and death rates (Methods).

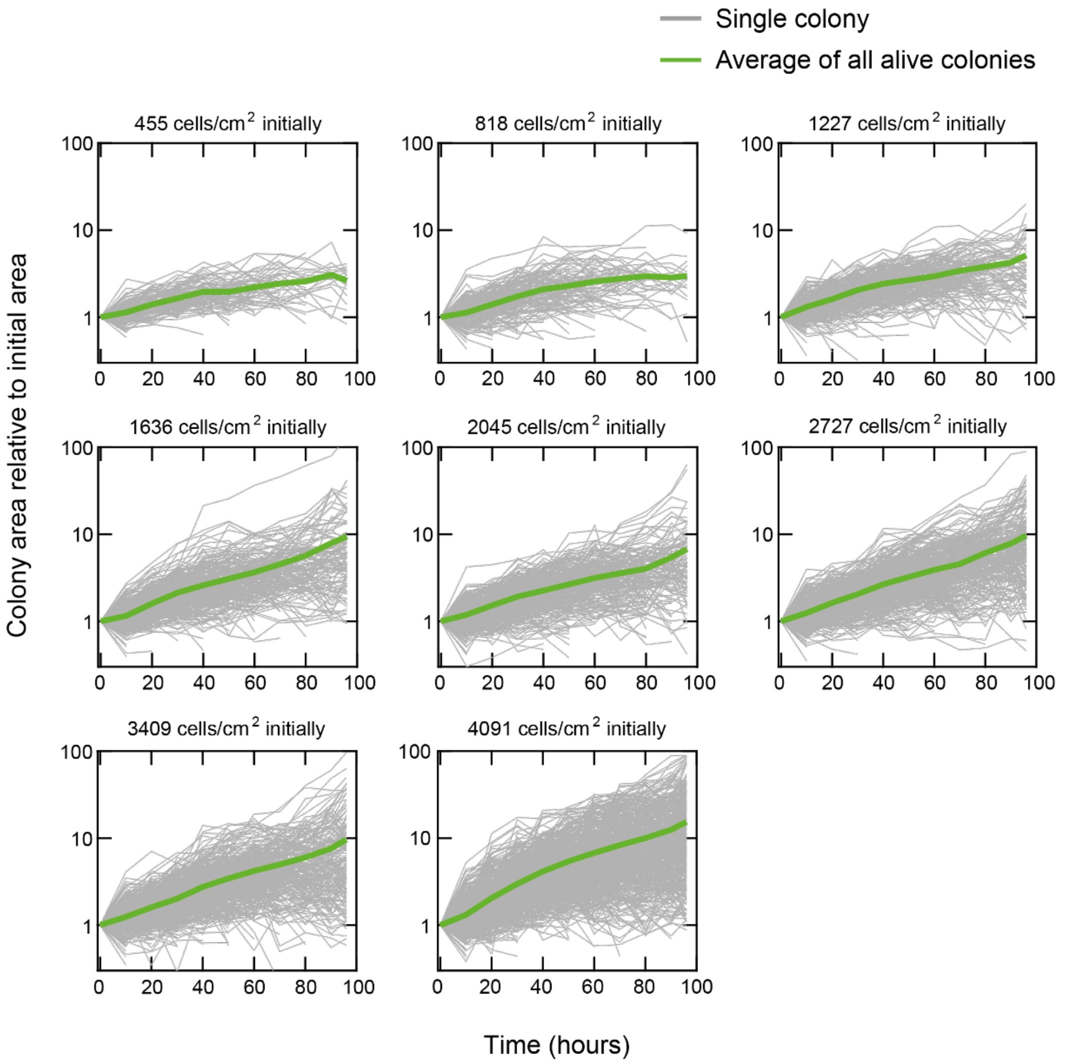

Supplementary Fig. 10 | Time-lapse microscopy over four days reveals the growth rate of each microcolony for a wide range of initial population-densities (related to Fig. 3). Data for E14 cells in unguided differentiation in N2B27 that were self-renewing in serum+LIF prior to differentiating (Methods). We used a wide-field microscope to continuously monitor and measure the area of each microcolony over four days (each grey curve). During the four days, we measured the area of a given microcolony every 10 hours (Methods). Each box shows the fold-change in the colony area (vertical axis) as a function of the time passed since triggering differentiation (horizontal axis) for different starting population-densities. In these movies, we observed both growing and dying colonies. For the colonies that died, the grey curves end at the last time frame in which they were alive. The dying colonies visibly stood out as they typically displayed apoptotic bodies or lifted off the plate and thus disappeared from the focal plane. At each time frame, we computed the average area of all living colonies (green curve) from which we can extract the average growth rate of a colony for each population density.

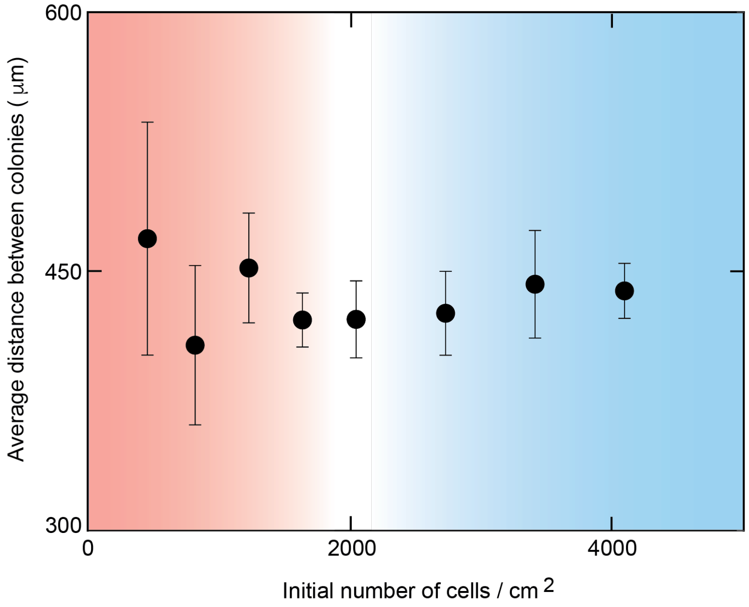

Supplementary Fig. 11 | For every population density, microcolonies are hundreds of microns apart from each other when differentiation begins (related to Figs. 2 and 4). Data for E14 cells in unguided differentiation in N2B27 that were self-renewing in serum+LIF prior to differentiating (Methods). We scattered a desired number of cells across a 6-cm diameter dish containing N2B27 to trigger differentiation. Then, we used a wide-field microscope to locate and image microcolonies in 17 fields-of-view per dish. A microscope's field-of-view had a dimension of 1.40 mm x 0.99 mm. From these images, we determined the distance between every pair of microcolonies that resided in the same field-of-view. Then, we averaged these distances (averaging over all pairs of colonies from all 17 fields-of-view per dish). The resulting, average distance between microcolonies is plotted here as a function of the initial population density. As shown here, for a wide range of initial population-densities, the average distance between microcolonies were virtually identical (~450 μm). n = 3 dishes for each initial population-density; Error bars are s.e.m.

A way to understand why the average colony-colony distance is nearly independent of the initial population-density is that the dish area is much larger than can be covered by the cells, even for the very high-density populations (at most ~1% of dish is covered by the cells, as one can also check by a back-of-envelope calculation with an estimate of one cell's area). In short, the vast difference in length-scale between each cell's size and the dish size leads to above result. Given this, a more informative metric is the distance between a colony and its nearest-neighboring colony (see Fig. 2b and Supplementary Fig. 14).

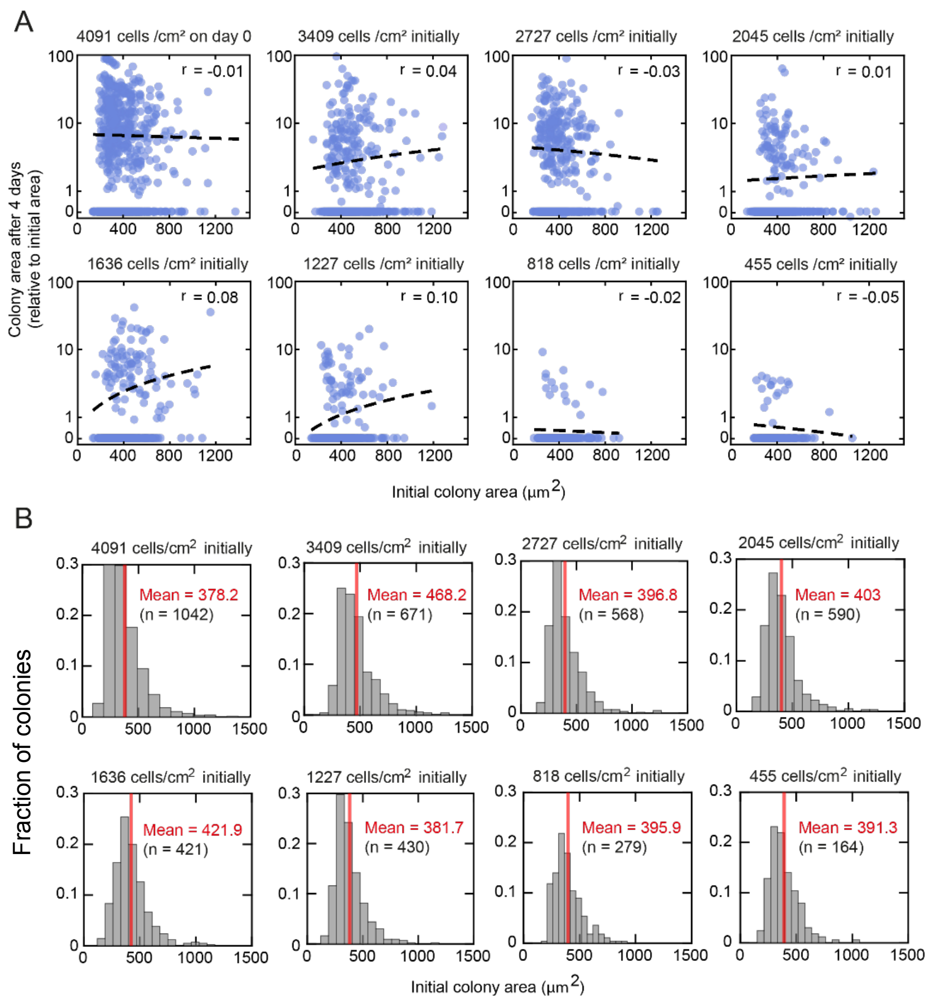

Supplementary Fig. 12 | Microcolony's survival and growth rate do not depend on how many cells are initially in the colony for any population density (related to Fig. 4). (A, B) Data for E14 cells in unguided differentiation in N2B27 that were self-renewing in serum+LIF prior to differentiating (Methods). Same protocol as in Supplementary Fig. 10. (**A)** We used a microscope to continuously monitor and measure the colony area during four days of differentiation. Each blue point represents the final area of a colony (after 96 hours) relative to its initial area. If a colony died at some timeframe in the time-lapse movie, then we assigned it a value of zero as the fold-change of its area (as explained in caption for Supplementary Fig. 10). Each box shows the fold-change in the colony area (vertical axis) as a function of the initial colony area (horizontal axis) for 17 fields-of-view and for a specific population density. Dashed line in each box is the linear regression (Pearson correlation coefficient *r* in each box). There is virtually no correlation between a colony's final area (and whether it dies or not) and its initial area, for any starting population density. Thus, the initial area of a microcolony, set by the number of cells in a microcolony, does not determine a colony's growth rate. **(B)** Histograms of the colony-area, at the start of the time-lapse movies in (A), for each initial population-density.

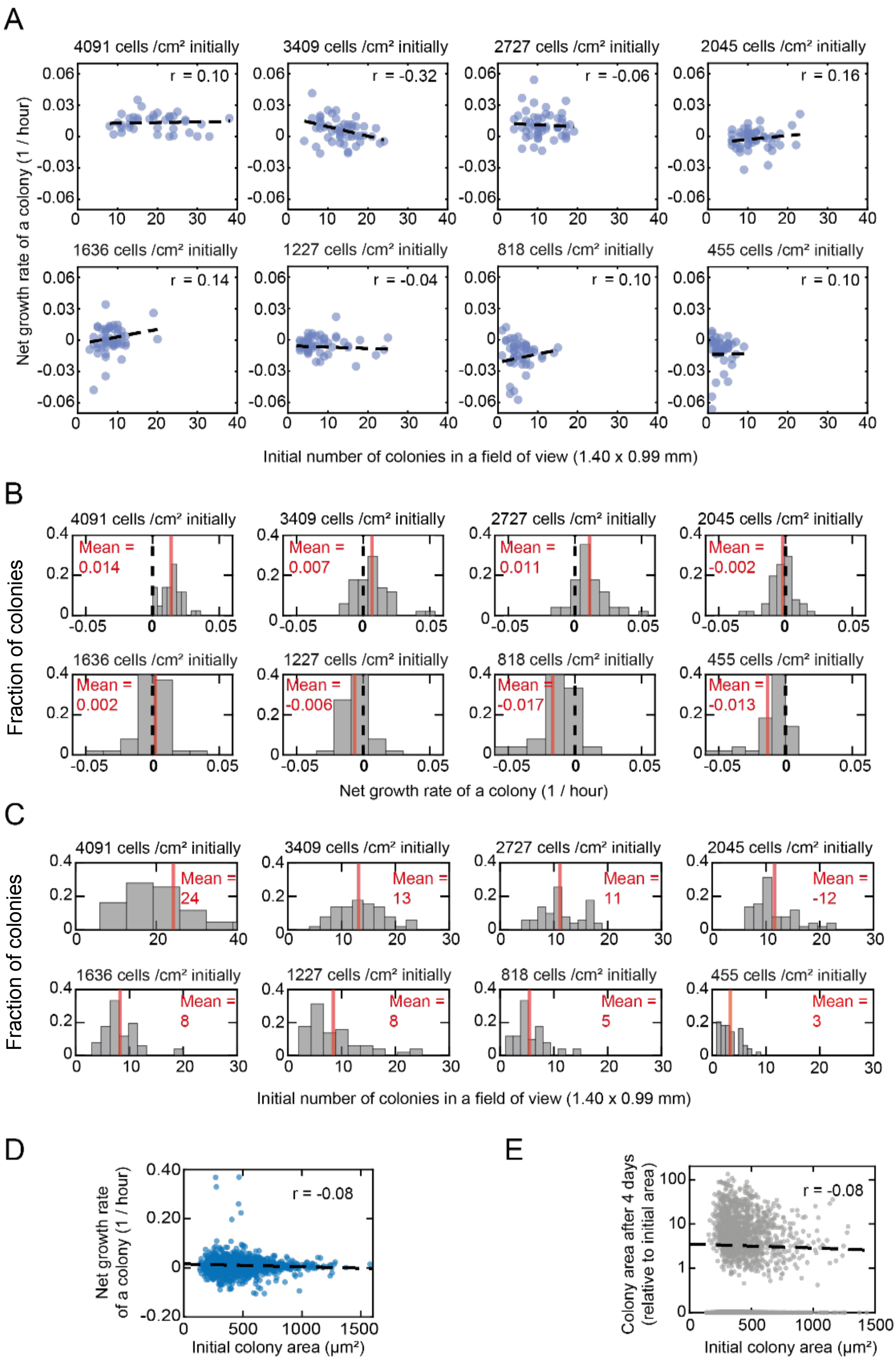

Supplementary Fig. 13 | Net growth rate of a microcolony does not depend on how many cells are in the microcolony (colony area) for any population-density (related to

Fig. 4). Data for E14 cells in unguided differentiation in N2B27 that were self-renewing in serum+LIF prior to the differentiation (Methods). Same protocol as in Supplementary Fig. 10. **(A-C)** We fitted an exponential function, Area(t) = $A_0\exp(\mu t)$, to each grey trace shown in Supplementary Fig. 10. From this fit, we determined net growth rate μ for every microcolony. For each microcolony, we determined its net growth rate and the total number of microcolonies that initially resided in the same field-of-view. In (A), each blue point represents one microcolony. The net growth rate is positive ($\mu > 0$) if a microcolony grew over the four days of differentiation whereas it is negative ($\mu < 0$) if the microcolony died, detached from the plate, and thereby disappeared from the field-of-view. Each box shows a colony's net growth rate (vertical axis) as a function of the number of colonies that existed, at the start of the time-lapse movie, in the same field-of-view (horizontal axis). We analyzed 17 fields-of-view for each initial population-density. Dashed lines in each box shows the Pearson correlation with the correlation coefficient *r* as indicated. (B) shows histograms of the net growth rate of a microcolony. (C) shows histograms of the number of microcolonies that each field-of-view began with. Data in (A-C) establish that a colony's grow rate - whether it survives or dies - is virtually uncorrelated with how many other microcolonies exist in its ~1 mm x 1 mm vicinity. **(D)** There is virtually no correlation between a microcolony's net growth rate and its initial area, as shown here. Data points come from many different, initial population-densities and are pooled together here. Dashed line shows the Pearson correlation with a correlation coefficient $r = -0.08$. **(E)** There is virtually no correlation between a microcolony's final area relative to its initial area and its initial area, as shown here. Data points come from many different, initial population-densities and are pooled together here. Dashed line shows the Pearson correlation with a correlation coefficient $r = -0.08$.

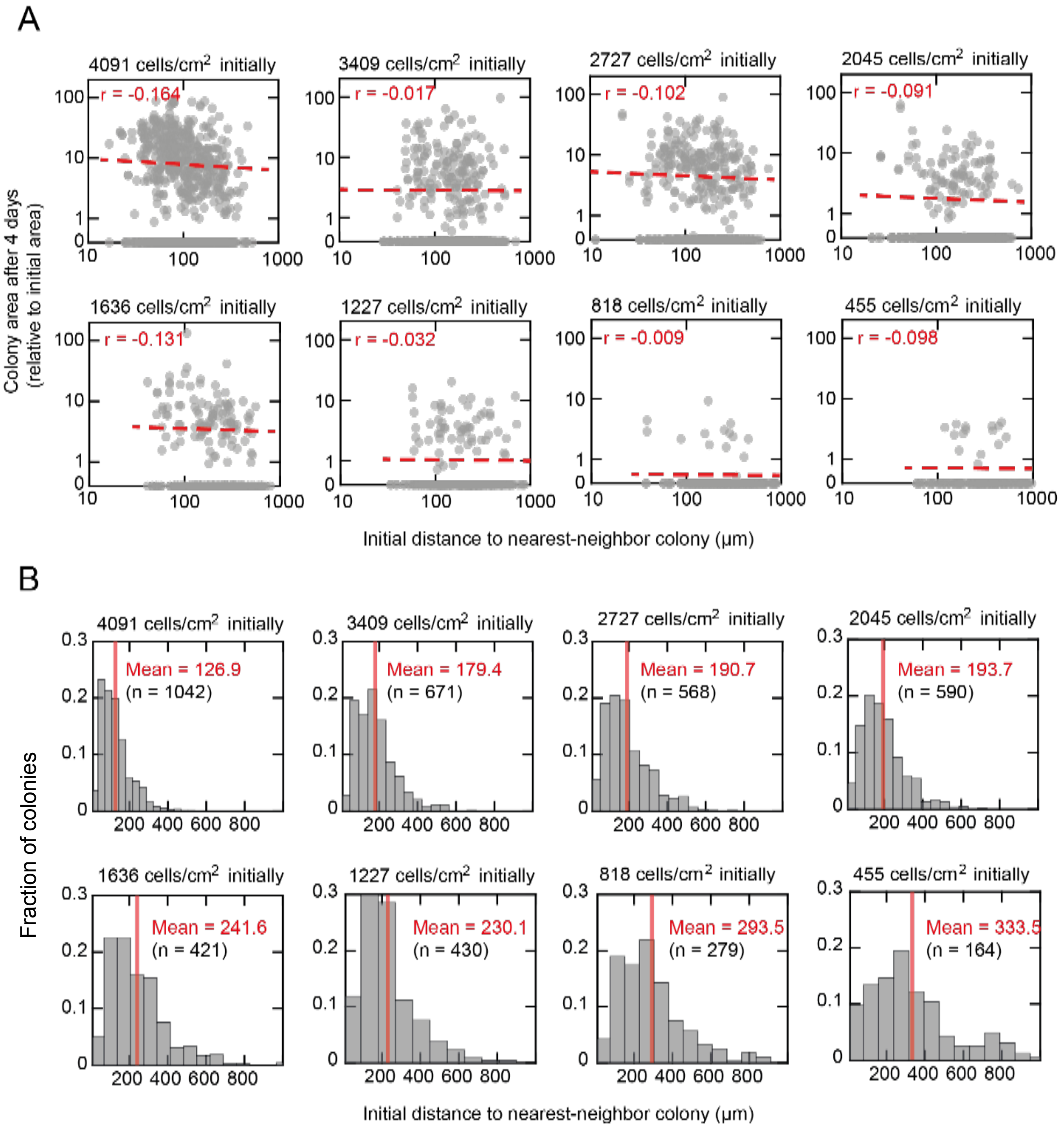

Supplementary Fig. 14 | Net growth rate of a microcolony cells does not depend on the distance between nearest-neighboring colonies for any initial population-density (related to Fig. 4). (A-B) Data for E14 cells in unguided differentiation in N2B27 that were self-renewing in serum+LIF prior to differentiating (Methods). Same protocol as in Supplementary Fig. 10. We scattered a desired number of cells across a 6-cm diameter dish containing N2B27 to initiate differentiation. Then, we used a wide-field microscope to continuously monitor microcolonies in 17 fields-of-view (Methods). Each field-of-view has a dimension of 1.40 mm x 0.99 mm. From the resulting time-lapse movies, we determined the distance between every pair of colonies that resided in the same field-of-view. From these distances, we found the smallest distance (i.e., distance from one colony to its nearest-

neighboring-colony). We did this for each colony in every field-of-view. The resulting, nearest-neighboring distance for each colony is plotted here as a function of the initial population-density. Each grey point represents the final area (after 96 hours) of a colony relative to its initial area as a function its distance to its nearest neighbor (at the start of the time-lapse movie). If a colony died during the time-lapse, then we assigned it a value of zero as the fold-change in its area. Each box shows the fold-change in the colony area (vertical axis) as a function of the distance from colony to its nearest neighbor (horizontal axis) for 17 fields of view and a specific population density. Dashed curves in each box denote the Pearson correlation with the correlation coefficient *r* in each box. There is virtually no correlation between a colony's final area (and whether it dies or not) and its initial, nearest-neighbor distance, for any starting population density. These results strongly indicate that the presence of a nearby colony does not predict whether a colony survives or not and does not predict how fast each colony grows during differentiation.

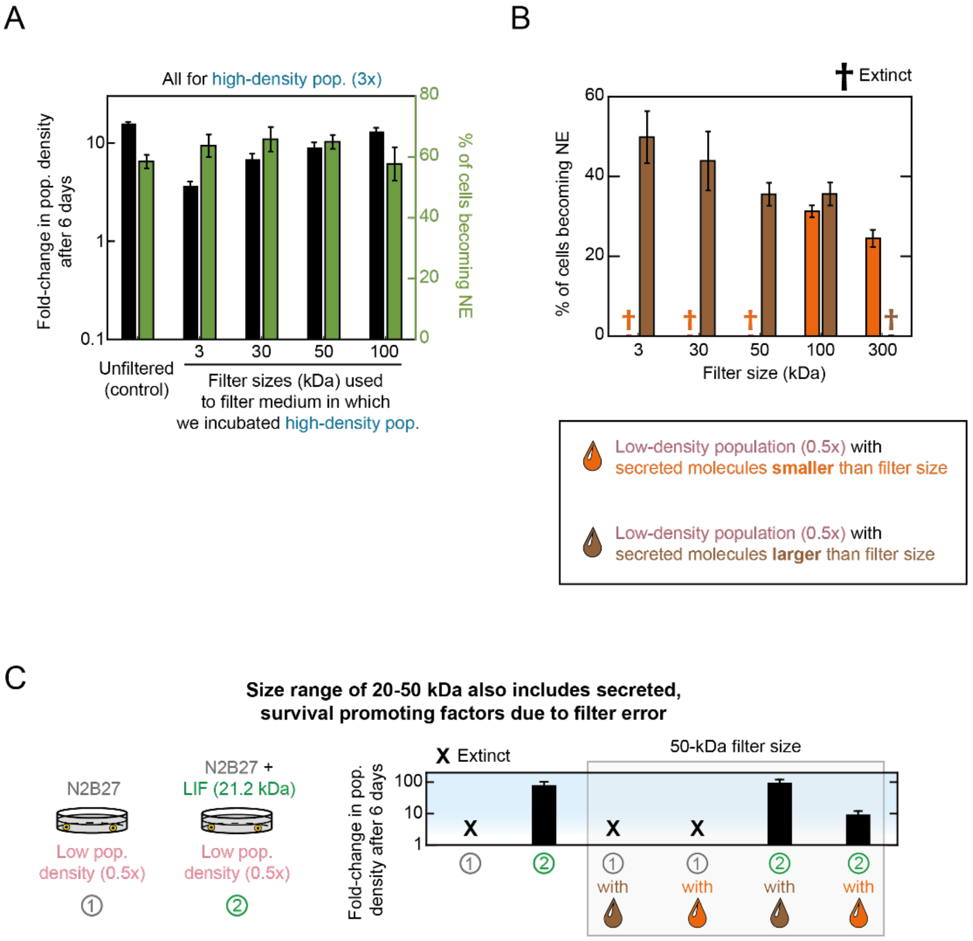

Supplementary Fig. 15 | Filtering culture medium does not eliminate media components that are essential for cells to grow (related to Extended Data Fig. 1). We used commercial, membrane-based filters of various sizes (Methods and Extended Data Fig. 1). Data in (A) and (B) for 46C cells differentiating in N2B27 + RA that were self-renewing in serum+LIF prior to differentiating. **(A)** To check that the filters do not remove any media components that are essential for the cells to grow, we filtered the medium of a high-density population (5172 cells/cm^2), after two days of differentiation, with filters of various sizes. We then took the filtered medium (medium that passed through the filter and thus containing all molecules that are smaller than the filter size) and gave it back to the same high-density population. We incubated the population for four days in this medium. On the last day, we measured the fold-change in population density (black bars) and the percentage of cells becoming NE (green bars). As a control, we also measured the fold-change in population density after six days of growth in the unfiltered medium (first black and green bars). Since all black bars have nearly the same height as do all the green bars, we can conclude that none of the filters capture any ingredients in the cell-culture medium that are essential for cell growth (e.g., vitamins and other components which are already present in the medium from the beginning of cell culture, rather

than secreted by cells). **(B)** We cultured a low-density population (862 cells/cm^2) in a medium from a high-density population (5172 cells/cm^2) that went through the filter (orange bars). This medium has all molecules that are smaller than the filter size. We also cultured the low-density population in a medium that contained all the molecules that are larger (heavier) than the filter size (brown bars), which we captured by flowing the high-density population's medium through the filter. We used a wide range of filter sizes (horizontal axis). The bars show the percentages of cells entering the NE after being cultured in the filtered medium. Crosses indicate that the population became extinct in the filtered medium. The corresponding fold-changes in population density are shown in Extended Data Fig. 1b. **(C)** To check the accuracy of the filters in capturing a molecule of known size, we filtered N2B27 with a 50-kDa size filter (we show in (B) that this procedure does not catch any ingredients in N2B27 that are essential for cell growth) that was supplemented with recombinant LIF (1000 U/mL). We then measured the fold-change in population density (black bars). Same color scheme is used as shown in (B). Recombinant LIF has a molecular weight of ~20 kDa and we confirmed that unfiltered N2B27+LIF results in a ~100-fold expansion of a low-density population (46C cells, previously self-renewing in serum+LIF) that otherwise becomes extinct during differentiation (here, N2B27 without any inducer such as RA). Both fractions of filtered N2B27+LIF resulted in the rescue and expansion of the low-density population, but the fraction containing molecules larger (heavier) than 50 kDa resulted in the largest possible expansion which was virtually identical to the expansion achieved with unfiltered N2B27+LIF.

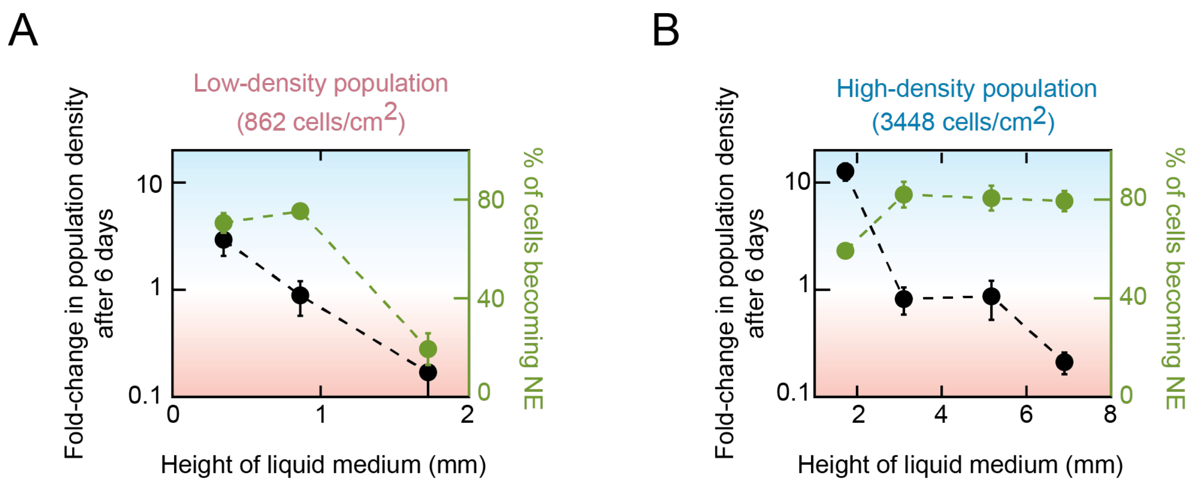

Supplementary Fig. 16 | Changing the height of the cell-culture medium by millimeters alters the population's growth (related to Fig. 5). Data for 46C cells differentiating in N2B27 with RA that were self-renewing in serum+LIF prior to differentiating. Black data points are duplicates of the data in Fig. 5b. They indicate the fold-change in the population density, relative to the initial population-density, as a function of the height of the liquid-medium. Green data points indicate the percentage of the cells that expressed GFP (measured with a flow cytometer) as a function of the liquid height. In our study, we used a 10-mL of growth medium (e.g., in Fig. 2) unless we explicitly state that we used a different volume (e.g., in Fig. 5b and here). A 10-mL liquid has a height that is just below 2 mm in a 10-cm diameter dish. **(A)** Data for a differentiating population that starts with a low density (862 cells / cm^2). In a 10-mL liquid, this population becomes extinct (last data point, at ~2-mm liquid height). From the smallest to the largest liquid height, the data correspond to 2 mL, 5 mL, and 10 mL liquid media. **(B)** Data for a differentiating population that starts with a high density (3448 cells / cm^2). In a 10-mL liquid, this population survives and grows towards the carrying capacity (first data point, at ~2-mm height). From the smallest to the largest liquid height, the data correspond to 10 mL, 20 mL, 30 mL, and 40 mL liquid media. (A) shows that we can rescue a low-density population from becoming extinct if we decrease the liquid height by 50% or more from the usual height of ~2-mm (corresponding to 10 mL). (B) shows that we can drive a high-density population to extinction if we increase the liquid height by a two-fold or more. Together, these results support our finding that local communication - intra-colony or communication between colonies within the same field-of-view - cannot be the primary determinant of a population's collective growth because, as these results suggest, secreted survival-factors diffuse over at least several millimeters to determine a population's survival.

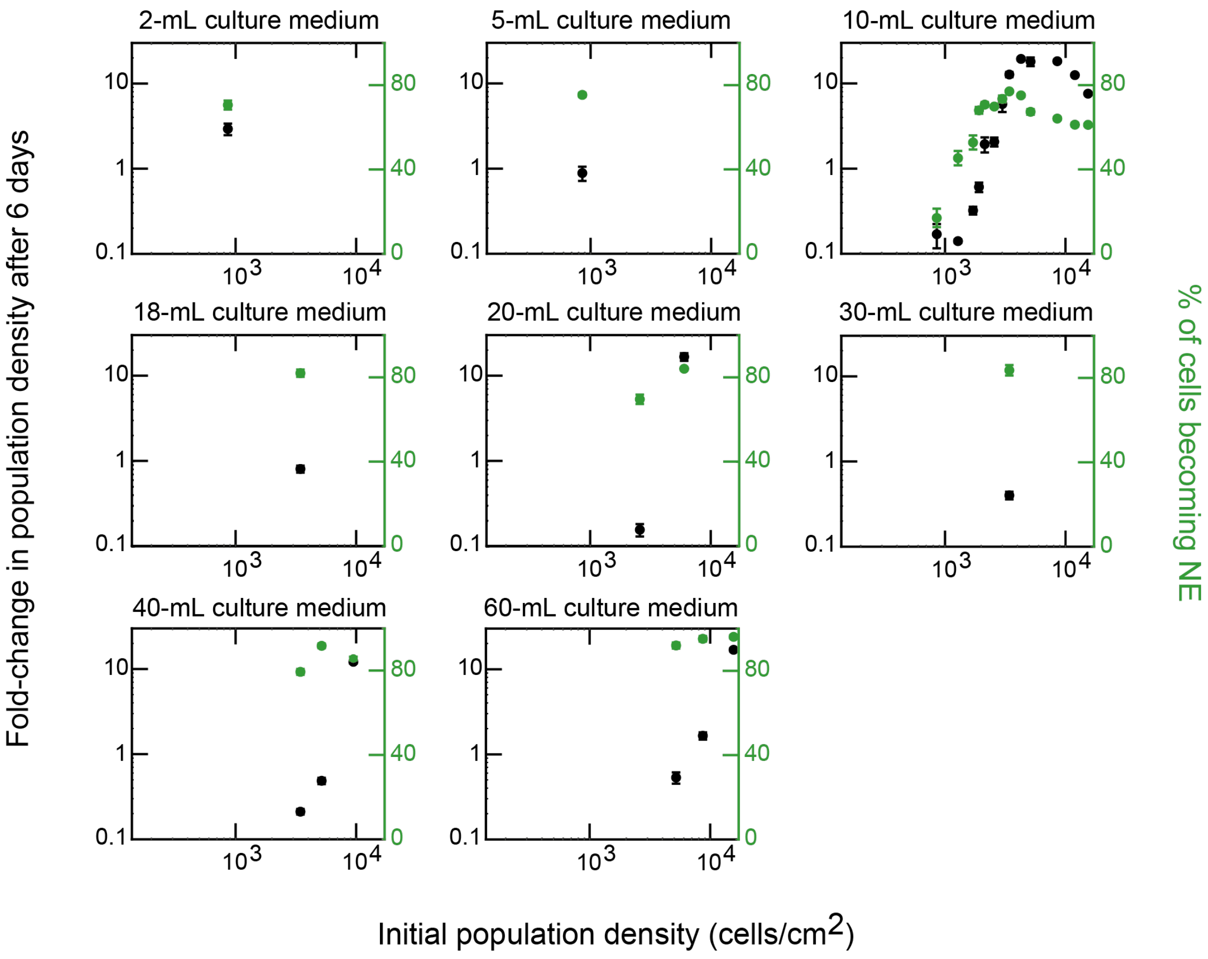

Supplementary Fig. 17 | Volume of liquid medium at the start of differentiation determines collective growth of a population (related to Fig. 5). Data for 46C cells differentiating in N2B27 + RA that were self-renewing in serum+LIF prior to differentiating. To experimentally test the stochastic model's prediction (to verify that the model-produced phase diagram in Fig. 5d is correct), we incubated populations of different starting densities in various volumes of growth medium (and thus in different heights of the liquid medium). Each box shows a different volume of growth medium. Black points show the fold-changes in the population density (relative to the initial density) after six days. Green points show the percentages of cells that entered the NE lineage (Sox1-GFP positive cells measured with flow cytometer). n = 3; Error bars are s.e.m.; n = 3. These results match the phase diagram produced by the model (Fig. 5d).

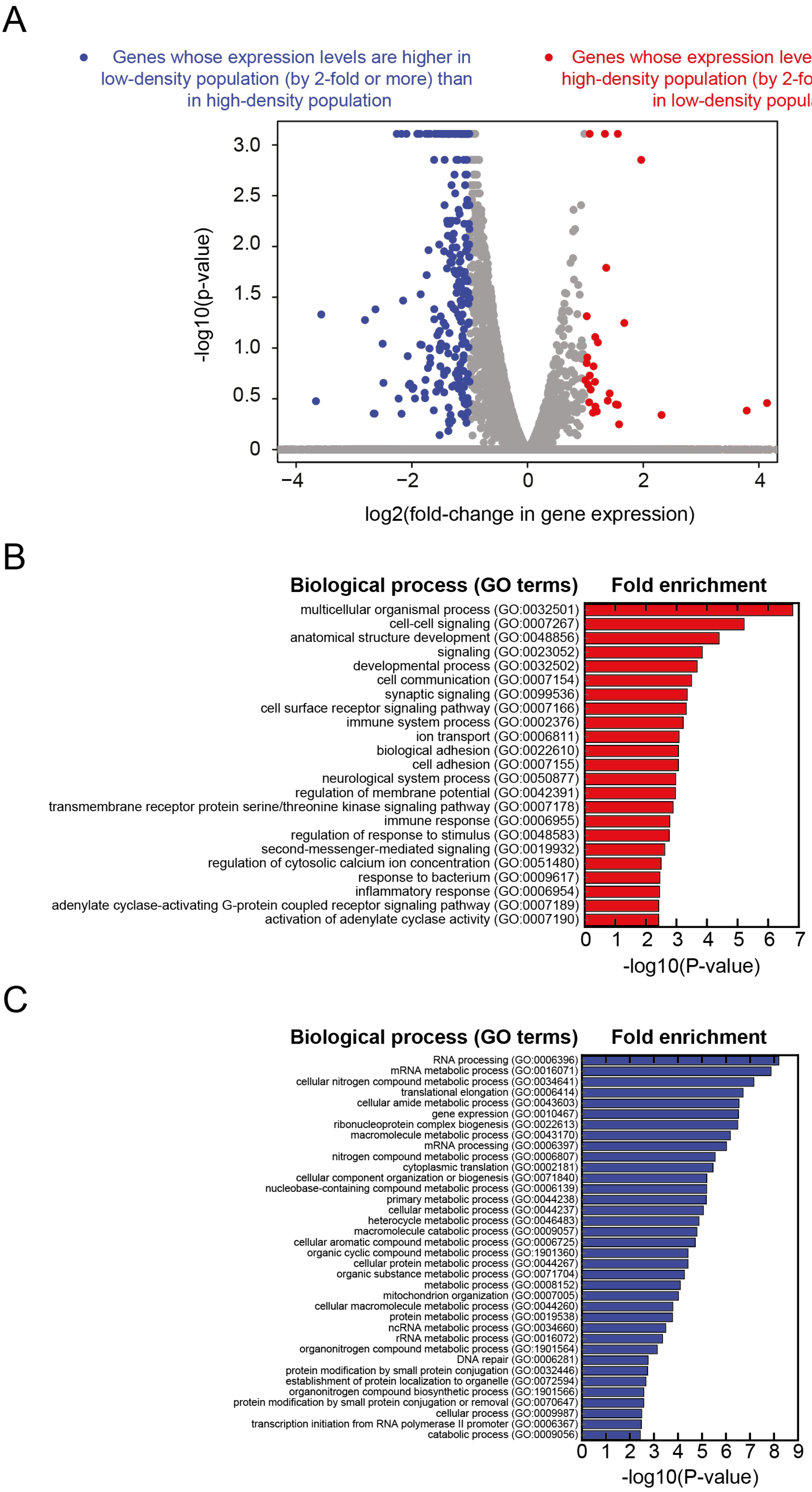
A
Genes whose expression levels are higher in low-density population (by 2-fold or more) than in high-density population
Genes whose expression levels are higher in high-density population (by 2-fold or more) than in low-density population
-log10(p-value)
3.0
2.5
2.0
1.5
1.0
0.5
0
−4
−2
0
2
4
log2(fold-change in gene expression)
B
Biological process (GO terms)
Fold enrichment
multicellular organismal process (GO:0032501)
cell-cell signaling (GO:0007267)
anatomical structure development (GO:0048856)
signaling (GO:0023052)
developmental process (GO:0032502)
cell communication (GO:0007154)
synaptic signaling (GO:0099536)
cell surface receptor signaling pathway (GO:0007166)
immune system process (GO:0002376)
ion transport (GO:0006811)
biological adhesion (GO:0022610)
cell adhesion (GO:0007155)
neurological system process (GO:0050877)
regulation of membrane potential (GO:0042391)
transmembrane receptor protein serine/threonine kinase signaling pathway (GO:0007178)
immune response (GO:0006955)
regulation of response to stimulus (GO:0048583)
second-messenger-mediated signaling (GO:0019932)
regulation of cytosolic calcium ion concentration (GO:0051480)
response to bacterium (GO:0009617)
inflammatory response (GO:0006954)
adenylate cyclase-activating G-protein coupled receptor signaling pathway (GO:0007189)
activation of adenylate cyclase activity (GO:0007190)
0 1 2 3 4 5 6 7
-log10(P-value)
C
Biological process (GO terms)
Fold enrichment
RNA processing (GO:0006396)
mRNA metabolic process (GO:0016071)
cellular nitrogen compound metabolic process (GO:0034641)
translational elongation (GO:0006414)
cellular amide metabolic process (GO:0043603)
gene expression (GO:0010467)
ribonucleoprotein complex biogenesis (GO:0022613)
macromolecule metabolic process (GO:0043170)
mRNA processing (GO:0006397)
nitrogen compound metabolic process (GO:0006807)
cytoplasmic translation (GO:0002181)
cellular component organization or biogenesis (GO:0071840)
nucleobase-containing compound metabolic process (GO:0006139)
primary metabolic process (GO:0044238)
cellular metabolic process (GO:0044237)
heterocycle metabolic process (GO:0046483)
macromolecule catabolic process (GO:0009057)
cellular aromatic compound metabolic process (GO:0006725)
organic cyclic compound metabolic process (GO:1901360)
cellular protein metabolic process (GO:0044267)
organic substance metabolic process (GO:0071704)
metabolic process (GO:0008152)
mitochondrion organization (GO:0007005)
cellular macromolecule metabolic process (GO:0044260)
protein metabolic process (GO:0019538)
ncRNA metabolic process (GO:0034660)
rRNA metabolic process (GO:0016072)
organonitrogen compound metabolic process (GO:1901564)
DNA repair (GO:0006281)
protein modification by small protein conjugation (GO:0032446)
establishment of protein localization to organelle (GO:0072594)
organonitrogen compound biosynthetic process (GO:1901566)
protein modification by small protein conjugation or removal (GO:0070647)
cellular process (GO:0009987)
transcription initiation from RNA polymerase II promoter (GO:0006367)
catabolic process (GO:0009056)
0 1 2 3 4 5 6 7 8 9
-log10(P-value)

Supplementary Fig. 18 | Enrichment analysis of RNA-Seq data reveals that high-density populations, compared to low-density populations, have higher levels of processes (GO terms) such as multicellular organismal processes, cell-cell signaling, neurological system processes, and cell adhesion (related to Extended Data Fig. 4). Data for 46C cells differentiating in N2B27 that were self-renewing in serum+LIF prior to differentiating. We identified possible, intracellular pathways that the secreted survival factors control by performing a transcriptome-level profiling (RNA-Seq) and then looking for differentially expressed genes. We performed RNA-Seq on populations of three different starting densities: (1) a low-density (862 cells/cm^2) population that becomes extinct; (2) a high-density (5172 cells/cm^2) population that grows towards the carrying capacity; and (3) a medium-density (1931 cells/cm^2) population that is near the threshold density. For RNA-Seq, we collected all cells from these populations on the first and second days after triggering differentiation. We also collected cells that were kept pluripotent in a serum-based pluripotency medium (FBS with LIF) as a comparison. We analyzed the resulting transcriptome expression levels (FPKMs) for each gene by focusing on gene-expression levels that differed by more than 2-folds between the high- and low-density populations. **(A)** Volcano plot. Each grey dot represents a single gene. The horizontal axis shows the relative expression level: the expression level of the high-density population divided by the expression level of the low-density population. The vertical axis shows the p-value of the statistical test performed in Cufflinks (Methods). Genes that are more highly expressed by the high-density population than the low-density population, by 2-folds or more, are shown as red points. Genes that are more highly expressed by the low-density population than the high-density population, by 2-folds or more, are shown as blue points. **(B)** Enrichment analysis for genes that are more highly expressed by the high-density population than the low-density population by 2-folds or more (red data points in (A)). We used PANTHER and a custom MATLAB script to examine which GO terms (biological processes) are enriched and determine the corresponding significance (p-value) of the enrichment. We list here the enriched GO terms with their GO-term numbers. This plot shows that GO terms such as “multicellular organismal processes”, “cell-cell signaling”, “neurological system processes” and “cell adhesion” have some of the highest fold enrichments. **(C)** Enrichment analysis for genes that are more highly expressed by the low-density population than the high-density population by 2-folds or more (blue data points in (A)). We list here the enriched GO terms with their GO-term numbers. This plot shows that GO terms such as “RNA processing” and “macromolecule catabolic processes” have some of the highest fold enrichments. See also Supplementary Fig. 19 for further analyses of the RNA-Seq dataset. Supplementary Data lists the genes examined in the RNA-Seq.

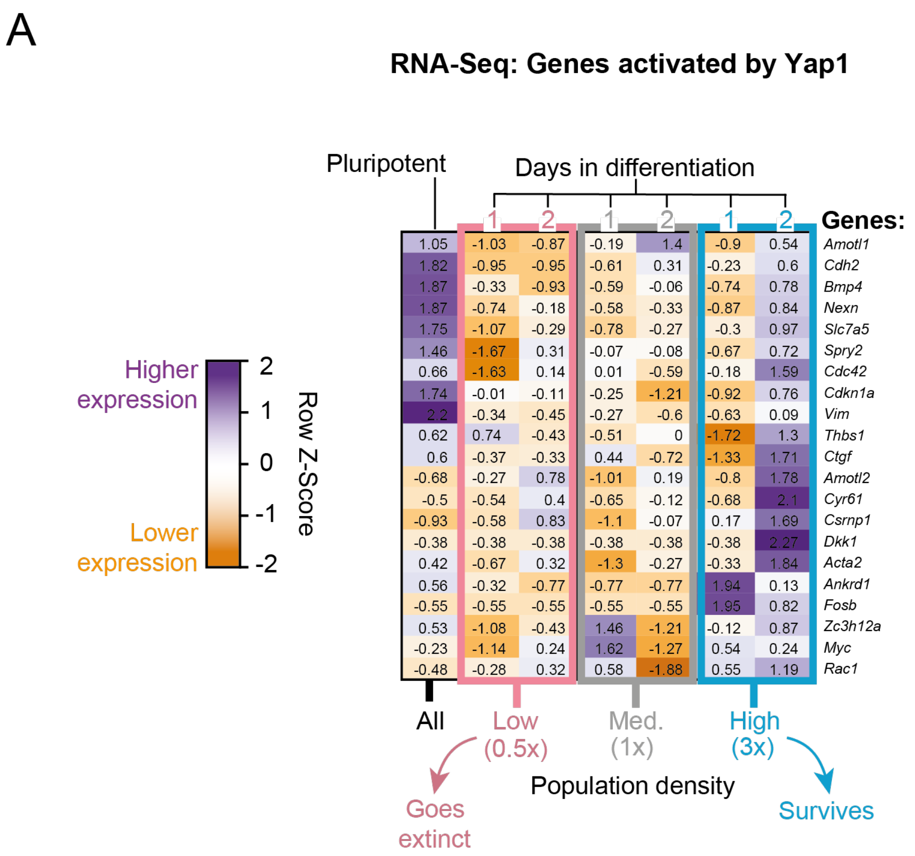

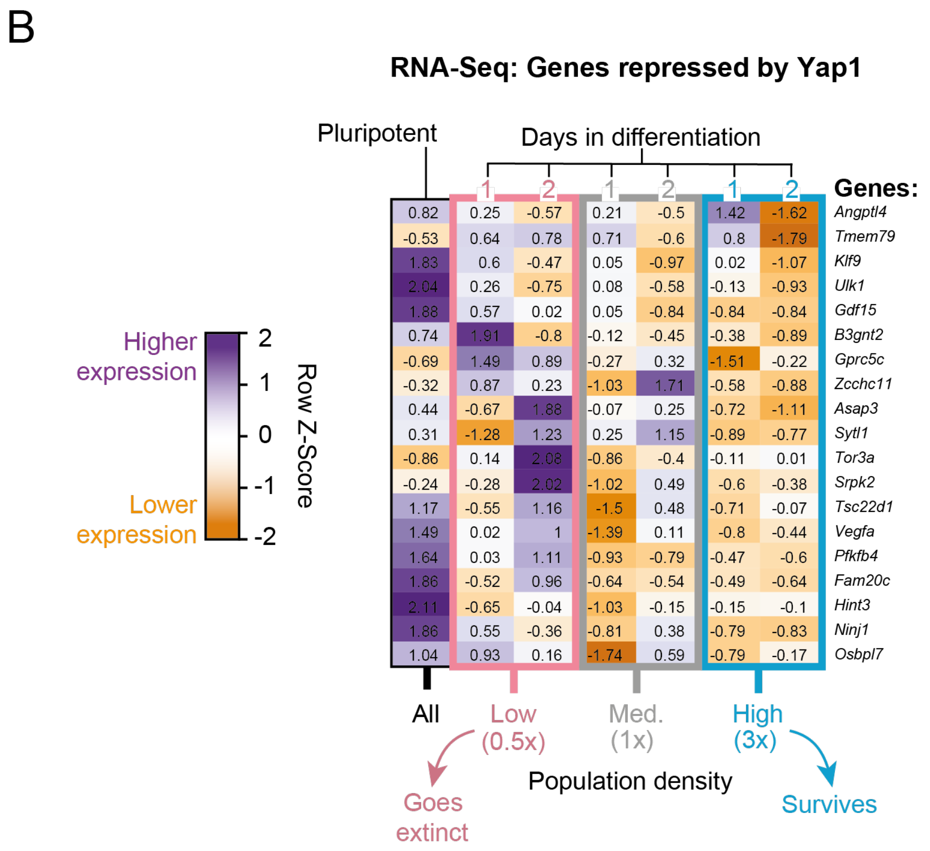

Supplementary Fig. 19 | RNA-Seq analysis reveals that YAP1 becomes more active in populations that start with higher densities (related to Extended Data Fig. 4). Data for

46C cells differentiating in N2B27 that were self-renewing in serum+LIF prior to differentiating. Same RNA-Seq dataset as in Supplementary Fig. 18 and Extended Data Fig. 4. YAP1 is a key component of the Hippo signaling pathway that is important for cell proliferation and apoptosis. We focused on YAP1-related genes because the GO terms that were enriched in the dataset are relevant for YAP1-regulated genes (Supplementary Fig. 18). We found several YAP1-related genes prominently participating in the "cell adhesion" processes (one of the top enriched GO terms). Details of the RNA-Seq are in the caption for Supplementary Fig. 18. In brief, we performed RNA-Seq on populations of three different starting densities: (1) a low-density (862 cells/cm^2) population that becomes extinct; (2) a high-density (5172 cells/cm^2) population that grows towards the carrying capacity; and (3) a medium-density (1931 cells/cm^2) population that is near the threshold density. For RNA-Seq, we collected all cells from these populations on the first and second days after triggering differentiation. We also collected cells that were kept pluripotent in a serum-based pluripotency medium (FBS with LIF) as a comparison. We analyzed the expression levels (FPKMs) by classifying genes into two groups: (1) genes that are activated by YAP1; and (2) genes that are repressed by YAP1[39-45]. For each gene, we computed its mean expression level μ by averaging its expression level across all experimental conditions (i.e., across all densities and days). Afterwards, we determined the row Z-score for each gene and experimental condition, which is a measure of by how much a gene's expression level in each experimental condition deviates from the mean expression level (μ) for that gene. A gene that is more highly expressed has a high row Z-score (close to ~ 2) and is indicated as a shade of purple in the heat maps here. A gene that is more lowly expressed has a low row Z-score (close to -2) and is indicated as a shade of orange in the heat maps here. **(A)** Heat map that shows the row Z-score for each gene (each row) and each experimental condition (each column). These genes are known to be either directly or indirectly activated by YAP1. We observed that these genes, including *Cyr61* and *Amotl2,* were more highly expressed (purple color) by the higher-density populations than the lower-density populations, suggesting that YAP1 is more active in higher-density populations. **(B)** Heat map that shows the row Z-scores for each gene (each row) and each experimental condition (each column). These genes are known to be either directly or indirectly repressed by YAP1. We observed that these genes, including *Angptl4* and *Tmem79*, were more highly expressed (purple color) by lower-density populations than the higher-density populations. Taken together, the results here (A-B) are consistent with YAP1 becoming more active in higher-density populations.

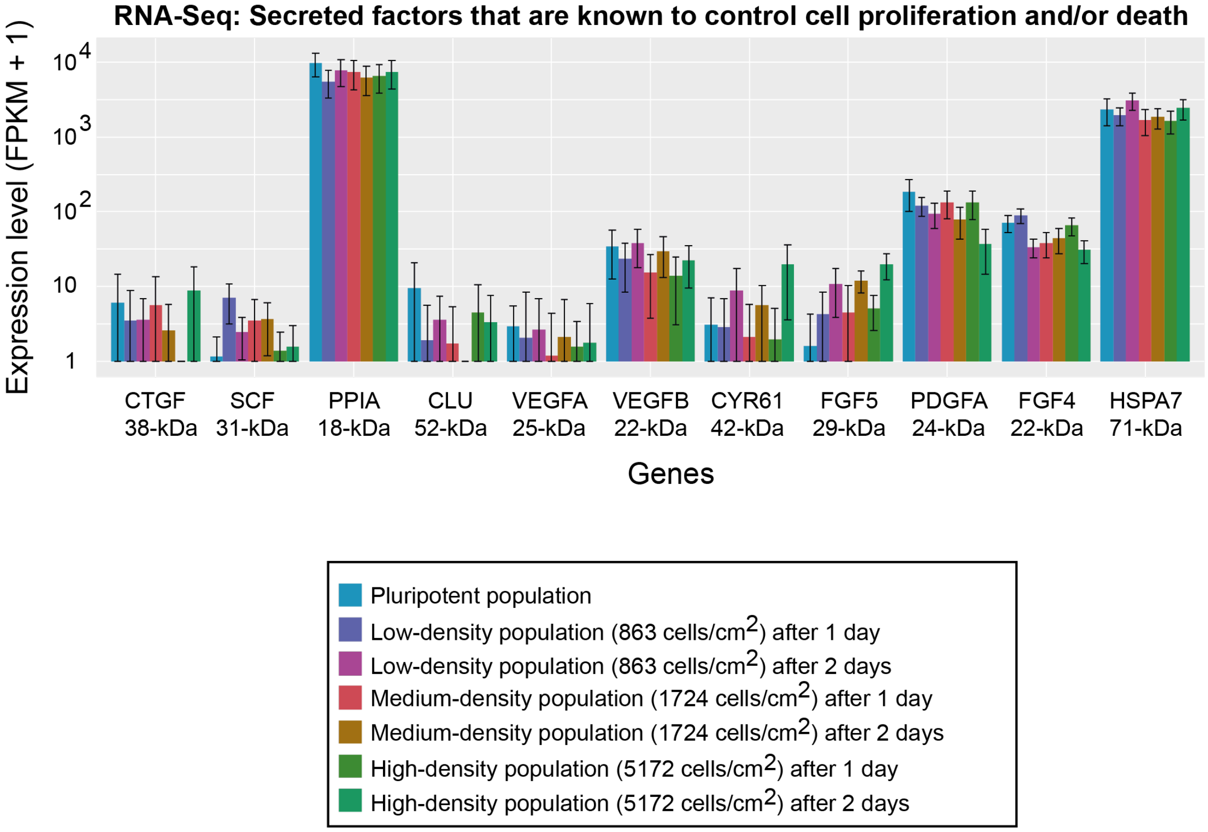

Supplementary Fig. 20 | Expression levels of secreted factors that are known to control cell proliferation (related to Extended Data Fig. 3). Data for 46C cells differentiating in N2B27 that were self-renewing in serum+LIF prior to differentiating. Same RNA-Seq dataset as in Supplementary Fig. 18. To identify secreted ligands that control a population's collective growth, we examined expression levels of any known ligands that are known, from literature, to be a secreted factor and control cell proliferation and/or death. We examined RNA-Seq data from four populations: (1) pluripotent population prior to differentiation; (2) low-density (862 cells/cm^2) population; (3) high-density (5172 cells/cm^2) population; and (4) medium-density (1931 cells/cm^2) population that is near the threshold density. For the three differentiating populations, we collected their cells on the first and second day after triggering differentiation. Expression levels (FPKMs) of secreted factors that are known to control proliferation and/or apoptosis in ES cells and that fall within the range of molecular weights that the membrane-filter experiments identified (50 – 300 kDa with +/-50% error) (Extended Data Fig. 1). Shown are the following genes: *Ctgf*, *Scf*, *Ppia*, *Clu*, *Vegfa*, *Vegfb*, *Cyr61*, *Fgf5*, *Pdgfa*, *Fgf4* and *Hspa8*. Below each gene name is the molecule's weight (kDa) according to two online resources: Uniprot and ExPASy. n = 3 for all plots; Error bars are s.e.m.

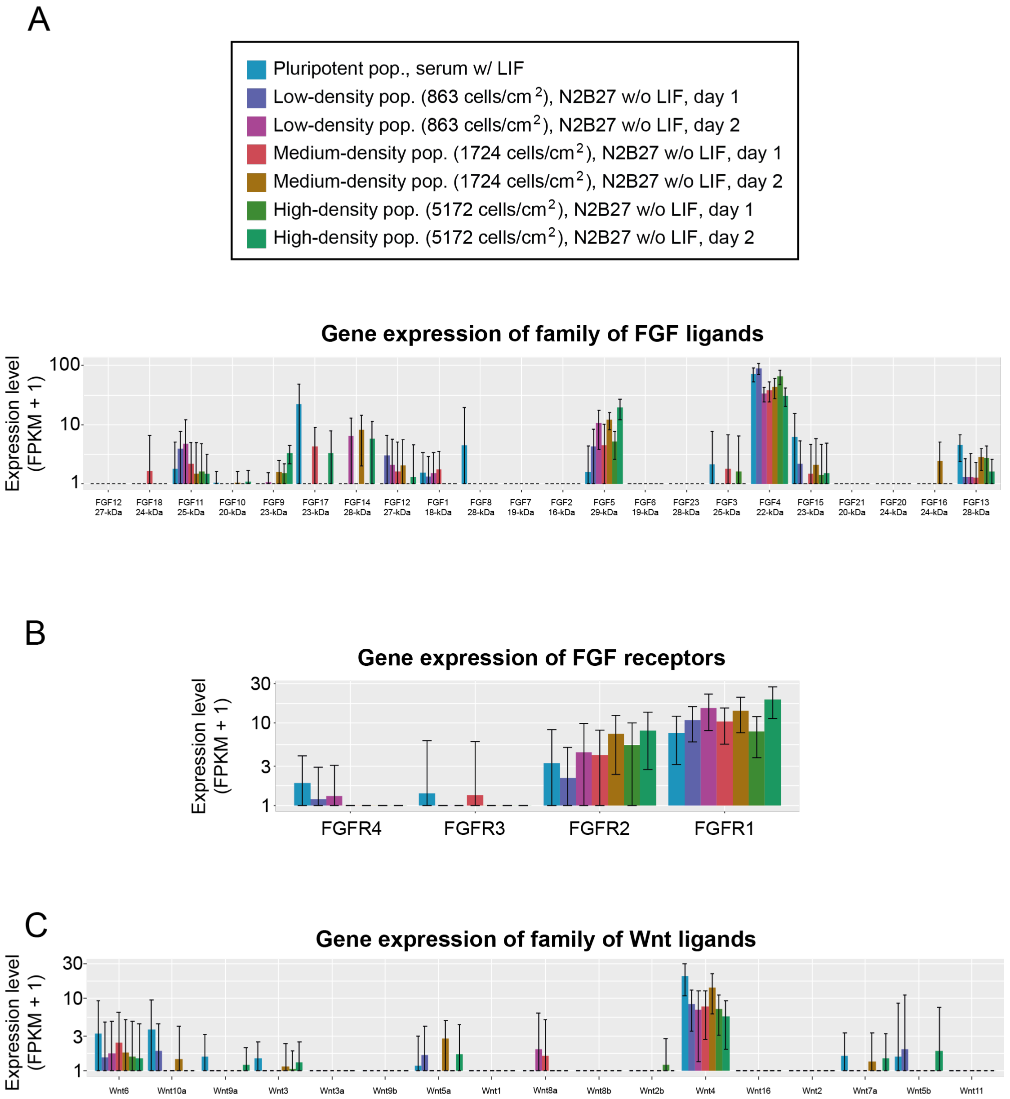

Supplementary Fig. 21 | Expression levels of all 22 FGFs and their receptors (related to Extended Data Fig. 3). Data for 46C cells differentiating in N2B27 that were self-renewing in serum+LIF prior to differentiating. Same RNA-Seq dataset as in Supplementary Fig. 18. We examined RNA-Seq data on four populations: (1) pluripotent population prior to differentiation; (2) low-density (862 cells/cm^2) population; (3) high-density (5172 cells/cm^2) population; and (4) medium-density (1931 cells/cm^2) population that is near the threshold density. For the three differentiating populations, we collected their cells on the first and second day after triggering differentiation. **(A)** Expression levels of all 22 FGF ligands. Shown are the following genes: *Fgf1-8*, *Fgf20-21* and *Fgf23*. Below each gene name is the corresponding molecular weight in kDa. Note that FGF4 expression prominently stands out among all the FGFs. n = 3 for all plots; Error bars are s.e.m. **(B)** Expression levels of all FGF receptors (FGFRs). Shown are the following genes: *Fgfr1-4*. Below each gene name is the corresponding molecular weight in kDa, according to two online resources: Uniprot and ExPASy. n = 3 for all plots; Error bars are

s.e.m. **(C)** Expression levels of Wnt ligands. Shown are the following genes: *Wnt6, Wnt10a, Wnt9a, Wnt3, Wnt3a, Wnt9b, Wnt5a, Wnt1, Wnt8a, Wnt8b, Wnt2b, Wnt4, Wnt16, Wnt7a, Wnt5b* and *Wnt11*. None of the Wnt genes prominently stand out, except for *Wnt4* which still has an order of magnitude lower expression relative to *FGF4* expression. n = 3 for all plots; Error bars are s.e.m.

A

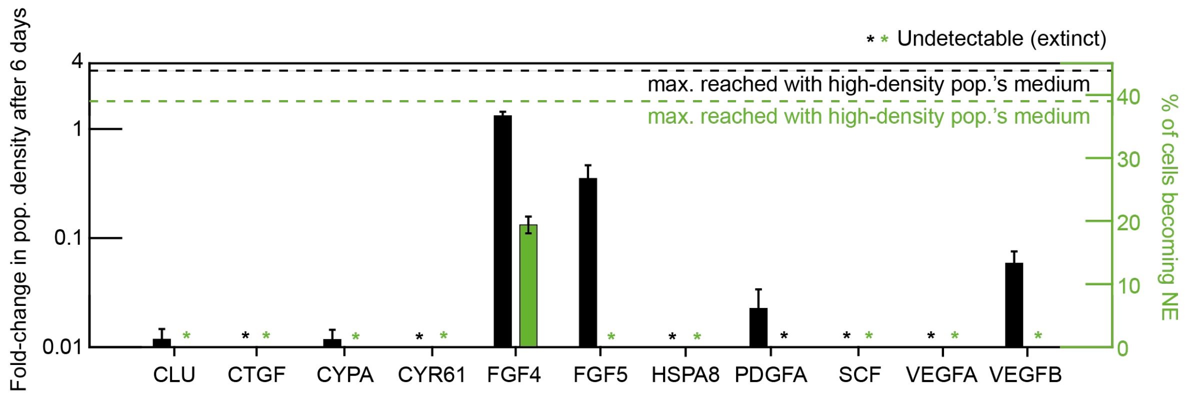

	CLU	CTGF	CYPA	CYR61	FGF4	FGF5	HSPA8	PDGFA	SCF	VEGFA	VEGFB
**11-molecule mix:**	X	X	X	X	X	X	X	X	X	X	X
**6-molecule mix:**			X		X	X	X	X			X
**4-molecule mix:**					X	X		X			X
**2-molecule mix:**					X	X					

B

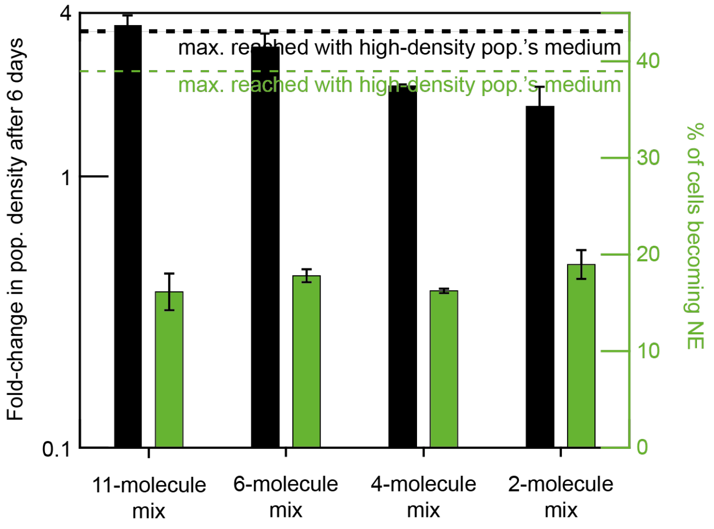

Supplementary Fig. 22 | Out of all the extracellular factors that we added one-by-one into differentiation medium, only FGF4 rescues low-density populations from extinction (related to Extended Data Fig. 3). Supplementary Fig. 20 revealed that 11 secreted factors that are known to control cell proliferation/and or death are highly expressed in high-density populations during differentiation. We thus reasoned that one or combinations of these factors may be the secreted molecule(s) that determine the collective growth of a population. Data in (A) and (B) are for 46C cells differentiating in N2B27+RA that were self-renewing in serum+LIF prior to differentiating. **(A)** We tested the 11 ligands by adding them one-by-one into the medium of a low-density population (862 cells/cm^2) that would ordinarily become extinct. We

added the following molecules individually, each at a saturating concentration (also see Methods): version of recombinant mouse FGF4 used in Supplementary Fig. 24C (200 ng/mL), recombinant human FGF5 (200 ng/mL), recombinant mouse PDGFA (100 ng/mL), recombinant mouse VEGFB 186 (100 ng/mL), recombinant mouse VEGFA (100 ng/mL), recombinant human CYR61 (500 ng/mL), recombinant human CTGF (500 ng/mL), recombinant mouse CLU (200 ng/mL), recombinant human HSPA8 (500 ng/mL), recombinant human CYPA (1000 ng/mL), and recombinant mouse SCF (2000 ng/mL). After 6 days in a medium containing one of these molecules, we measured the fold-change in population-density (black bars) and differentiation efficiency (green bars) of the low-density population. *n* = 3; error bars are s.e.m. These results show that only the recombinant mouse FGF4 causes the fold-change in population density to be higher than one. All the other factors resulted in the low-density population either approaching extinction (fold change much less than 1) or becoming extinct (indicated with an asterisk). The black dashed line marks the maximum fold-change in population density achieved when the low-density population grows in the medium of a high-density population. The green dashed line marks the maximum differentiation efficiency achieved when the low-density population grows in the medium of a high-density population. The box beneath the plot shows which signaling factors were mixed and then given to the low-density population in (B). **(B)** Results obtained by giving combinations of the 11 factors together to the low-density population, with the ingredients of the mixture indicated in the box below (A). Giving all 11 factors together at once yielded the highest growth (~4-fold increase in population density; black bar), which was virtually identical to the growth obtained with a high-density population's (5172 cells/cm^2) medium (black dashed line). But, with the 11 molecules added together at once, the differentiation efficiency (green) remained rather low at ~20% compared to the ~40% (green dashed line) that we get from incubating the low-density population in the medium of a high-density population. As we progressively reduced the number of signaling factors in the mixture from 11 to 2, we observed only a modest decrease in population growth, down to about ~2 fold. Importantly, recombinant FGF4 was included in all these mixtures.

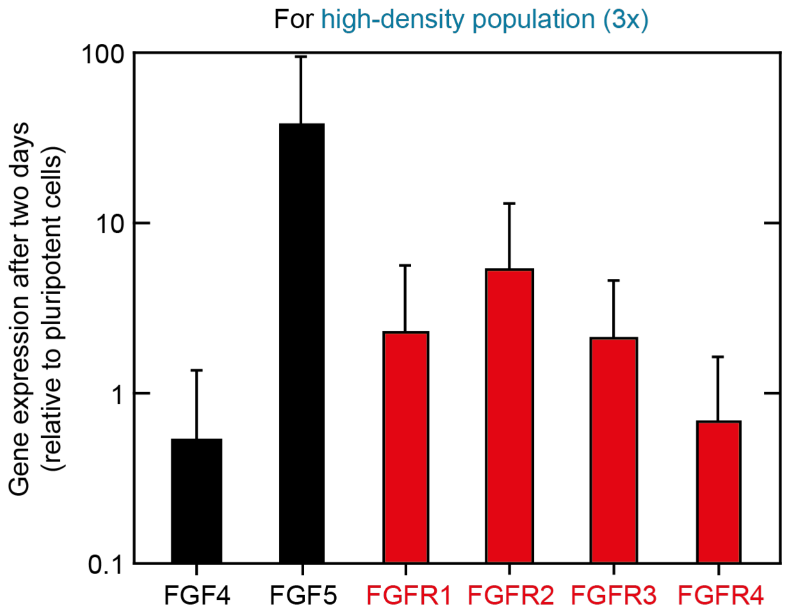

Supplementary Fig. 23 | Differentiating populations express *FGF4*, *FGF5* and all four FGF receptor genes during the first 2 days of differentiation (related to Extended Data Fig. 3 and Fig. 6). Data for 46C cells differentiating in N2B27 that were self-renewing in serum+LIF prior to differentiation. We used RT-qPCR to measure the expression levels of all four receptors (*FGFR1-4*) of Fibroblast Growth Factors (FGFs) and the expression levels of the two FGFs, *FGF4* and *FGF5* (primers in Supplementary Table 2). We examined a high-density population (5172 cells/cm^2) after two days of differentiation. We normalized the resulting expressions of a gene relative to that of the housekeeping gene, *GAPDH* of the same population, and then further normalized the resulting value to the pluripotent population's normalized expression level (like the procedure described in the caption for Supplemetnary Fig. 30). Thus, a given gene's expression level is compared to the pluripotent population's expression level for that gene. Normalized expression levels of *FGF4* and *FGF5* (in black) and *FGFR1*-2 (in red). n = 3; Error bars are s.e.m. Altogether, these results show that *FGF4*, *FGF5* and *FGFR1-2* are expressed – and some more so than the pluripotent population (i.e., expression value greater than 1) – during the first 2 days in which ES cells exit pluripotency.

Standard curves for ELISA on three different versions of FGF4

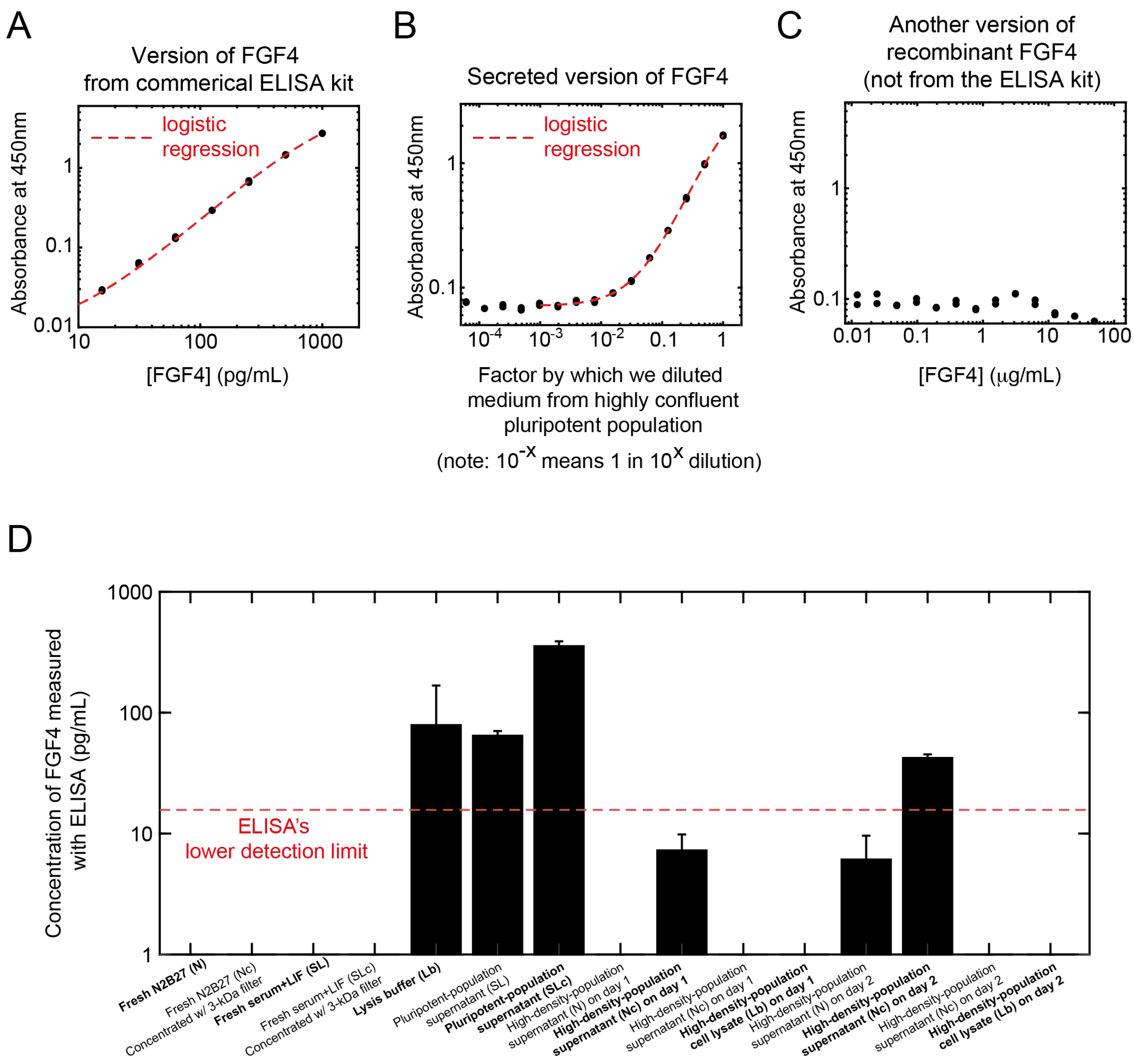

Supplementary Fig. 24 | Cells secrete and extracellularly accumulate appreciable amounts of FGF4 during the first two days of differentiation (related to Fig. 6). We performed ELISA that detected mouse FGF4 (Methods). Data for 46C cells differentiating in N2B27 that were self-renewing in serum+LIF prior to differentiating. **(A)** Standard curve based on a recombinant mouse FGF4 that came with the commercial ELISA kit. Note that this recombinant FGF4 is not necessarily the same version as the FGF4 that our cells secrete. Each measurement (absorbance at 450 nm) was done in duplicate (black data points). Then, we performed a logistic regression on the data by fitting a four-parameter logistic function (red curve): $f(x) = D + \frac{A-D}{1+(\frac{x}{C})^B}$, where *A, B, C* and *D* are constant coefficients and x is the known

concentration of the recombinant mouse FGF4 that we added. We found: A = 0.0084, B = 1.313, C = 1312 and D = 6.59. **(B)** Standard curve based on the version of FGF4 that pluripotent cells secrete into their medium. We first concentrated the medium taken from a highly confluent (~80% confluent) pluripotent population with a 3-kDa filter and then performed ELISA on serially diluted fractions of this concentrated medium. Each measurement (absorbance at 450 nm) was done in duplicate (black points). Then, we performed a logistic regression on the black data points by fitting a 4-parameter logistic function (red curve): $f(x) = D + \frac{A-D}{1+(\frac{x}{C})^B}$, where *A, B, C* and *D* as constant coefficients and x as amount of lysed, pluripotent cells (day 0). We found: A = 0.07123, B = 1.184, C = 1.407 and D = 4.069. The standard curve shows that pluripotent ES cells secrete a version of FGF4 that our ELISA can detect. Moreover, it also shows the ELISA's limitations: the assay could only detect sufficiently high concentration of FGF4 as seen by the fact that it could not detect any FGF4 in a 1:100 dilution of a concentrated medium from a highly confluent ES cells. **(C)** Standard curve based on recombinant mouse FGF4 from a different manufacturer (not from the ELISA kit) that we could add to the cell-culture medium to rescue low-density populations (Methods). Each measurement (absorbance at 450 nm) was taken in duplicates (black points). As seen here, ELISA cannot detect any amounts of this version of FGF4, even when its concentration was 100-folds higher than the highest concentration of the version supplied by the ELISA kit. The three standard curves (A-C) show that ELISA is highly sensitive to the form of FGF4. We used three different forms in each of (A-C). The two versions of FGF4 that were not supplied by the ELISA kit (B-C) were detected with a lower efficiency than the version that was supplied by the kit (A). **(D)** ELISA measurements of secreted FGF4 (in pg/mL) in various conditions (indicated with labels on the horizontal axis). We detected abundant FGF4 in the pluripotency medium (~500 pg/mL). In the medium of the high-density population (8620 cells/cm^2) after two days of differentiation, we detected ~50 pg/mL of FGF4. After 1 day of differentiation, the medium of the high-density population did not contain any detectable amounts of FGF4. Hence, high-density populations take 2 days to accumulate appreciable (detectable) amounts of FGF4.

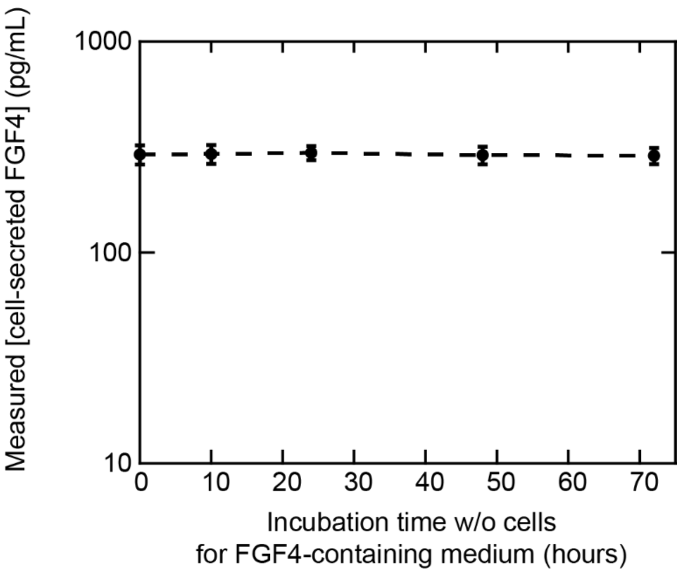

Supplementary Fig. 25 | Secreted FGF4 shows no appreciable degradation for 3 days at 37°C in liquid medium (related to Fig. 6). Determining the degradation rate of FGF4 that cells secrete tells us whether FGF4 can diffuse by millimeters or not, through the Stokes-Einstein equation (Methods). We performed ELISA that targets FGF4 (Methods) to determine the concentrations of secreted FGF4 when incubated without cells in liquid medium. For this, we used a 3-kDa filter to concentration the pluripotency medium (serum+LIF) taken from a confluent population of 46C cells. Then we incubated the medium without any cells in a 37°C incubator for the hours indicated on the horizontal axis. We then took it out of the incubator and performed ELISA on it to measure the remaining [FGF4]. We observed that, after incubating for 72 hours (3 days), the initial concentration of the secreted FGF4 virtually remained unchanged. As a control, we confirmed that no ingredient of a 3-kDa-concentrated pluripotency medium (without cells) interfered with our ELISA measurement to produce a non-zero concentration. Thus, this control showed no such signal (see Supplementary Fig. 24D). Altogether, these results suggests that the concentration of secreted FGF4, by itself and in the absence of cells, is stable over at least 3 days and thus – according to the Stokes-Einstein equation – can diffuse over millimeters (Methods).

A

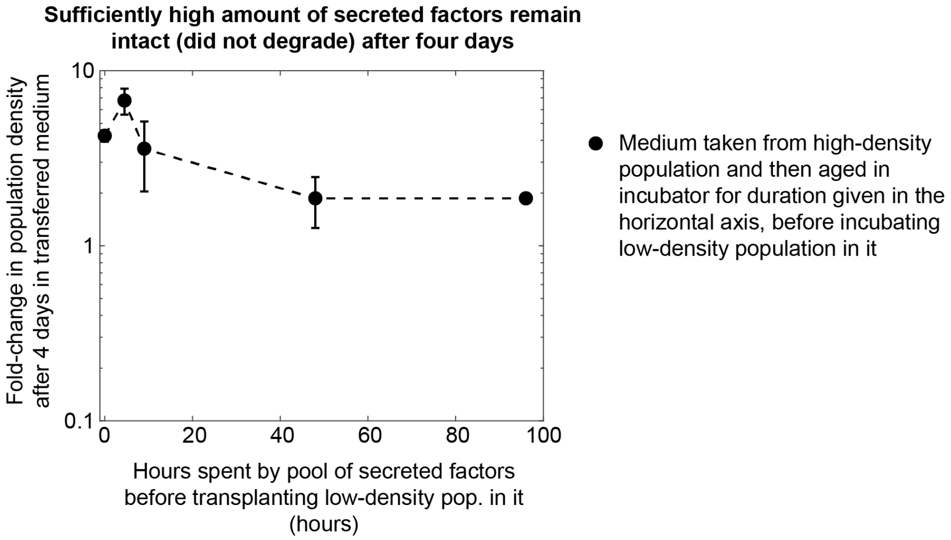

B

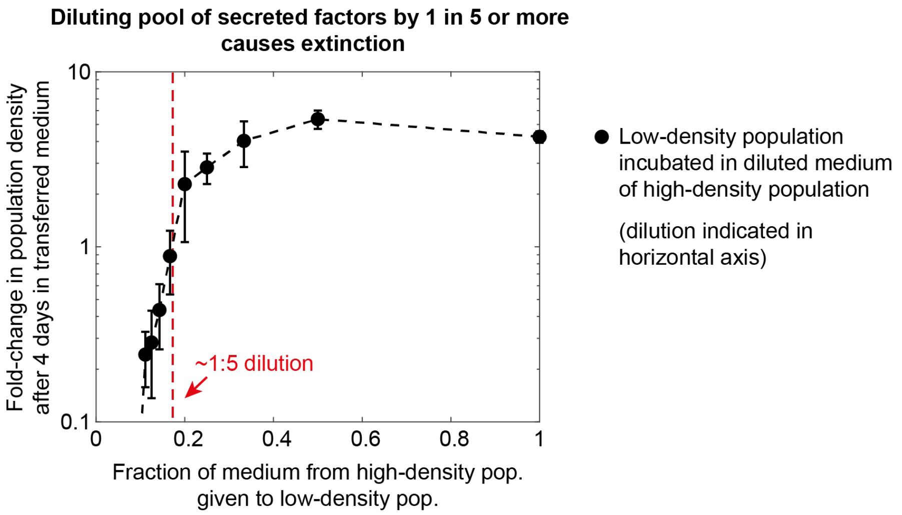

(Note: 0.1 means 1 in 10 dilution of the high-density pop.'s medium into fresh medium)

Supplementary Fig. 26 | Collection of all secreted factors that regulate population's growth have an effective half-life of at least 2 days (related to Fig. 6). Data for 46C cells differentiating in N2B27 that were self-renewing in serum+LIF prior to differentiation. According to the reaction-diffusion equation, molecules of ~100 kDa in an aqueous environment need to have a half-life of at least ~12 hours to have a diffusion length that is over 1 mm (Methods). We sought to infer the half-lives of all the secreted molecules that are important for determining the survival-versus-extinction fate of a population. What is important is not the half-life of each molecule but rather the "effective" half-life of all molecules combined. To determine the effect half-life of all molecules combined, we used two populations: (1) high-density population (5172 cells/cm^2); and (2) low-density population (862 cells/cm^2). Combining the results of (A) and (B) enabled us to infer the lower bound on the effective half-life, as we

now explain. **(A)** After 2 days of differentiation with N2B27, we transferred the high-density population's medium to an empty plate that had no cells. We incubated the medium without any cells in a 37°C incubator for various amounts of time before transferring it to a low-density population that was just ending its second day of differentiation. After 4 days of incubation in the transferred medium (so a total of 6 days of differentiation), we measured the fold-change in density of the low-density population (black points). We plotted the results here as a function of the amount of time the medium spent in the incubator without any cells before we transferred it to the low-density population. The result shows that ageing the medium for 96 hours in 37°C before transferring it to the low-density population still results in rescuing of the low-density population (fold change in population density > 1). The fold change achievable does decrease as the medium's age increases, from ~4-fold (for unaged medium) to ~2-fold (for medium aged for ~96 hours). *n* = 3; error bars are s.e.m. **(B)** In a parallel experiment, we took the medium of the high-density population after two days of differentiation. Then, we diluted it by different amounts into a fresh differentiation medium (one that never harbored any cells). We incubated a 2-days-old low-density population into the diluted medium and then measured the fold-change in its density after four days (so a total of 6 days of differentiation). Plotted here is the fold-change in the population density as a function of how much of the medium from the high-density population was mixed with the fresh medium (e.g., 0.2 on the horizontal axis means a dilution by 1 in 5). The red line shows the maximum dilution that is allowed for still rescuing the low-density population. Any higher dilution causes the low-density population to have a fold-change in density that is less than one. *n* = 3; error bars are s.e.m.

Combining the results of (A) and (B), we can conclude that more than 1/5 of the secreted molecules remain in the medium after four days of ageing in (A) since, for otherwise, the results in (B) tell us that the fold-change in (A) for medium that was aged for 96 hours should be less than 1, which is not the case. In fact, using the same reasoning, we can say that the effective, combined half-life of the secreted molecules is at least two days. To see, this, note that a half-life of one day would mean that after four days, we would have 1/16 of the molecules degraded after four days since $1/2^4 = 1/16$. But 1/16 < 1/5, which would mean that the low-density population should have become extinct in the medium that we aged for 96 hours in (A). This is not the case. Repeating the calculation by assuming that the effective, combined half-life is two days leads to: $1/2^2 = 1/4 > 1/5$, which is consistent with the data in (A). In summary, the effective half-life of all secreted molecules that affect the collective growth is at least two days.

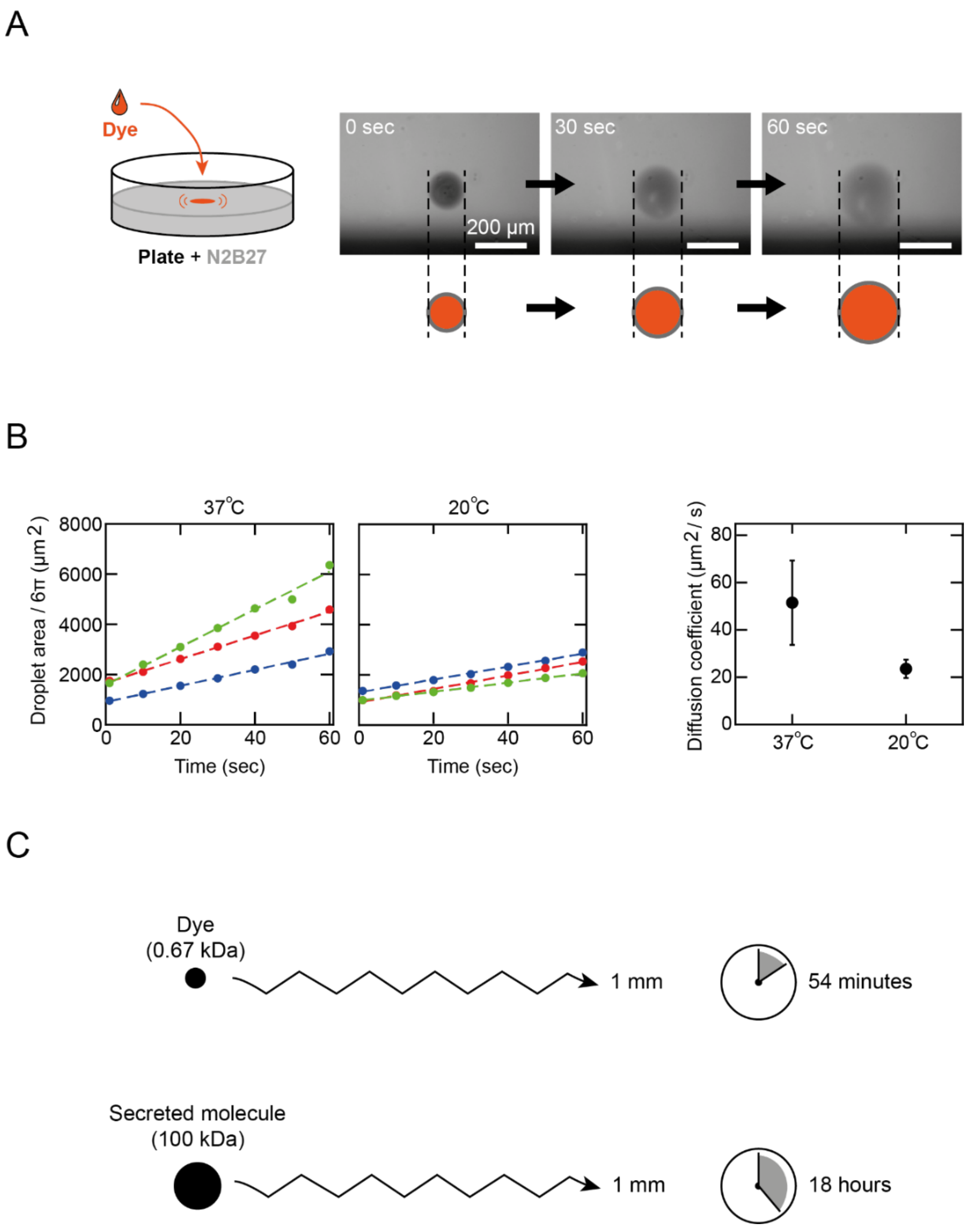

Supplementary Fig. 27 | Diffusion alone, without any other mechanism of transport, explains the long-range (millimeters-scale) spreading of secreted survival factors (related to Fig. 6). To further support the idea that diffusion alone spreads the cell-secreted factors in our experiments, we determined how fast a droplet of a dye molecule of a known weight spreads in a differentiation medium without any cells, under the same incubation conditions as our cell cultures. **(A)** We used a gel loading dye (DNA Gel Loading Dye 6X, Thermo Scientific, #R0611) which consists of two molecules: bromophenol blue (669.96 Da)

and xylene cyanol (538.61 Da). For simplicity, our calculations below will assume that the dye consists of only the heavier molecule, bromophenol blue. We injected a single, 0.5-μL droplet of the dye at the center of a 6-cm diameter plate that contained 5-mL of transparent N2B27 medium, either at room temperature (20°C) or pre-warmed at 37°C. We used a wide-field microscope to make a time-lapse movie with a bird's eye view and snapshots every 10-seconds. Three snapshots (at 0, 30 and 60 seconds; all done at 37°C) of a single droplet of the dye shows the droplet expanding. Scale bar = 200 μm. **(B)** We determined the diffusion constant D of the dye in two ways: using the time-lapse movie and from theory. In the plots, three different colors represent three independent experiments. To determine *D* from the movies, we tracked the visible droplet boundary over time in a movie to plot the droplet area over time (shown in the two plots here at 37°C and 20°C). As shown, the droplet area linearly increased over time, which is consistent with pure diffusion (pure Brownian motion) since the area of a droplet is proportional to the mean squared displacement of a particle. Specifically, for a particle that undergoes a pure three-dimensional diffusion (Brownian motion), its mean squared displacement $\langle R^2 \rangle$ at time *t* is: $\langle R^2 \rangle = 6Dt$. Let *A* be the 2-dimensionally projected area of the droplet. Then, $\langle R^2 \rangle = \frac{A}{\pi}$ and hence, $D = \frac{A}{6\pi t} = \frac{A_{slope}}{6\pi}$, where A_{slope} is the slope of the linear fits to the droplet area as shown in the two plots here. From these fits, the experimentally determined diffusion constants D_{exp} at 37°C and 20°C are 51.5 ± 17.8 μm^2/s and 23.5 ± 3.9 μm^2/s respectively (*n* = 3; error bars are s.e.m.). As a comparison, we determined the diffusion constant D from theory - via the Stokes-Einstein equation which states, $D = \frac{kT}{6\pi\eta r_{dye}}$ where k is the Boltzmann constant, T is temperature, η is the medium's dynamic viscosity, and r_{dye} is the radius of the dye molecule. For water, η = 0.000692 kg/m·s at 37°C and η = 0.001003 kg/m·s at 20°C (from BioNumbers - Milo et al. *Nucleic Acids Res* (2010)). We conservatively estimated r_{dye} by noting that bromophenol blue consists of ~10 carbon-carbon bonds which would mean that the dye molecule's diameter is 10 x 0.126 nm. For simplicity, we assume that $r_{dye} = 1\ nm$. The Stokes-Einstein equation then states that the dye's diffusion constants D_{theory} at 37°C and 20°C are 328.1 μm^2/s and 214.0 μm^2/s respectively. Hence, D_{exp} < D_{theory}. The fact that our analysis relies on the visible (by eye) boundary of the expanding droplet would underestimate the D_{exp} since the dye must be spreading at least as fast as the boundary does. More importantly, if there were significant convection currents in the liquid medium, then D_{exp} would be much larger than the measured value. This argues against there being any significant liquid convection in our cell-culture media. In other words, the dye-based experiment strongly indicates that cell-secreted survival-promoting factors spread out by pure diffusion rather than by convection currents which, according to the dye, are negligible in our cell-culture conditions. Furthermore, note that

$\frac{D_{exp}(37^oC)}{D_{exp}(20^oC)}$ = ~2.2x and $\frac{D_{theory}(37^oC)}{D_{theory}(20^oC)}$ = ~1.5x; the experimental and theoretical values for pure diffusion closely match (proportional to a factor on the order of one). **(C)** Based on Brownian motion in 3 dimensions with the experimentally determined diffusion constant, the dye molecule has a mean squared displacement of $\langle R^2 \rangle$ = 1 mm^2 after $t_{dye} = \frac{\langle R^2 \rangle}{6D}$ = 54 minutes (with $D = D_{exp}(dye, 37^oC)$ = 51.5 μm^2/s). The same calculation, but now based on the Stokes-Einstein estimate of the diffusion constant would yield t_{dye} = 8.5 minutes for a 1 mm^2 mean squared displacement (with $D_{theory}(dye, 37^oC)$ = 328.1 μm^2/s). Hence the theory predicts a faster spreading of dye than experimentally observed - again, arguing against liquid convection or any other mechanism besides diffusion helping to spread the dye. A secreted molecule of 100 kDa would have a mean squared displacement of 1 mm^2 after time, $t_{secreted} = \frac{\langle R^2 \rangle}{6D_{secreted}} = \frac{\langle R^2 \rangle \cdot r_{secreted}}{6 \cdot D_{exp}(dye, 37^oC) \cdot r_{dye}}$ = 18 hours, where we estimate the radius of the molecule to be $r_{secreted}$ = 20 nm (Methods). Hence the two days taken to observe appreciable amount of FGF4 and other survival-promoting factor(s) traveling millimeters and accumulating is consistent with the $t_{secreted}$ calculated here (i.e., if $t_{secreted}$ were much larger than two days, then we should not be observing the survival-factors travelling by millimeters within two days).

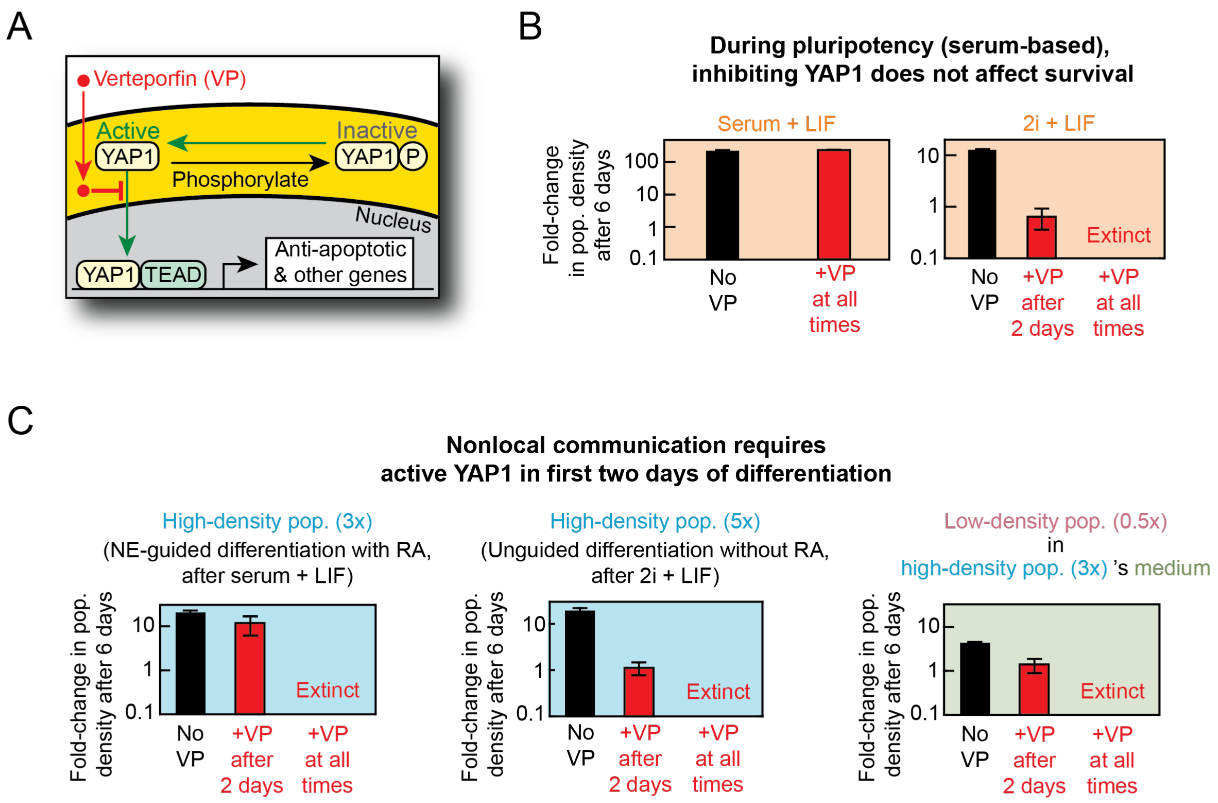

Supplementary Fig. 28 | Macroscopic quorum-sensing requires active YAP1 in first two days of differentiation but, during self-renewal in serum-based medium, inhibiting YAP1 does not affect cell survival (related to Fig. 6). (A) Cartoon shows YAP1 which exists as either phosphorylated (labeled "P") or dephosphorylated. Verteporfin (VP) is a well-characterized inhibitor of active YAP1[39,44] and thus inhibits active (dephosphorylated) YAP1 from entering the nucleus and regulating target gene expression and thus anti-apoptotic processes. **(B)** Data for 46C cells self-renewing in either serum+LIF (left graph) or 2i+LIF (right graph) (Methods). (Left graph) Fold-change in population density for low-density population (862 cells / cm2 initially) after 6 days of self-renewal in serum+LIF in the absence of VP (“No VP”, black bar) or presence of 1 μM VP at day 0 (“+VP at all times”, red bar). The result shows that addition of VP, and hence the inhibition of any active YAP1, does not affect survival and growth of serum-grown cells. (Right graph) Fold-change in population density for low-density population (862 cells / cm2 initially) after 6 days of self-renewal in 2i+LIF in the absence of VP (“No VP”, black bar) or presence of 1 μM VP at day 0 (“+VP at all times”, red bar) or day 2 (“+VP after 2 days”, red bar). Error bars are s.e.m.; n = 3. The result shows that addition of VP, and hence the inhibition of any active YAP1, does not affect survival and growth of only serum-grown pluripotent cells. **(C)** Data for 46C cells differentiating towards NE lineage in N2B27 with or without RA that were previously self-renewing in either serum+LIF or 2i+LIF (Methods). Left most and right most graphs are identical to the graphs shown in Fig. 6c and

involved 46C cells previously self-renewing in serum+LIF and then differentiating in N2B27+RA. (Middle graph) Fold-change in population density for high-density population (8621 cells / cm2 initially, previously self-renewing in 2i+LIF) after 6 days of unguided differentiation in N2B27 without RA in the absence of VP ("No VP", black bar) or presence of 1 µM VP at day 0 ("+VP at all times", red bar) or day 2 ("+VP after 2 days", red bar). Error bars are s.e.m.; n = 3. The result shows that – regardless of the use of serum during self-renewal and regardless of the use of RA during differentiation – nonlocal communication requires active YAP1 during the first two days of differentiation.

A

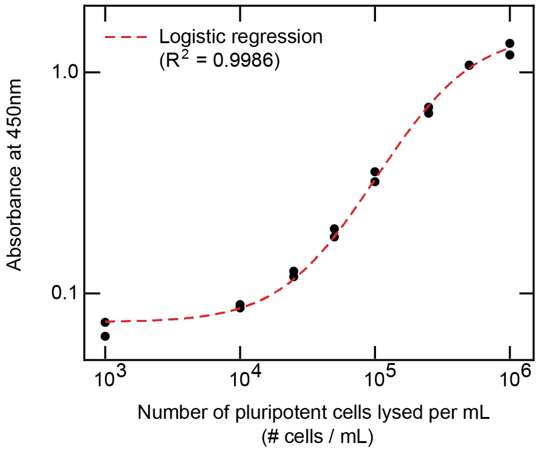

B

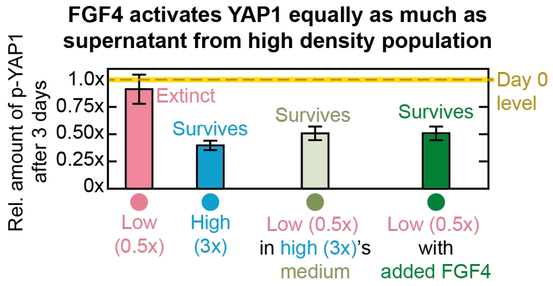

C

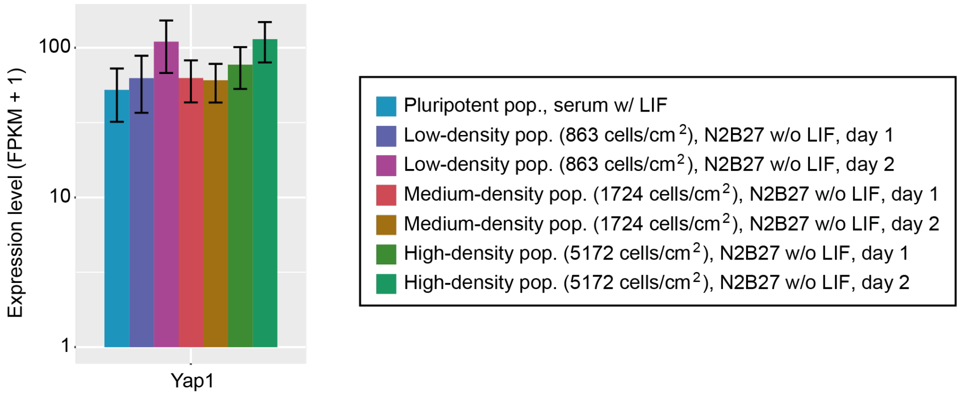

Supplementary Fig. 29 | Populations that survive differentiation have more active (dephosphorylated) YAP1 compared to populations that become extinct during differentiation (related to Fig. 6). Data in (A) and (B) for 46C cells differentiating in

N2B27+RA that were self-renewing in serum+LIF prior to differentiation. To determine how the YAP1 activity may be determined by the population density, we performed ELISA that specifically detects inactive (phosphorylated) YAP1 - YAP1 phosphorylated at Ser397, which is a primary phosphorylation site[41,42] (Methods). **(A)** Standard curve for the ELISA. Here we lysed pluripotent populations of various densities and then measured the amount of phosphorylated YAP1 in each lysate - this yields an optical absorbance value at 450 nm (black points). Duplicates for each lysate are shown. Red curve is the logistic fit function: $f(x) = D + \frac{A-D}{1+(\frac{x}{C})^B}$, with *A, B, C* and *D* are fit constants and x is the number of cells lysed per mL. Results: A = 0.074, B = 1.418, C = 2.95 x 10^5 and D = 1.502 with an R^2 = 0.9986. **(B)** We examined three populations: (1) high-density population (5172 cells/cm^2); (2) low-density population (862 cells/cm^2); and (3) low-density population that we rescued from extinction by transplanting it, after two days, into the high-density population's medium. For each population, we measured its level of phosphorylated (inactive) YAP1 three days after starting differentiation and then normalized this level to the level present in pluripotent cells of the same density (shown in (A)). This yielded a "relative abundance" for each of the three populations. Compared to the pluripotent cells of equivalent density, cells of the low-density population (pink bar) had ~10% fewer inactive YAP1 whereas cells of the high-density population (blue bar) had ~60% fewer inactive YAP1 than the pluripotent population of the same density. Cells of the rescued low-density population (green bar) had ~50% (green bar) less inactive YAP1 than pluripotent populations of the same density. Together, these results establish that, after exiting pluripotency, cells of surviving populations have more active (dephosphorylated) YAP1 than cells that head towards extinction. **(C)** Data for 46C cells differentiating in N2B27 that were self-renewing in serum+LIF prior to differentiation (Methods). Expression level of YAP1 from RNA-Seq dataset. Legend shows different conditions. Note that on each day, the low-density (862 cells/cm^2 initially) and the high-density (5172 cells/cm^2 initially) populations have virtually the same YAP1 expression level. Thus, we can compare the amounts of inactive (phosphorylated) YAP1 between the low- and high-density populations in Fig. 6c (i.e., since both populations have nearly the same total level of YAP1, we would be subtracting the amount of inactive YAP1 from the same value for both populations to get the amount of active Yap1). Medium-density (1931 cells/cm^2 initially) population starts with the near-threshold density. n = 3 for all plots; Error bars are s.e.m.

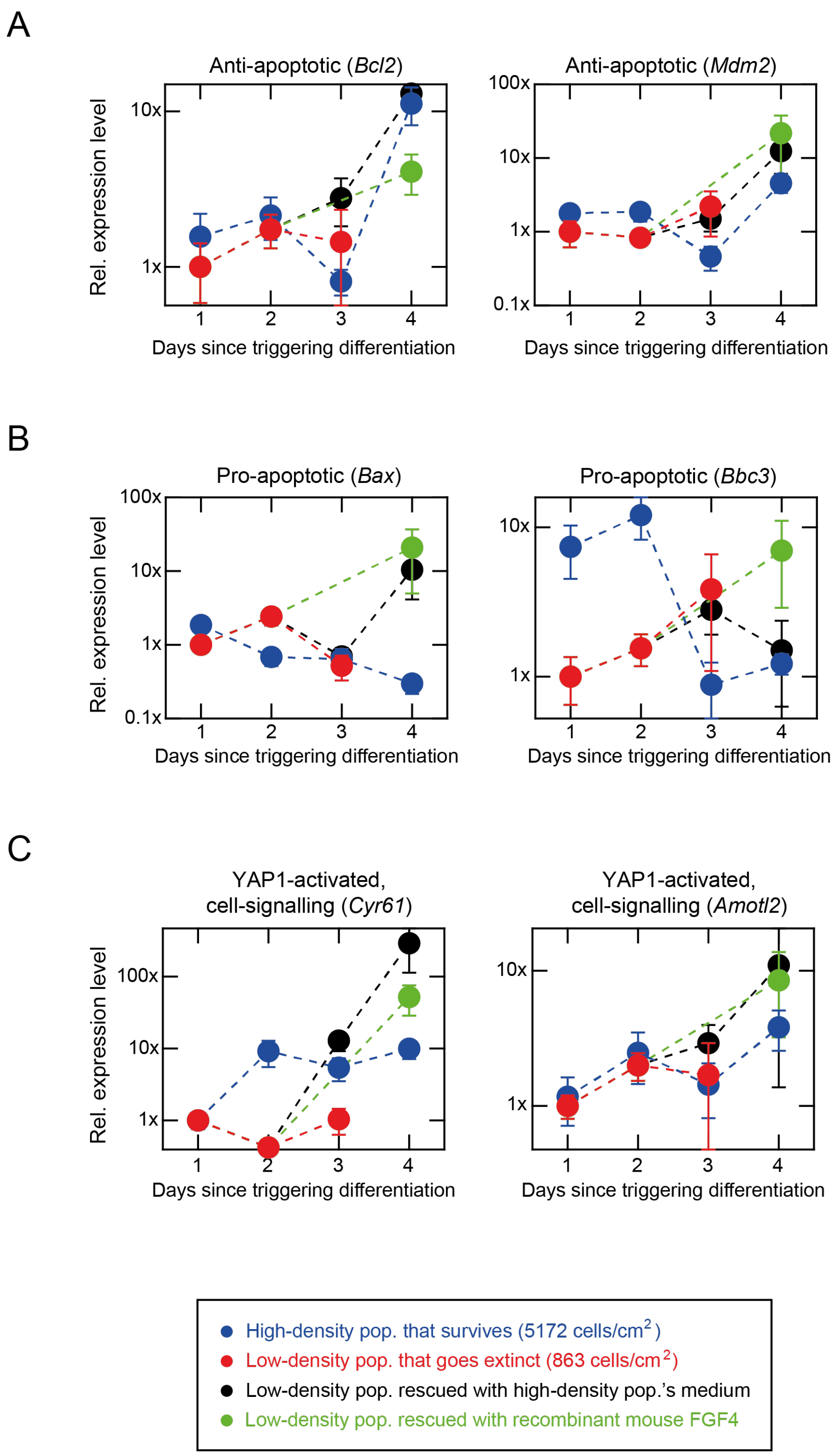

Supplementary Fig. 30 | Low-density populations that are rescued from extinction with recombinant mouse FGF4 or high-density population's medium activate YAP1-regulated genes (related to Fig. 6). Data for 46C cells differentiating in N2B27+RA that were self-renewing in serum+LIF prior to differentiation. With real-time quantitative PCR (primers in Supplementary Table 2), we measured anti-apoptotic, pro-apoptotic, and YAP1-mediated cell-

signaling genes over the course of differentiation. Normalization of expression values: for each gene *g*, we first divided its expression level by the expression level of *Gapdh*, resulting in a value N_g. For each population, we divided its N_g by the low-density population's N_g on day 1 to get the final, normalized expression level μ which is plotted in here in all graphs. Thus, "1x" is the expression level of the low-density population on the first day after starting differentiation. We examined four populations: (1) high-density population (5172 cells/cm^2); (2) low-density population (862 cells/cm^2); (3) low-density population that we rescued from extinction by transplanting it, after two days, into the high-density population's medium; and (4) low-density population that we rescued after adding 200 ng/mL recombinant mouse FGF4 to its differentiation medium on day 0. (**A)** Expression levels of two anti-apoptotic genes, *Bcl2* (left graph) and *Mdm2* (right graph). Both *Bcl2* and *Mdm2* show increased expressions (more anti-apoptotic) for high-density population (blue) and low-density population that was rescued by the medium of the high-density population after the 2nd day (black) or with FGF4 (green). Low-density population that goes extinct (red) shows nearly constant, low expression level of both genes. No data for 4th day is shown for the low-density population because it becomes extinct after the 3rd day (there were already barely any cells left for the 3rd day data shown here). **(B)** Expression levels of two pro-apoptotic genes - *Bax* (left graph) and *Bbc3* (right graph). Color scheme is the same as in (A). The high-density population initially has a higher Bbc3 expression than the low-density population but eventually down-regulates and has lower Bbc3 expression than the low-density population. The low-density population, in turn, gradually increases its Bbc3 expression over time, up to the moment of extinction (~ day 3). Note that the rescued low-density population keeps its Bbc3 expression level low, past day 2 (which is when it receives the medium from a high-density population) and has nearly same low Bbc3 expression as the high-density population after being rescued. Note that differentiation is known to increase expression of apoptotic genes. **(C)** Expression levels of two cell-signaling genes that are upregulated by Yap1, *Cyr61* (left graph) and *Amotl2* (right graph). Only the high-density and the rescued low-density populations gradually increase the expression levels of both genes whereas the low-density population that heads towards extinction (red) maintains a nearly constant, low expression of both genes (consistent with our findings in Fig. 6 that secreted factors that are abundant for high-density populations increase YAP1 activity (and thus upregulate expression of *Cyr61* and *Amotl2*). In all the plots, n = 3; Error bars are s.e.m.

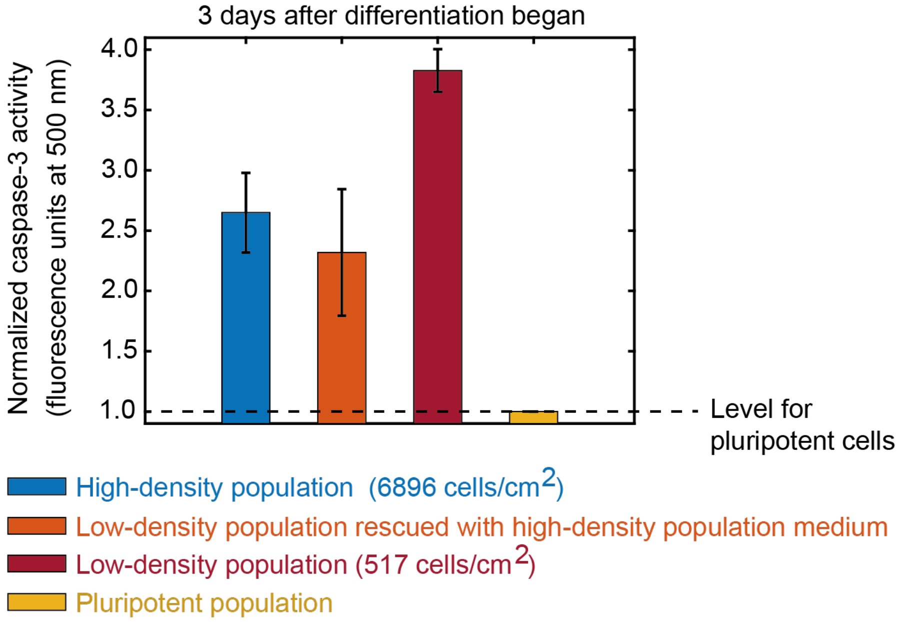

Supplementary Fig. 31 | Pro-apoptotic caspase-3 activity is higher in low-density populations than in high-density and rescued low-density populations (related to Fig. 6). Data for E14 cells differentiating in N2B27+RA that were self-renewing in serum+LIF prior to differentiation. We sought to examine the activity of a well-known, pro-apoptotic marker (caspase 3) in populations that are either extinction-bound or surviving. We used a membrane-permeable, DNA-dye-based assay (NucView 488 Caspase-3 Assay Kit for Live Cells). This assay measured the amounts of active caspase 3/7 inside cells. We examined caspase-3 levels in four different populations: (1) high-density population (6896 cells/cm^2 –blue bar); low-density population (517 cells/cm^2 –red bar); (3) low-density population that was rescued from extinction by transplanting it, after two days, into the high-density population's medium (orange bar); and (4) pluripotent E14 cells before we induced the differentiation (yellow bar). After 3 days of differentiation, we collected the cells from each of these populations, mixed them with the DNA-dye according to the manufacturer's protocol, and then measured the resulting fluorescence at 500 nm in single cells with a flow cytometer. Plotted here are the geometric means of the fluorescence for each population. Higher fluorescence means more caspase-3 activity. We normalized the values to the fluorescence level of the pluripotent population (yellow bar), as indicated by the dashed line. *n* =3; Error bars are s.e.m. This plot shows that all differentiating populations - including the very high-density populations - have upregulated levels of caspase-3 activity relative to the pluripotent populations. Note that differentiation is known to increase expression of apoptotic genes.

Supplementary Table 1 | Ingredients for the differentiation medium (N2B27) and their molecular weights (Related to Fig. 5).

Our recipe for making N2B27 (differentiation medium) is based on established protocols (Ying et al[37]) and is summarized in Methods. Below we list the ingredients of N2B27 together with their molecular weights (MW). This list is adapted from Mittal and Voldman[16] and based on information from ATCC, Ying et al.[37], and Brewer et al. (*J Neurosci Res* (1993)). Most components of differentiation medium (N2B27) are smaller than the smallest filter size that we used (3 kDa), and if larger then not vital for growth of ES cells (see Mittal and Voldman[16]). We show experimentally that filtering N2B27 does not catch any ingredients vital for ES cell growth (see Supplementary Fig. 15)

Abbreviations: MW = molecular weight, Da = Daltons, N/A = not applicable (i.e., not included in mixture), CONF = confidential, propriety information (Invitrogen).

Compound Name	MW (Da)	Conc. in DMEM/F-12 (μM)	Conc. in Neurobasal (μM)	Conc. In N2 supplement (μM)	Conc. In B-27 minus vitamin-A supplement (μM)
Inorganic salts					
CaCl2 (anhydrous)	111	1000	1800	N/A	N/A
CuSO4 (anhydrous)	160	0.01	N/A	N/A	N/A
Fe(NO3)3·9H2O	404	0.13	0.25	N/A	N/A
FeSO4·7H2O	278	1.5	N/A	N/A	N/A
MgSO4 (anhydrous)	120	700	812	N/A	N/A
KCl	75	4000	5333	N/A	N/A
NaHCO3	84	14,300	26,000	N/A	N/A
NaCl	58	120,000	51,300	N/A	N/A
Na2HPO4 (anhydrous)	142	500	N/A	N/A	N/A
NaH2PO4 ·H2O	120	500	1,000	N/A	N/A
ZnSO4·7H2O	288	1.5	N/A	N/A	N/A
Amino Acids					
L-Alanine	89	50	22.5	N/A	N/A
L-Arginine·HCl	210	700	400	N/A	N/A
L-Asparagine·H2O	150	50	5	N/A	N/A
L-Aspartic Acid	133	50	N/A	N/A	N/A
L-Cystine·HCl·H2O	294	60	N/A	N/A	N/A
L-Cystine·2HCl	312	100	10	N/A	N/A
L-Glutamic Acid	147	50	N/A	N/A	N/A
L-Glutamine	146	2,500	500	N/A	N/A
Glycine	75	250	400	N/A	N/A
L-Histidine·HCl·H2O	209	150	200	N/A	N/A
L-Isoleucine	131	415	800	N/A	N/A
L-Leucine	131	450	800	N/A	N/A
L-Lysine·HCl	146	625	1,000	N/A	N/A
L-Methionine	149	115	200	N/A	N/A

L-Phenylalanine	165	215	400	N/A	N/A
L-Proline	115	150	67	N/A	N/A
L-Serine	105	250	400	N/A	N/A
L-Threonine	119	450	800	N/A	N/A
L-Tryptophan	204	45	80	N/A	N/A
Vitamins					
D-Biotin (B7)	244	0.01	CONF	N/A	N/A
Choline Chloride	140	64	28	N/A	N/A
Folic Acid (B9)	441	6	9	N/A	N/A
myo-Inositol (B8)	180	70	40	N/A	N/A
Niacinamide (B3)	122	16.5	33	N/A	N/A
D-Pantothenic Acid (B5)	219	10	8	N/A	N/A
Pyridoxine·HCl (B6)	202	10	20	N/A	N/A
Riboflavin (B2)	376	0.6	1	N/A	N/A
Thiamine·HCl (B1)	373.5	6	10	N/A	N/A
Vitamin B-12	1,355	0.5	0.25	N/A	N/A
Proteins					
Insulin	5,800	N/A	N/A	4.3	CONF
Serotransferrin	77,000	N/A	N/A	1.33	CONF
BSA	66,000	N/A	N/A	0.75	CONF
SOD-1 (Dimer)	32,000	N/A	N/A	N/A	CONF
Catalase (Tetramer)	240,000	N/A	N/A	N/A	CONF
Other					
D-Glucose	180	17,500	25,000	N/A	N/A
HEPES	238	15,000	10,000	N/A	N/A
Hypoxanthine	136	17.5	N/A	N/A	N/A
Linoleic Acid	280	0.16	N/A	N/A	N/A
Phenol Red, Sodium Salt	354	23	23	N/A	N/A
Putrescine·2HCl	161	0.5	N/A	N/A	N/A
Pyruvic Acid·Na	111	500	225	N/A	N/A
DL-Thioctic Acid	206	0.5	N/A	N/A	N/A
Thymidine	242	1.5	N/A	N/A	N/A
Progesterone	314	N/A	N/A	0.02	CONF
Sodium Selenite	173	N/A	N/A	0.02	CONF
Putrescine	88	N/A	N/A	180	CONF
Lecithin	770	N/A	N/A	N/A	CONF
Linolenic Acid	278	N/A	N/A	N/A	CONF
Phosphatidylcholine	776	N/A	N/A	N/A	CONF
Retinol	286	N/A	N/A	N/A	CONF
Retinyl Acetate	328	N/A	N/A	N/A	CONF
Sodium Selenite	173	N/A	N/A	N/A	CONF
T3 (Triodo-I-Thyronine)	650	N/A	N/A	N/A	CONF
DL-α-Trocopherol (Vitamin E)	431	N/A	N/A	N/A	CONF

DL-α-Trocopherol Acetate	473	N/A	N/A	N/A	CONF
L-Tyrosine·2Na·2H2O	263	200	400	N/A	N/A
L-Valine	117	450	800	N/A	N/A

Supplementary Table 2 | List of primers used for RT-qPCR (Related to Supplementary Fig. 23)

Below we summarized the forward (FWD) and reverse (REV) primers of the genes used for RT-qPCR in Supplementary Fig. 23.

Amotl2_FWD	CAGAGGGACAATGAGCGATTGC
Amotl2_REV	TCACGCTTGGAAGAGGTCCTCA
Bcl2_FWD	TGTGGATGACTGAGTACCTGAAC
Bcl2_REV	GAGAAATCAAACAGAGGTCGCATG
Cyr61_FWD	GGATGAATGGTGCCTTGC
Cyr61_REV	GTCCACATCAGCCCCTTG
Bax_FWD	GGGGCCTTTTTGCTACAGGG
Bax_Rev	AAAGATGGTCACTGTCTGCC
Bbc3_FWD	GTGTGGAGGAGGAGGAGTG
Bbc3_REV	TCGATGCTGCTCTTCTTGTC
Mdm2_FWD	GAAGGAGGAAACGCAGGACA
Mdm2_REV	CCTGGCAGATCACACATGGT
Gapdh_FWD	TGACCTCAACTACATGGTCTACA
Gapdh_REV	CTTCCCATTCTCGGCCTTG
FGF4_Fwd	AAGGCACCTGCCCTGTTCTG
FGF4_Rev	GGGAGCTAGCTGGCTGAAGAAA
FGF5_Fwd	AACTCCATGCAAGTGCCAAAT
FGF5_Rev	CGGACGCATAGGTATTATAGCTG
FGFR1_Fwd	TGGACCGCATTGTGGCCTTGAC
FGFR1_Rev	TCAGCGCCGTTTGAGTCCACTG
FGFR2_Fwd	TGCACGCAGGATGGACCTCTCT
FGFR2_Rev	TGCTCCTCGGGGACACGGTTAA
FGFR3_Fwd	AAGCCAGCCAGCTGCACACA
FGFR3_Rev	TCAAACGGCACGGAGAGGTCCA
FGFR4_Fwd	TAATGAGGGAGTGCTGGCACGC
FGFR4_Rev	AGGGTCGTGGCTGAAAACCGAG

SUPPLEMENTARY NOTES

Derivation of conditions required for millimeter-scale diffusion (related to Fig. 5)

Our experiments show that the cells secrete molecules that diffuse on the order of several millimeters. In this section, we use calculations to show that physics of diffusion also allows for biomolecules to diffuse on the order of millimeters or longer. At first, a millimeter-scale diffusion may seem counterintuitive. This is because one often does not think about two cells, 1 mm apart, communicating with each other via an exchange of diffusible molecules. But physics allows for such communication with diffusible molecules whose molecular weights are in the weight-range that we experimentally identified (Extended Data Fig. 1). Consider the general form of reaction-diffusion equation for the concentration *c* of a secreted molecule at position $\vec{r}$ and time *t*:

$$\frac{\partial c}{\partial t} = D\nabla^2 c(\vec{r},t) - \gamma c(\vec{r},t) \qquad \text{(Equation S1)}$$

where *D* and γ are the molecule's diffusion constant and degradation rate respectively. A molecule's diffusion length *L* is the characteristic (typical) distance that it travels before degrading. Let us first deduce *L* from dimensional analysis and then obtain its exact value from solving Equation S1. The only parameters in Equation S1 are *D* and γ. *D* is in a unit of length^2/time and γ is in a unit of 1/time. Hence, we have the well-known, $L = \sqrt{D/\gamma}$,. We also have,

$$\gamma = \frac{\log(2)}{\tau} \qquad \text{(Equation S2)}$$

where τ is the molecule's half-life. Next, as is common for biomolecules, we can estimate *D* with the Stokes-Einstein relationship by assuming that the diffusing molecule is spherical. We would then have

$$D = \frac{kT}{6\pi\eta r} \qquad \text{(Equation S3)}$$

where *k* is the Boltzmann constant, *T* is temperature, η is the dynamic viscosity of the medium in which the molecule is diffusing, and *r* is the radius of the diffusing, spherical particle. The ES cells grew at 37 ^{O}C and in an aqueous medium. Thus, for an order of magnitude estimate, we can use the dynamic viscosity of water at 40 ^{O}C: $\eta = 0.653 \times 10^{-3} N \cdot s/m^2$ (http://www.engineersedge.com). This value nearly stays the same at 30 ^{O}C and is thus insensitive to temperature changes for our purpose. Then, at 37 ^{O}C, we have

$$D = \frac{3.5 \times 10^{-19}\ m^3/s}{r} \qquad \text{(Equation S4)}$$

Before estimating the *r*, we can estimate the maximum diffusion constant D_{max} for our molecules of interest. Our experiments with molecular filters showed that the secreted protein(s) must be about ~100 kDa (specifically, between 50 to 300 kDa (Extended Data Fig. 1); or as low as ~25 kDa if we take the conservative estimate of $\pm$50% error in the filter-pore sizes (Supplementary Fig. 15)). Each amino acid has a mass of ~110 Da and end-to-end length of ~0.35 nm. Thus, our proteins of interest (of 100 kDa) would consist of ~1000 amino acids which, when joined stretched end-to-end, have a length of 350 nm. This clearly is a gross overestimate of the *r* for secreted proteins. With this overestimate, we have $D_{max} = 1\ \mu m^2/s$, which is the conservative, lower bound given for the diffusion constant of large biomolecular machines such as ribosomes. In fact, the conventional values assigned to the diffusion constant of typical proteins inside a cytoplasm fall in the range of 5 - 50 $\mu m^2/s$ (from BioNumbers - Milo et al. *Nucleic Acid Res* (2010)). Since the cytoplasm is a highly crowded environment that limits diffusion, we can expect a higher value for the protein of our interest that diffuses in the liquid medium.

We now estimate the *D*. The typical value of *r* for proteins is ~5 nm (from BioNumbers). In contrast, *r* ~ 30 nm for eukaryotic ribosomes (from BioNumbers), which are macromolecular complexes that are certainly heavier and larger than our proteins of interest as our experiments revealed. As a conservative estimate (i.e., to estimate a reasonable lower bound on the diffusion length), suppose that *r* ~ 20 nm for our proteins of interest, four times larger than the value of *r* for typical proteins (~5 nm). Then according to above equation, we have $D \approx 20\ \mu m^2/s$ which is reasonable in the ES cell's relatively non-viscous liquid medium (recall that 5 - 50 $\mu m^2/s$ for a crowded cytoplasm). Note that

$$\tau = \frac{L^2 \log(2)}{D} \qquad \text{(Equation S5)}$$

Hence, for a diffusion length *of* 1 mm, we need τ ~ 12 hours. Note that this is a conservative, overestimate of the secreted molecule's half-life since we underestimated the diffusion constant by assuming that the molecule is larger than half the radius of a ribosome (i.e., assumed *r* ~ 20 nm). A more realistic estimate such as, for example, *r* ~ 10 nm, would yield τ ~ 6 hours. A more typical value that one assigns to *r* for proteins is *r* ~ 5 nm, which would yield τ ~ 3 hours. In summary, both the conservative and more realistic estimates yield protein half-lives, which are required for a near millimeter-scale diffusion, that are well within the typically cited values of protein half-lives. Crucially, we measured the half-lives of the secreted molecules, including FGF4, in the supernatants of our cell cultures (Supplementary Fig. 25-

26) and found that they are longer than the calculated half-lives here, meaning that the secreted molecules can, in fact, reach further than the 1-mm diffusion length we used in our calculation here.

Next, we use the steady-state solution to Equation S1 to arrive at the same conclusions as above. Consider a spherical cell of radius *R* that secretes a molecule at a constant rate η in an isotropic manner. The three-dimensional reaction-diffusion equation is

$$\frac{\partial c}{\partial t} = \frac{1}{r^2}\frac{\partial}{\partial r}\left(Dr^2\frac{\partial c}{\partial r}\right) - \gamma c + \frac{\eta}{4\pi R^2}\delta(r - R) \qquad \text{(Equation S6)}$$

where *c* is the concentration, *r* is radial distance from the center of the cell, and δ is the Dirac delta function. As one can verify, its steady-state solution is

$$c(r) = \frac{c_R R}{r} exp\left(-\frac{r - R}{\lambda}\right) \qquad \text{(Equation S7)}$$

where $\lambda = \sqrt{D/\gamma}$ and

$$c_R = \frac{\eta}{4\pi R^2}\frac{\gamma}{\lambda}\frac{1}{1 + \frac{\lambda}{R}} \qquad \text{(Equation S8)}$$

Note that c_R is the steady-state concentration on the surface of the cell. The exponential term in the steady-state solution tells us that the characteristic length (i.e., diffusion length) is exactly $\sqrt{D/\gamma}$, as we also found from dimensional analysis above. Thus, the conclusions based on the dimensional analysis holds true. Note that this was not guaranteed since there could have been a small, numerical factor that scaled the λ down by several orders of magnitude.
