# Supplementary material for: Macroscopic quorum sensing sustains differentiating embryonic stem cells
Source: Nat Chem Biol. 2023 Jan 12;19(5):596–606. doi: 10.1038/s41589-022-01225-x (PMC10154202; doi:10.1038/s41589-022-01225-x)
Supplement: Supplementary file 2 — Reporting Summary [file 41589_2022_1225_MOESM2_ESM.pdf]

nature research

Corresponding author(s): Hyun Youk

Last updated by author(s): Nov 5, 2022

Reporting Summary

Nature Research wishes to improve the reproducibility of the work that we publish. This form provides structure for consistency and transparency in reporting. For further information on Nature Research policies, see our Editorial Policies and the Editorial Policy Checklist.

Statistics

For all statistical analyses, confirm that the following items are present in the figure legend, table legend, main text, or Methods section.

n/a	Confirmed	
☐	☒	The exact sample size (*n*) for each experimental group/condition, given as a discrete number and unit of measurement
☐	☒	A statement on whether measurements were taken from distinct samples or whether the same sample was measured repeatedly
☒	☐	The statistical test(s) used AND whether they are one- or two-sided<br>*Only common tests should be described solely by name; describe more complex techniques in the Methods section.*
☒	☐	A description of all covariates tested
☒	☐	A description of any assumptions or corrections, such as tests of normality and adjustment for multiple comparisons
☐	☒	A full description of the statistical parameters including central tendency (e.g. means) or other basic estimates (e.g. regression coefficient) AND variation (e.g. standard deviation) or associated estimates of uncertainty (e.g. confidence intervals)
☒	☐	For null hypothesis testing, the test statistic (e.g. *F*, *t*, *r*) with confidence intervals, effect sizes, degrees of freedom and *P* value noted<br>*Give P values as exact values whenever suitable.*
☒	☐	For Bayesian analysis, information on the choice of priors and Markov chain Monte Carlo settings
☒	☐	For hierarchical and complex designs, identification of the appropriate level for tests and full reporting of outcomes
☐	☒	Estimates of effect sizes (e.g. Cohen's *d*, Pearson's *r*), indicating how they were calculated

Our web collection on statistics for biologists contains articles on many of the points above.

Software and code

Policy information about availability of computer code

Data collection	BD FACSDiVa 8.0; Andor IQ3; QuantStudio 5
Data analysis	MATLAB (R2016-R2020);<br>R Studio 3.5.1<br>FlowJo 8.0<br>ImageJ 1.53c;<br>Salmon-1.5.1<br>TopHat 2.1.1;<br>Bowtie 2<br>Samtools 1.16.1<br>Cufflinks 2.2.1<br>Cuffdiff 2<br>CummeRbund 2.7.2<br>BBDuk 36.85<br>PANTHER<br><br>MATLAB codes used for data analysis, modeling, and plotting are publicly available at GitHub:<br>https://github.com/youklab/Daneshpour-Stemcells-2022.

For manuscripts utilizing custom algorithms or software that are central to the research but not yet described in published literature, software must be made available to editors and reviewers. We strongly encourage code deposition in a community repository (e.g. GitHub). See the Nature Research guidelines for submitting code & software for further information.

Data

Policy information about availability of data

All manuscripts must include a data availability statement. This statement should provide the following information, where applicable:

- Accession codes, unique identifiers, or web links for publicly available datasets
- A list of figures that have associated raw data
- A description of any restrictions on data availability

Our RNA-Seq data are available on NCBI's Gene Expression Omnibus and are accessible through GEO Series accession number GSE157642. As a reference genome for our RNA-Seq analyses, we used the genome sequence of Mus musculus from UCSC (mm10) which is avilable here: https://genome.ucsc.edu/cgi-bin/hgGateway?db=mm10 . Every main data for this study are available at GitHub: https://github.com/youklab/Daneshpour-Stemcells-2022.

Field-specific reporting

Please select the one below that is the best fit for your research. If you are not sure, read the appropriate sections before making your selection.

☒ Life sciences ☐ Behavioural & social sciences ☐ Ecological, evolutionary & environmental sciences

For a reference copy of the document with all sections, see nature.com/documents/nr-reporting-summary-flat.pdf

Life sciences study design

All studies must disclose on these points even when the disclosure is negative.

Sample size	No sample size calculation was performed to predetermine sample size before any experiment (because our study does not use any human/clinical test subjects). Our experiments are on cell cultures. Therefore, as is the standard for experiments with cell cultures, in every experiment, we performed measurements on n biological replicates as indicated for each figure (n is at least 3 for every experiment; specific value is given in every figure). Error bars report the mean with s.e.m. for every experiment. As is the standard for cell-culture experiments like ours, n being larger than or equal to 3 is considered sufficient (we don't use or rely on p-values or any other statistical testing measures to draw our conclusions).
Data exclusions	We did not exclude data.
Replication	Every experiments was performed at least three independent times (precise value is reported for every data in our paper as "n = # biologically independent replicates" (# is at least 3 for every experiment). Moreover, reproducibility of the experimental findings was verified by having more than one person performing the same experiments but on different days with different batches of media, multiple vials of cells, and different cell lines.
Randomization	This is not relevant for our study because our study does not use any statistical hypothesis testing. We did not use human/clinical subjects and did not use any subjective testing. We reported all data from biological replicates. In any of our experiments which used only cells, the concept of "randomizing" cells does not make sense.
Blinding	Blinding was not necessary for our experiments because our study does not use any statistical hypothesis testing and no conclusions involved any biases (which is what blinding is used to prevent). There are no human/clinical subjects or subjective testing of any sort. Our experiments used cell cultures and we reported all the results from biological replicates. Mathematical modeling reproduced these features, further solidifying our conclusions.

Reporting for specific materials, systems and methods

We require information from authors about some types of materials, experimental systems and methods used in many studies. Here, indicate whether each material, system or method listed is relevant to your study. If you are not sure if a list item applies to your research, read the appropriate section before selecting a response.

Materials & experimental systems

n/a	Involved in the study	
☐	☒	Antibodies
☐	☒	Eukaryotic cell lines
☒	☐	Palaeontology and archaeology
☒	☐	Animals and other organisms
☒	☐	Human research participants
☒	☐	Clinical data
☒	☐	Dual use research of concern

Methods

n/a	Involved in the study	
☒	☐	ChIP-seq
☐	☒	Flow cytometry
☒	☐	MRI-based neuroimaging

Antibodies

Antibodies used	Mouse FGF4 ELISA Kit (ELISAGenie / Westburg, MOES00755); PathScan Phospho-YAP (Ser397) Sandwich ELISA Kit (Cell Signaling Technology, #57046).
Validation	For FGF4 ELISA Kit: Supplementary Fig. 24 shows that we validated that the antibody in the FGF4 ELISA kit specifically detected FGF4. Specifically, Supplementary Fig. 24 shows that we validated the antibody by serially diluting the reconstituted FGF4 into N2B27 medium (our cell-culture medium) and then checking that we obtained a sensible standard curve (Supplementary Fig. 24). The absorbance signals (at 450 nm) were sufficiently high relative to the lower detection-limit of the ELISA kit (Supplementary Fig. 24). Furthermore, we used another form of FGF4 to verify that the ELISA kit could detect both the endogenously secreted form of FGF4 and the reconstituted form of FGF4 (from R&D Systems, #7486-F4) as described in our Methods section and Supplementary Fig. 24. Furthermore, manufacturer's validation data are here: https://www.assaygenie.com/mouse-fgf4-fibroblast-growth-factor-4-elisa-kit-moes00755/<br><br>For Phospho-YAP Sandwich ELISA kit: antibody validated by the manufacturer (validation data shown here: https://www.cellsignal.com/products/elisa-kits/phospho-yap-ser397-sandwich-elisa-kit/57046). Moreover, antibody was validated by us in Supplementary Fig. 29A in which we show a standard curve with various concentrations of cell lysates that contain phosphorylated Yap1 (shows no signal when phosphorylated Yap1 is absent; increasing signal as the amount of phosphorylated Yap1 increases).

Eukaryotic cell lines

Policy information about cell lines

Cell line source(s)	E14Tg2a.IV (129/Ola) cell line --- from ATCC;<br>46C cell line -- gift from Austin Smith (referenced in Ying et al. Nature (2008))<br>Brachyury-eGFP cell line -- Gift from Valery Kouskoff (referenced in Fehling et al. Dev Biol (2003); Pearson et al. Stem Cell Rep (2015))
Authentication	These cell lines were karyotyped.<br>We reproduced the behaviors that the papers mentioned above reported (e.g., differentiation efficiency of 46C was comparable to the value reported by Ying et al. Nature (2008) when we used their differentiation protocol; same for Brachyury-eGFP and the E14 cell lines.
Mycoplasma contamination	We routinely tested all cell lines for presence of mycoplasma. There were no mycoplasma contaminations for every cell line and experiment reported in our paper.
Commonly misidentified lines<br>(See ICLAC register)	None: there are no misidentified cell lines.

Flow Cytometry

Plots

Confirm that:

☒ The axis labels state the marker and fluorochrome used (e.g. CD4-FITC).

☒ The axis scales are clearly visible. Include numbers along axes only for bottom left plot of group (a 'group' is an analysis of identical markers).

☒ All plots are contour plots with outliers or pseudocolor plots.

☒ A numerical value for number of cells or percentage (with statistics) is provided.

Methodology

Sample preparation	We collected cells using accutase, washed them with 1X PBS, resuspended them in 1X PBS + 4% FBS and kept them on ice before doing instant measurements with a flow cytometer. We used a BD FACSCelesta system with a High-Throughput Sampler (HTS) and lasers with the following wavelengths: 405 nm (violet), 488 nm (blue) and 561 nm (yellow/green).
Instrument	BD FACSCelesta with Hight-Throughput Sampler and three lasers: 405 nm (violet), 488 nm (blue), 561 nm (yellow/green)
Software	MATLAB R2016-R2020
Cell population abundance	We reported all ES cells that the flow cytometer detected; As a control, flowing PBS without any cells yielded negligible number of events in the gate settings that we used.
Gating strategy	Supplementary Fig. 1b shows the gating strategy. Specifically, we calibrated the FSC and SSC gates to detect only mouse ES cells (FSC-PMT = 231 V, SSC-PMT = 225 V, GFP-PMT = 476 V; as a control, flowing plain 1X PBS yielded no detected events). We measured the GFP fluorescence using the FIT-C channel. We analysed the flow-cytometry data using FlowJo and custom MATLAB script (MathWorks).

☒ Tick this box to confirm that a figure exemplifying the gating strategy is provided in the Supplementary Information.
